# Copper(II)-Catalyzed Direct C3 Chalcogenylation of Indoles

**DOI:** 10.3390/molecules30091870

**Published:** 2025-04-22

**Authors:** Liuyan Pan, Shengwei Chen, Dongfang Wu, Jian Shao, Xiaofeng Bao, Gong-Qing Liu

**Affiliations:** Nantong Key Laboratory of Small Molecular Drug Innovation, School of Pharmacy, Nantong University, Nantong 226019, China

**Keywords:** chalcogenation, indoles, selenylindoles, sulfenylindoles, CuBr_2_

## Abstract

3-Chalcogenylindoles serve as crucial building blocks in organic synthesis and pharmaceutical chemistry. Herein, we describe a simple and general synthesis of 3-chalcogenylindoles through the direct C–H chalcogenation of indoles using *N*-selenophthalimide and *N*-sulfenylsuccinimide as chalcogenation reagents in the presence of CuBr_2_ as the catalyst. The reactions were carried out in CH_2_Cl_2_ at room temperature under an air atmosphere with a low loading of catalyst, and a wide range of 3-selenylindoles and 3-thioindoles were obtained in good yields. Various functionalities, namely, methyl, methoxy, halo, ester, cyano, trifluoromethyl, and formyl groups on indoles, have shown amenability to the developed reaction. A mechanism involving the activation of the chalcogenation agent through CuBr_2_ coordination with the amide carbonyl group is proposed.

## 1. Introduction

**Figure 1:**
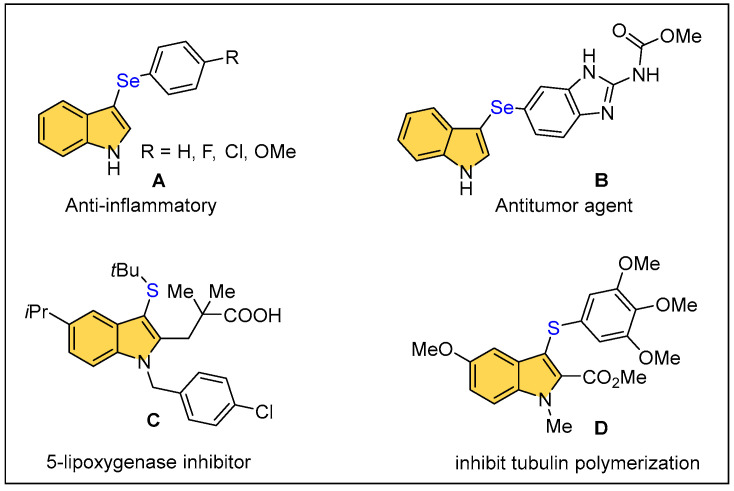
Examples of biologically active 3-chalcogenylindoles.

Currently, a “one drug, one target, one disease” paradigm is considered insufficient for addressing complex diseases, such as cancer and cardiovascular disorders [1,2,3,4]. Hence, the development of “synthetic multivalent molecules”, which integrate two or more bioactive moieties, presents an appealing and effective strategy for the design of novel therapeutic agents [5]. These hybrid molecules can synergistically enhance biological activity and improve interactions with multiple biological targets [6]. In this context, 3-chalcogenylindoles, which contain organochalcogenides (S, Se) and an indole skeleton, could be interesting targets for novel drug candidates and are anticipated to result in significantly greater biological activity than those containing only a single pharmacophore. Indeed, activity studies have shown that this class of compounds has a wide spectrum of therapeutic properties (Figure 1). For example, 3-selenylindoles **A** are highly reactive with oxidants and play a role in modulating oxidative and nitrosative damage at sites of inflammation [7]. In vitro model studies revealed that compound **B** significantly inhibits tubulin polymerization and disrupts tubulin-microtubule dynamics [8]. Alkylthioindole compound **C** has good inhibitory activity against 5-lipoxygenase and enhances the antitumor efficacy of celecoxib in human colon cancer cells [9]. Arylthioindole **D** significantly inhibits on tubulin polymerization and effectively suppresses the growth of human breast cancer cells [10].

**Scheme 1:**
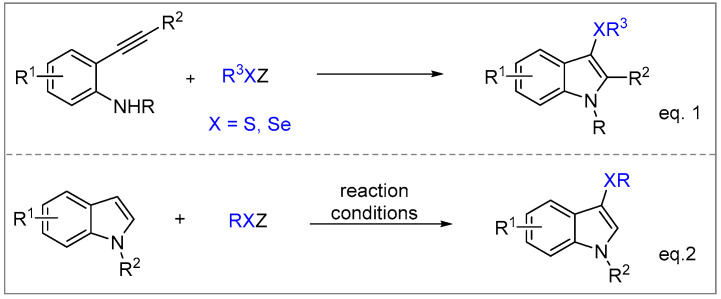
Deferent strategies for the synthesis of 3-chalcogenylindoles. Reaction conditions: (a) Base [14,15,16]: K_2_CO_3_, *t*BuOK, Cs_2_CO_3_, (b) Oxidant [17,18,19,20,21,22,23,24,25,26,27,28,29,30,31,32,33,34,35,36,37,38,39,40,41,42,43,44,45,46,47,48,49,50,51,52,53,54,55,56,57,58,59,60,61,62,63,64,65,66]: persulfate, I_2_/DMSO, TCCA, KIO_3_, Oxone, (c) Transition metal [27,28,29,30,31]: [Ag], [Cu], [Fe], [Bi], (d) Photo [32,33,34,35] or electrochemical methods [36,37].

Accordingly, over the years, a substantial amount of effort has been devoted to this topic, and numerous elegant methodologies for the construction of these chalcogenylindoles have been developed. Two major synthetic strategies are typically employed. One approach to synthesizing 3-chalcogenylindoles involves the cyclization of 2-alkynylanilines (Scheme 1. eq. 1) [11,12,13]. A more frequently utilized method is direct chalcogenylation of an existing indole ring using dichalcogenides. In these cases, various bases (Scheme 1. eq. 2a) [14,15,16], oxidants (Scheme 1. eq. 2b) [17,18,19,20,21,22,23,24,25,26], transition metals (Scheme 1. eq. 2c) [27,28,29,30,31], photoreactors [32,33,34,35] and electrolytic devices (Scheme 1. eq. 2d) [36,37] are employed to generate highly reactive species that react with nucleophilic indoles. Further, other sulfur or selenium reagents such as benzeneseleninic acids [38], thiophenols [39,40,41], sulfonyl hydrazides [42], sulfoximines [43] and sulfonyl chloride [44] have also been introduced for the chalcogenylation of indoles. Although some of these methods allow fast and efficient chalcogenation of indoles, they also present several limitations, including the use of costly and toxic transition metals or photocatalysts and the requirements for additional bases and ligands and high reaction temperatures. Thus, a mild and efficient general method for the chalcogenation (sulfenylation and selenation) of indoles is still necessary.

**Scheme 2:**
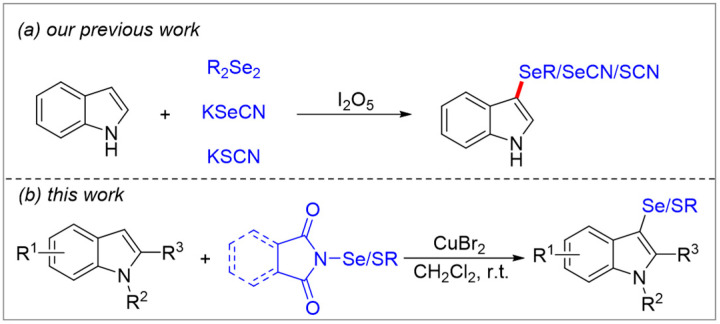
(**a**) Previous [48] and (**b**) present work to synthesize 3-chalcogenylindoles.

Recently, we are interested in developing a general and mild method for C–S/Se bond formation and have demonstrated that hypervalent iodine [45,46,47,48], N–F reagents [49,50], and photochemical processes [51,52,53] can effectively facilitate the cleavage of the E–E bond in dichalcogenides. Earlier, our group developed a method for the selenation and seleno/thiocyanation of indoles and activated/electron-rich arenes using stoichiometric amounts of iodine pentoxide (I_2_O_5_) (Scheme 2a) [48]. However, the major disadvantage associated with this method is the use of stoichiometric amounts of oxidant, which inevitably generates undesirable and toxic wastes and thus negatively impacts the environment, resulting in poor sustainability and cost efficiency. Furthermore, this method is not suitable for the synthesis of 3-sulfenylindoles.

In recent years, *N*-seleno and *N*-sulfenylsuccinimide/phthalimide have emerged as promising alternative chalcogenation reagents in organic synthesis [54,55,56]. These compounds are odorless, colorless crystalline solids that are more stable than toxic, unstable, and malodorous selenols and thiols. Although these reagents have been used for many years for the formation of new carbon–selenium and carbon–sulfur bonds, to the best of our knowledge, there is no report on the construction of organoselenium-substituted indoles, which remain both challenging and of great value. More recently, we reported Zn(OTf)_2_-catalyzed inter- and intramolecular selenofunctionalization of alkenes with electrophilic *N*-phenylselenophthalimide (*N*-PSP) to access various selenosubstituted heterocycles and vicinal Se heteroatom-disubstituted molecules [57]. Inspired by this result and the aforementioned background, we postulated that these reagents could be activated by abundant metals to generate electrophilic species, potentially serving as a suitable selenium/sulfur synthon for a selective C–H chalcogenation reaction of indoles. We herein report the CuBr_2_-catalyzed regioselective chalcogenylation of indole derivatives at the C3 position with *N*-selenophthalimide and *N*-sulfenylsuccinimide as a continuation of our interest in chalcogenation chemistry [45,46,47,48,49,50,51,52,53,57,58,59]. Notably, *N*-protected or *N*-unprotected indole derivatives were all suitable for this transformation, furnishing related products (Scheme 2b).

## 2. Results and Discussion

With these considerations in mind, we commenced our investigation with optimizing the conditions for the reaction of 1*H*-indole (**1a**) with *N*-PSP (**2a**). The results are summarized in Table 1. Based on our previous success with zinc-active *N*-PSP, we performed preliminary experiments with 5 mol% Zn(OTf)_2_ under ambient conditions with stirring for 5 h in CH_2_Cl_2_. To our delight, desired 3-phenylselanylindole **3a** was obtained in 75% yield (Table 1, entry 1). Other triflates, such as Cu(OTf)_2_, AgOTf, Yb(OTf)_3_ and In(OTf)_3_, were also investigated and reported similar results (entries 2–5). Further catalyst screening experiments indicated that CuBr_2_ was the most effective in terms of the reaction yield obtained (entries 6–10). Next, the influence of the solvent on the reaction using diverse solvents with different characteristics, including MeOH, DMF, EtOAc, CH_3_CN, and hexane, was also screened in the selenation reaction but did not result in higher yields (entries 11–15). The subsequent optimization revealed that reducing the loading of CuBr_2_ to 2 mol% did not affect the yield of product **3a**, whereas lowering the amount of catalyst to 1 mol% led to a significant decrease in yield (entries 16–17). Notably, the reaction can be scaled up to 5 mmol without a significant loss of efficacy. Specifically, 0.585 g of compound **1a** yielded 1.115 g of 3-indolyl selenide **3a** in a single run, corresponding to an 82% yield. Control experiments confirmed the necessity of the copper catalyst for the reaction, as the yield significantly decreased in its absence (entry 18).

**Scheme 3:**
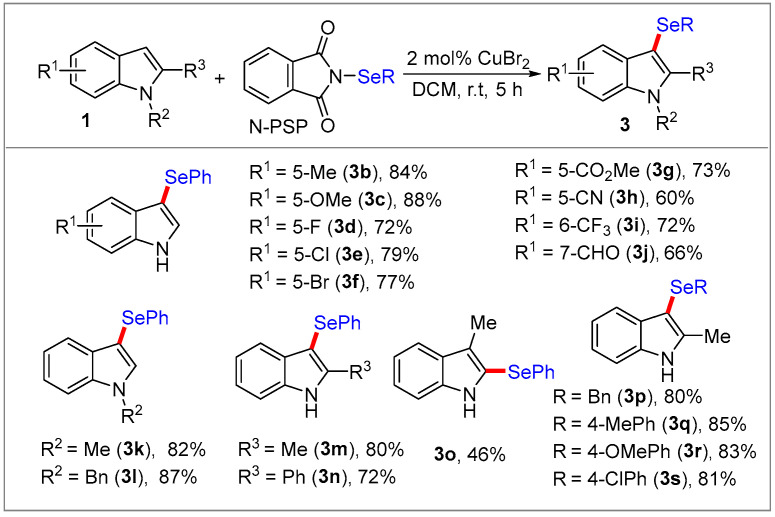
Substrate scope of 3-organoselenylindoles. Reaction conditions: **1** (0.2 mmol), *N*-PSP (0.2 mmol), CuBr_2_ (0.004 mmol) and CH_2_Cl_2_ (2 mL), air, r.t., 5 h.

With the optimal conditions in hand, we explored the substrate scope of this selenation approach. As shown in Scheme 3, indoles with electron-donating methyl and methoxy groups were well tolerated under the current conditions and provided methyl and methoxy-substituted indolyl selenides **3b**–**3c**. Similarly, halogen substituents, such as fluoro, chloro, and bromo groups on the phenyl ring of the indole, were compatible and afforded the respective 3-indolyl selenides **3d**–**3f** in 72–77% yields. Notably, numerous valuable functional groups, such as esters, cyanos, trifluoromethyls, and formyls, exhibited good tolerance within the reaction system (**3g**–**3j**), thus providing opportunities for further elaboration to create more complex products. Moreover, *N*-methyl and *N*-benzyl indole also reacted effectively with *N*-PSP to afford **3k** and **3l** in good yields. In addition, this study was extended to several substituted indoles where 2-methylindol and 2-phenylindole afforded their corresponding 3-phenylselenylindole **3m** and **3n** in yields of 80% and 72%, respectively. Interestingly, 3-methyl indole also coupled with *N*-PSP and resulted in 2-selenyl indole **3o** in 46% yield. The C-3 selenylation followed by a C-2 migration provides a plausible explanation for these results. Finally, other electrophilic selenylating agents, such as *N*-benzyl, *N*-4-methylphenyl, *N*-4-methoxyphenyl and *N*-4-chlorophthalimides, were also compatible with the catalytic protocol, resulting in **3p**–**3s** in good yields.

**Scheme 4:**
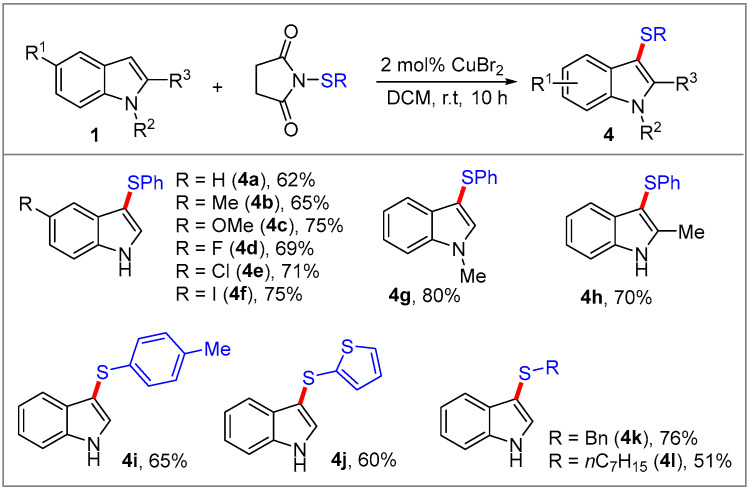
Substrate scope of indoles and *N*-thiosuccinimides. Reaction conditions: **1** (0.2 mmol), *N*-thiosuccinimide (0.2 mmol), CuBr_2_ (0.004 mmol) and CH_2_Cl_2_ (2 mL), air, r.t., 10 h.

The success of C–H selenation of indoles motivated us to explore sulfenylation under the optimized conditions. However, no reaction was observed when the sulfur analog *N*-sulfenyl phthalimide was employed. This outcome was attributed to the insufficient reactivity of *N*-sulfenyl phthalimide. After a preliminary screening of electrophilic thiolation reagents, we found that *N*-thiophenylsuccinimide effectively facilitates the sulfenylation of indoles in the presence of CuBr_2_ under ambient conditions. Despite the significant advancements in the application of *N*-thiosuccinimides as sulfenylation reagents [60,61,62,63], these methods have several drawbacks, including high catalyst loading, harsh reaction conditions and a narrow scope. Tudge et al. reported a reaction involving indoles with *N*-thioalkyl- and *N*-thioarylphthalimides to produce 3-thioindoles using a low concentration of MgBr_2_ catalyst (0.5 mol%). However, these reactions were conducted at 90 °C [60]. Thus, we expanded this methodology to synthesize various thioindoles, aiming to achieve C3 sulfenylation of indoles under mild conditions. As demonstrated in Scheme 4, *N*-thiophenylsuccinimide was reacted with a wide range of indoles to achieve the desired unsymmetrical diarylthioether (**4a**–**4h**). Different *N*-(thio)succinimides were next used to access a variety of C3-sulfenylated indoles. The use of N-thiosuccinimides containing electron-rich thioaryl moieties or heterocycles allowed reactions smoothly, yielding the thiolated product **4i**–**4j** in high yields. In addition, this method can also be applied to the thioalkylation of indole and the desired products **4k** and **4l** were obtained in acceptable yields when *N*-(benzylthio)succinimide and *N*-(heptylthio)succinimide were used as coupling reagents.

**Scheme 5:**
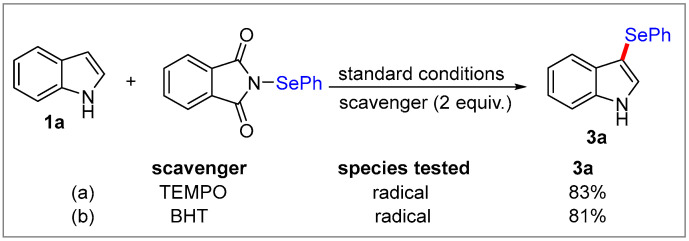
Control experiments used in mechanistic studies.

**Scheme 6:**
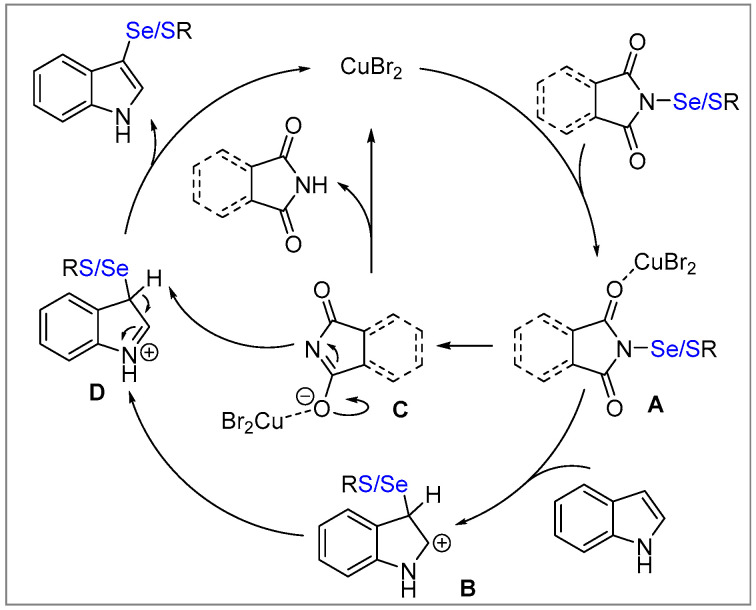
Plausible reaction mechanism.

We tried to follow the course of the reaction via NMR spectroscopy; however, it was unfruitful at this stage due to the presence of paramagnetic Cu(II) species. In fact, when we tried to study the complexation of reagents with CuBr_2_ by NMR spectroscopy, the signals observed were very broad, and no identifiable species could be discerned from the spectra. In addition, 2,2,6,6-tetramethylpiperidin-1-oxyl (TEMPO) (Scheme 5a) and 2,6-ditert-butyl-4-methylphenol (BHT) (Scheme 5b) were introduced as a radical trapping agent, and the reaction proceeded smoothly under the title conditions. This result confirmed that the reaction did not proceed through a radical mechanism. Therefore, we propose an electrophilic substitution mechanism. As depicted in Scheme 6, CuBr_2_ activates *N*-PSP or *N*-thiosuccinimide via coordination with the amide carbonyl group to form intermediate **A**. Then, the 3-position of the indole nucleophilically attacks **A**, forming complex **C** and intermediate **D**. Finally, aromatization of **D** to reform the indole ring occurs via deprotonation of **D** by the nitrogen atom of **C**, releasing the catalyst and resulting in the formation of chalcogenylindole and phthalimide/succinimide.

## 3. Materials and Methods

### 3.1. General Information

All chemicals were purchased as reagent grade and used without further purification. Solvents were dried and distilled prior to use. Petroleum ether used had a boiling point range of 60–90 °C. Chemical reactions were monitored using thin-layer chromatography (TLC) with precoated silica gel 60 plates of 0.25 mm thickness. Chromatographic purification of products was performed as flash column chromatography on silica gel (200–300 meshes). NMR spectra were recorded on a Bruker Avance-III HD (^1^H NMR: 400 MHz, ^13^C NMR: 100 MHz) spectrometer (Billerica, MS, USA) Chemical shifts are referenced to residual solvent signals (CDCl_3_: 7.26 ppm and 77.16 ppm for ^1^H NMR and ^13^C NMR, respectively) and reported in parts per million (ppm) relative to tetramethylsilane (TMS). Spin-spin coupling constants (J) were given in Hz. Melting points were determined on glass slides using a WRX-4 digital display microscopic (YiChe, Shanghai, China) melting point apparatus and were presented uncorrected.

### 3.2. General Procedure for the Synthesis of 3-Chalcogenylindoles

To a 20 mL test tube with magnetic stir bar were added 0.2 mmol indoles, 0.2 mmol *N*-selenophthalimide/*N*-sulfenylsuccinimide, 0.004 mmol CuBr_2_ and 2 mL of CH_2_Cl_2_. The reaction mixture was stirred at room temperature. The reaction was monitored by TLC. After completion of reaction, the solvent was removed with a rotary evaporator. The pure product was obtained by flash chromatography on silica gel using petroleum ether and ethyl acetate as the eluent.

**3-(Phenylselanyl)-1*H*-indole** (**3a**). Using the general procedure, the reaction was performed with the indole (1.0 equiv, 23 mg, 0.2 mmol), *N*-PSP (1.0 equiv, 60 mg, 0.2 mmol), CuBr_2_ (0.02 equiv, 0.89 mg, 4 μmol), and 2 mL of CH_2_Cl_2_. Compound **3a** was isolated as a yellow solid (47 mg, 86% yield) after flash chromatography (petroleum ether/ethyl acetate = 30/1). mp = 135–136 °C. (ref. [29] mp = 134–137 °C). ^1^H NMR (400 MHz, CDCl_3_): δ 8.40 (brs, 1H), 7.52 (d, *J* = 7.9 Hz, 1H), 7.37 (d, *J* = 2.5 Hz, 1H), 7.41 (d, *J* = 8.1 Hz, 1H), 7.21–7.00 (m, 7H). ^13^C{^1^H} NMR (100 MHz, CDCl_3_): δ 134.8, 133.1, 130.5, 129.2, 128.3, 127.4, 124.8, 122.3, 120.0, 118.9, 110.6, 97.5. The data are in accordance with the literature [48].

**5-Methyl-3-(phenylselanyl)-1*H*-indole** (**3b**). Using the general procedure, the reaction was performed with 5-methyl-1*H*-indole (1.0 equiv, 26 mg, 0.2 mmol), *N*-PSP (1.0 equiv, 60 mg, 0.2 mmol), CuBr_2_ (0.02 equiv, 0.89 mg, 4 μmol), and 2 mL of CH_2_Cl_2_. Compound **3b** was isolated as a brown solid (48 mg, 84% yield) after flash chromatography (petroleum ether/ethyl acetate = 20/1). mp = 93–95 °C. (ref. [25] mp = 134–137 °C). ^1^H NMR (400 MHz, CDCl_3_): δ 8.26 (brs, 1H), 7.44–7.35 (m, 2H), 7.26 (d, J = 8.3 Hz, 1H), 7.32–6.99 (m, 6H), 2.38 (s, 3H). ^13^C{^1^H} NMR (100 MHz, CDCl_3_): δ 134.3, 134.6, 132.0, 130.7, 129.9, 129.3, 128.7, 125.8, 124.2, 120.3, 110.7, 97.8, 21.1. The data are in accordance with the literature [48].

**5-Methoxy-3-(phenylselanyl)-1*H*-indole** (**3c**). Using the general procedure, the reaction was performed with 5-methoxy-1*H*-indole (1.0 equiv, 29 mg, 0.2 mmol), *N*-PSP (1.0 equiv, 60 mg, 0.2 mmol), CuBr_2_ (0.02 equiv, 0.89 mg, 4 μmol), and 2 mL of CH_2_Cl_2_. Compound **3c** was isolated as an oil (53 mg, 88% yield) after flash chromatography (petroleum ether/ethyl acetate = 8/1). ^1^H NMR (400 MHz, CDCl_3_): δ 8.41 (brs, 1H), 7.34 (d, *J* = 2.6 Hz, 1H), 7.32 (d, *J* = 8.8 Hz, 1H), 7.18 (dt, *J* = 6.0, 1.5 Hz, 2H), 7.19–7.10 (m, 4H), 6.87 (dd, *J* = 8.8, 2.5 Hz, 1H), 3.81 (s, 3H). ^13^C{^1^H} NMR (100 MHz, CDCl_3_): δ 154.7, 133.6, 132.4, 131.7, 130.5, 128.6, 128.9, 125.3, 113.7, 111.9, 101.8, 97.2, 56.2. The data are in accordance with the literature [48].

**5-Fluoro-3-(phenylselanyl)-1*H*-indole** (**3d**). Using the general procedure, the reaction was performed with 5-fluoro-1*H*-indole (1.0 equiv, 27 mg, 0.2 mmol), *N*-PSP (1.0 equiv, 60 mg, 0.2 mmol), CuBr_2_ (0.02 equiv, 0.89 mg, 4 μmol), and 2 mL of CH_2_Cl_2_. Compound **3d** was isolated as a pink solid (42 mg, 72% yield) after flash chromatography (petroleum ether/ethyl acetate = 15/1). mp = 116–118 °C. (ref. [29] mp = 118–120 °C). ^1^H NMR (400 MHz, CDCl_3_): δ 8.38 (brs, 1H), 7.54 (d, *J* = 2.5 Hz, 1H), 7.37 (dd, *J* = 8.8, 4.2 Hz, 1H), 7.22 (dd, *J* = 9.3, 2.5 Hz, 1H), 7.22–7.10 (m, 5H) (td, *J* = 9.0, 2.6 Hz, 1H). ^13^C{^1^H} NMR (100 MHz, CDCl_3_): δ 158.3 (d, *J* = 236.5 Hz), 133.7, 133.4, 133.1, 131.3 (d, *J* = 10.2 Hz), 129.4, 128.6, 126.1, 111.8 (d, *J* = 9.6 Hz), 111.8 (d, *J* = 26.7 Hz), 105.0 (d, *J* = 24.2 Hz), 98.7 (d, *J* = 4.8 Hz). ^19^F NMR (376 MHz, CDCl_3_): δ −122.4. The data are in accordance with the literature [48].

**5-Chloro-3-(phenylselanyl)-1*H*-indole** (**3e**). Using the general procedure, the reaction was performed with 5-chloro-1*H*-indole (1.0 equiv, 30 mg, 0.2 mmol), *N*-PSP (1.0 equiv, 60 mg, 0.2 mmol), CuBr_2_ (0.02 equiv, 0.89 mg, 4 μmol), and 2 mL of CH_2_Cl_2_. Compound **3e** was isolated as a white solid (49 mg, 79% yield) after flash chromatography (petroleum ether/ethy acetate = 18/1). mp = 113–114 °C. (ref. [26] mp = 109–111 °C). ^1^H NMR (400 MHz, CDCl_3_): δ 8.37 (brs, 1H), 7.58 (d, *J* = 2.1 Hz, 1H), 7.50 (d, *J* = 2.3 Hz, 1H), 7.28 (d, *J* = 8.6 Hz, 1H), 7.20–7.13 (m, 3H), 7.13–7.10 (m, 3H). ^13^C{^1^H} NMR (100 MHz, CDCl_3_): δ 133.2, 133.7, 132.2, 131.7, 128.9, 128.3, 127.2, 126.4, 123.0, 120.2, 112.8, 98.4. The data are in accordance with the literature [48].

**5-Bromo-3-(phenylselanyl)-1*H*-indole** (**3f**). Using the general procedure, the reaction was performed with 5-bromo-1*H*-indole (1.0 equiv, 39 mg, 0.2 mmol), *N*-PSP (1.0 equiv, 60 mg, 0.2 mmol), CuBr_2_ (0.02 equiv, 0.89 mg, 4 μmol), and 2 mL of CH_2_Cl_2_. Compound **3f** was isolated as a white solid (54 mg, 77% yield) after flash chromatography (petroleum ether/ethyl acetate = 15/1). mp = 130–132 °C. (ref. [26] mp = 136–138 °C). ^1^H NMR (400 MHz, CDCl_3_): δ 8.41 (brs, 1H), 7.74 (d, *J* = 1.7 Hz, 1H), 7.51 (d, *J* = 2.5 Hz, 1H), 7.33–7.24 (m, 2H), 7.21–7.14 (m, 2H), 7.22–7.11 (m, 3H). ^13^C{^1^H} NMR (100 MHz, CDCl_3_): δ 134.7, 133.6, 132.0, 132.3, 129.5, 128.3, 125.6, 126.3, 123.4, 114.7, 113.3, 98.1. The data are in accordance with the literature [48].

**Methyl 3-(phenylselanyl)-1*H*-indole-5-carboxylate** (**3g**). Using the general procedure, the reaction was performed with methyl 1*H*-indole-5-carboxylate (1.0 equiv, 35 mg, 0.2 mmol), *N*-PSP (1.0 equiv, 60 mg, 0.2 mmol), CuBr_2_ (0.02 equiv, 0.89 mg, 4 μmol), and 2 mL of CH_2_Cl_2_. Compound **3g** was isolated as a white solid (48 mg, 73% yield) after flash chromatography (petroleum ether/ethyl acetate = 3/1). mp = 155–157 °C. (ref. [23] mp = 165–168 °C). ^1^H NMR (400 MHz, CDCl_3_): δ 8.69 (s, 1H), 8.38 (d, *J* = 1.7 Hz, 1H), 8.02 (dd, *J* = 8.6, 1.7 Hz, 1H), 7.51 (d, *J* = 2.5 Hz, 1H), 7.49–7.37 (m, 1H), 7.24–7.13 (m, 2H), 7.23–6.96 (m, 3H), 3.88 (s, 3H). ^13^C{^1^H} NMR (100 MHz, CDCl_3_): δ 168.4, 139.3, 133.7, 132.4, 130.1, 128.8, 129.3, 126.0, 124.7, 123.6, 122.7, 111.5, 100.1, 51.8. The data are in accordance with the literature [48].

**3-(Phenylselanyl)-1*H*-indole-5-carbonitrile** (**3h**). Using the general procedure, the reaction was performed with 1*H*-indole-5-carbonitrile (1.0 equiv, 28 mg, 0.2 mmol), *N*-PSP (1.0 equiv, 60 mg, 0.2 mmol), CuBr_2_ (0.02 equiv, 0.89 mg, 4 μmol), and 2 mL of CH_2_Cl_2_. Compound **3h** was isolated as a white solid (36 mg, 60% yield) after flash chromatography (petroleum ether/ethyl acetate = 8/1). mp = 151–152 °C. (ref. [64] mp = 141–143 °C)^1^H NMR (400 MHz, CDCl_3_): δ 8.91 (brs, 1H), 8.04 (d, *J* = 1.2 Hz, 1H), 7.58 (d, *J* = 2.5 Hz, 1H), 7.51–7.39 (m, 2H), 7.25–7.16 (m, 2H), 7.23–7.10 (m, 3H). ^13^C{^1^H} NMR (100 MHz, CDCl_3_): δ 138.5, 133.4, 132.2, 129.7, 129.2, 129.2, 125.8, 126.6, 126.1, 120.7, 112.8, 103.8, 100.0. The data are in accordance with the literature [48].

**3-(Phenylselanyl)-6-(trifluoromethyl)-1*H*-indole** (**3i**). Using the general procedure, the reaction was performed with 6-(trifluoromethyl)-1*H*-indole (1.0 equiv, 37 mg, 0.2 mmol), *N*-PSP (1.0 equiv, 60 mg, 0.2 mmol), CuBr_2_ (0.02 equiv, 0.89 mg, 4 μmol), and 2 mL of CH_2_Cl_2_. Compound **3i** was isolated as a brown solid (49 mg, 72% yield) after flash chromatography (petroleum ether/ethyl acetate = 5/1). mp = 110–111 °C. ^1^H NMR (400 MHz, CDCl_3_): δ 8.57 (brs, 1H), 7.86–7.62 (m, 2H), 7.62 (d, *J* = 2.6 Hz, 1H), 7.42 (dd, *J* = 8.5, 1.4 Hz, 1H), 7.31–7.14 (m, 2H), 7.21–6.99 (m, 3H). ^13^C{^1^H} NMR (100 MHz, CDCl_3_): δ 135.5, 133.1, 133.5, 132.0, 128.8, 129.3, 126.6, 125.7, 125.5 (q, *J*_C-F_ = 31.9 Hz), 124.0, 121.4, 117.2 (d, *J*_C-F_ = 3.5 Hz), 108.8 (q, *J*_C-F_ = 4.5 Hz), 99.3. ^19^F NMR (376 MHz, CDCl_3_): δ −60.3. The data are in accordance with the literature [48].

**3-(Phenylselanyl)-1*H*-indole-7-carbaldehyde** (**3j**). Using the general procedure, the reaction was performed with 1*H*-indole-7-carbaldehyde (1.0 equiv, 29 mg, 0.2 mmol), *N*-PSP (1.0 equiv, 60 mg, 0.2 mmol), CuBr_2_ (0.02 equiv, 0.89 mg, 4 μmol), and 2 mL of CH_2_Cl_2_. Compound **3j** was isolated as a white solid (40 mg, 66% yield) after flash chromatography (petroleum ether/ethyl acetate = 10/1). mp = 158–160 °C. ^1^H NMR (400 MHz, CDCl_3_): δ 10.42 (brs, 1H), 10.08 (s, 1H), 7.96 (d, *J* = 7.8 Hz, 1H), 7.67 (dd, *J* = 7.3, 1.0 Hz, 1H), 7.58 (d, *J* = 2.4 Hz, 1H), 7.32 (t, *J* = 7.6 Hz, 1H), 7.22–7.15 (m, 2H), 7.13–6.98 (m, 3H). ^13^C{^1^H} NMR (100 MHz, CDCl_3_): δ 193.7, 134.0, 133.5, 133.4, 131.5, 130.1, 128.7, 129.0, 128.1, 126.4, 121.3, 120.2, 99.2. The data are in accordance with the literature [48].

**1-Methyl-3-(phenylselanyl)-1*H*-indole** (**3k**). Using the general procedure, the reaction was performed with 1-methyl-1*H*-indole (1.0 equiv, 26 mg, 0.2 mmol), *N*-PSP (1.0 equiv, 60 mg, 0.2 mmol), CuBr_2_ (0.02 equiv, 0.89 mg, 4 μmol), and 2 mL of CH_2_Cl_2_. Compound **3k** was isolated as a brown solid (47 mg, 82% yield) after flash chromatography (petroleum ether/ethyl acetate = 30/1). mp = 85–87 °C. (ref. [29] mp = 66–68 °C). ^1^H NMR (400 MHz, CDCl_3_): δ 7.57 (d, *J* = 8.5 Hz, 1H), 7.41 (d, *J* = 8.3 Hz, 1H), 7.31–7.24 (m, 4H), 7.24–7.09 (m, 4H), 3.86 (s, 3H). ^13^C{^1^H} NMR (100 MHz, CDCl_3_): δ 137.1, 136.1, 139.8, 131.1, 129.3, 128.2, 125.1, 122.7, 120.9, 120.2, 110.0, 95.6, 32.8. T The data are in accordance with the literature [48].

**1-Benzyl-3-(phenylselanyl)-1*H*-indole** (**3l**). Using the general procedure, the reaction was performed with 1-benzyl-1*H*-indole (1.0 equiv, 41 mg, 0.2 mmol), *N*-PSP (1.0 equiv, 60 mg, 0.2 mmol), CuBr_2_ (0.02 equiv, 0.89 mg, 4 μmol), and 2 mL of CH_2_Cl_2_. Compound **3l** was isolated as a white solid (63 mg, 87% yield) after flash chromatography (petroleum ether/ethyl acetate = 60/1). mp = 75–77 °C. (ref. [29] mp = 77–79 °C). ^1^H NMR (400 MHz, CDCl_3_): δ 7.67 (d, *J* = 7.8 Hz, 1H), 7.38 (s, 1H), 7.41–7.30 (m, 4H), 7.31–7.23 (m, 3H), 7.16–7.00 (m, 6H), 5.41 (s, 2H). ^13^C{^1^H} NMR (100 MHz, CDCl_3_): δ 136.7, 137.1, 134.6, 134.5, 131.3, 129.4, 129.2, 128.3, 128.3, 127.5, 125.2, 122.4, 121.1, 120.9, 109.8, 97.2, 50.7. The data are in accordance with the literature [48].

**2-Methyl-3-(phenylselanyl)-1*H*-indole** (**3m**). Using the general procedure, the reaction was performed with 2-methyl-1*H*-indole (1.0 equiv, 26 mg, 0.2 mmol), *N*-PSP (1.0 equiv, 60 mg, 0.2 mmol), CuBr_2_ (0.02 equiv, 0.89 mg, 4 μmol), and 2 mL of CH_2_Cl_2_. Compound **3m** was isolated as a pink solid (46 mg, 80% yield) after flash chromatography (petroleum ether/ethyl acetate = 20/1). mp = 90–91 °C. (ref. [29] mp = 99–100 °C). ^1^H NMR (400 MHz, CDCl_3_): δ 8.30 (brs, 1H), 7.51 (d, *J* = 7.7 Hz, 1H), 7.36 (d, *J* = 7.7 Hz, 1H), 7.19–6.92 (m, 7H), 2.57 (s, 3H). ^13^C{^1^H} NMR (100 MHz, CDCl_3_): δ 141.1, 136.2, 134.3, 131.5, 129.4, 128.6, 125.1, 122.5, 121.0, 120.3, 110.7, 96.7, 12.8. The data are in accordance with the literature [48].

**2-Phenyl-3-(phenylselanyl)-1*H*-indole** (**3n**). Using the general procedure, the reaction was performed with 2-phenyl-1*H*-indole (1.0 equiv, 38 mg, 0.2 mmol), *N*-PSP (1.0 equiv, 60 mg, 0.2 mmol), CuBr_2_ (0.02 equiv, 0.89 mg, 4 μmol), and 2 mL of CH_2_Cl_2_. Compound **3n** was isolated as an oil (50 mg, 72% yield) after flash chromatography (petroleum ether/ethyl acetate = 15/1). ^1^H NMR (400 MHz, CDCl_3_): δ 8.58 (s, 1H), 7.67 (d, *J* = 6.8 Hz, 2H), 7.69 (d, *J* = 7.9 Hz, 1H), 7.52–7.29 (m, 4H), 7.28–6.98 (m, 7H). ^13^C{^1^H} NMR (100 MHz, CDCl_3_): δ 141.8, 136.5, 134.4, 131.7, 131.9, 129.5, 129.1, 128.9, 128.7, 125.1, 123.6, 120.7, 121.3, 111.4, 96.0. The data are in accordance with the literature [48].

**3-Methyl-2-(phenylselanyl)-1*H*-indole** (**3o**). Using the general procedure, the reaction was performed with 3-methyl-1*H*-indole (1.0 equiv, 26 mg, 0.2 mmol), *N*-PSP (1.0 equiv, 60 mg, 0.2 mmol), CuBr_2_ (0.02 equiv, 0.89 mg, 4 μmol), and 2 mL of CH_2_Cl_2_. Compound **3o** was isolated as a brown solid (26 mg, 46% yield) after flash chromatography (petroleum ether/ethyl acetate = 10/1). mp = 82–83 °C (ref. [29] mp = 77–79 °C). ^1^H NMR (400 MHz, CDCl_3_): δ 8.00 (brs, 1H), 7.49 (d, *J* = 7.9 Hz, 1H), 7.18 (d, *J* = 7.9 Hz, 1H), 7.24–7.06 (m, 7H), 2.30 (s, 3H). ^13^C{^1^H} NMR (100 MHz, CDCl_3_): δ 136.2, 130.7, 128.5, 127.9, 127.4, 125.7, 121.9, 119.3, 118.7, 118.4, 116.7, 110.1, 9.7. The data are in accordance with the literature [48].

**3-(Benzylselanyl)-2-methyl-1*H*-indole** (**3p**). Using the general procedure, the reaction was performed with indole (1.0 equiv, 23 mg, 0.2 mmol), 2-(benzylselanyl)isoindoline-1,3-dione (1.0 equiv, 63 mg, 0.2 mmol), CuBr_2_ (0.02 equiv, 0.89 mg, 4 μmol), and 2 mL of CH_2_Cl_2_. Compound **3p** was isolated as an oil (48 mg, 80% yield) after flash chromatography (petroleum ether/ethyl acetate = 10/1). ^1^H NMR (400 MHz, CDCl_3_): δ 7.92 (brs, 1H), 7.71–7.59 (m, 1H), 7.31–7.22 (m, 1H), 7.14–7.09 (m, 2H), 7.15–7.10 (m, 3H), 7.10–6.92 (m, 2H), 3.77 (s, 2H), 2.06 (s, 3H). ^13^C{^1^H} NMR (100 MHz, CDCl_3_): δ 141.5, 149.7, 135.3, 131.6, 129.1, 127.8, 126.6, 121.3, 120.6, 120.0, 110.8, 97.1, 31.2, 12.6. 12.8. The data are in accordance with the literature [48].

**2-Methyl-3-(*p*-tolylselanyl)-1*H*-indole** (**3q**). Using the general procedure, the reaction was performed with indole (1.0 equiv, 23 mg, 0.2 mmol), 2-(*p*-tolylselanyl)isoindoline-1,3-dione (1.0 equiv, 63 mg, 0.2 mmol), CuBr_2_ (0.02 equiv, 0.89 mg, 4 μmol), and 2 mL of CH_2_Cl_2_. Compound **3q** was isolated as an oil (51 mg, 85% yield) after flash chromatography (petroleum ether/ethyl acetate = 15/1). ^1^H NMR (400 MHz, CDCl_3_): δ 8.21 (brs, 1H), 7.68–7.42 (m, 1H), 7.29 (d, *J* = 8.0 Hz, 1H), 7.23–7.06 (m, 4H), 6.89 (d, *J* = 7.9 Hz, 2H), 2.48 (s, 3H), 2.25 (s, 3H). ^13^C{^1^H} NMR (100 MHz, CDCl_3_): δ 141.1, 136.0, 135.6, 131.5, 139.7, 130.2, 129.0, 121.8, 120.4, 120.3, 110.7, 97.1, 21.3, 12.8. The data are in accordance with the literature [48].

**3-((4-Methoxyphenyl)selanyl)-2-methyl-1*H*-indole** (**3r**). Using the general procedure, the reaction was performed with indole (1.0 equiv, 23 mg, 0.2 mmol), 2-((4-methoxyphenyl)selanyl)isoindoline-1,3-dione (1.0 equiv, 66 mg, 0.2 mmol), CuBr_2_ (0.02 equiv, 0.89 mg, 4 μmol), and 2 mL of CH_2_Cl_2_. Compound **3r** was isolated as an oil (52 mg, 83% yield) after flash chromatography (petroleum ether/ethyl acetate = 10/1). ^1^H NMR (400 MHz, CDCl_3_): δ 8.25 (brs, 1H), 7.61 (d, *J* = 7.6 Hz, 1H), 7.32 (d, *J* = 7.6 Hz, 2H), 7.26–7.07 (m, 4H), 6.73 (d, *J* = 8.7 Hz, 2H), 3.67 (s, 3H), 2.51 (s, 3H). ^13^C{^1^H} NMR (100 MHz, CDCl_3_): δ 158.5, 140.7, 136.1, 131.4, 131.0, 123.4, 121.7, 120.8, 120.3, 115.1, 110.6, 97.2, 55.4, 13.5. The data are in accordance with the literature [48].

**3-((4-Chlorophenyl)selanyl)-2-methyl-1*H*-indole** (**3s**). Using the general procedure, the reaction was performed with indole (1.0 equiv, 23 mg, 0.2 mmol), 2-((4-chlorophenyl)selanyl)isoindoline-1,3-dione (1.0 equiv, 67 mg, 0.2 mmol), CuBr_2_ (0.02 equiv, 0.89 mg, 4 μmol), and 2 mL of CH_2_Cl_2_. Compound **3s** was isolated as a brown solid (52 mg, 81% yield) after flash chromatography (petroleum ether/ethyl acetate = 12/1). mp = 117–118 °C. (ref. [38] mp = 120–122 °C). ^1^H NMR (400 MHz, CDCl_3_): δ 8.32 (brs, 1H), 7.48 (d, *J* = 7.7 Hz, 1H), 7.30 (d, *J* = 8.1 Hz, 1H), 7.21 (dd, *J* = 8.1, 7.1 Hz, 1H), 7.09 (dd, *J* = 8.1, 7.1 Hz, 1H), 7.11 (s, 4H), 2.48 (s, 3H). ^13^C{^1^H} NMR (100 MHz, CDCl_3_): δ 141.3, 136.2, 132.6, 130.7, 131.3, 130.1, 128.8, 122.5, 121.2, 119.9, 110.4, 95.7, 13.5. 13.6. The data are in accordance with the literature [48].

**3-(Phenylthio)-1*H*-indole** (**4a**). Using the general procedure, the reaction was performed with indole (1.0 equiv, 23 mg, 0.2 mmol), 1-(phenylthio)pyrrolidine-2,5-dione (1.0 equiv, 41 mg, 0.2 mmol), CuBr_2_ (0.02 equiv, 0.89 mg, 4 μmol), and 2 mL of CH_2_Cl_2_. Compound **4a** was isolated as a brick red solid (28 mg, 62% yield) after flash chromatography (petroleum ether/ethyl acetate = 30/1). mp = 128–129 °C. (ref. [29] mp = 130–132 °C). ^1^H NMR (400 MHz, CDCl_3_): δ 8.27 (brs, 1H), 7.58 (dd, *J* = 7.9, 1.0 Hz, 1H), 7.38 (d, *J* = 2.6 Hz, 1H), 7.33 (dd, *J* = 8.2, 1.0 Hz, 1H), 7.23–7.12 (m, 1H), 7.17–6.99 (m, 6H). ^13^C{^1^H} NMR (100 MHz, CDCl_3_): δ 139.5, 136.1, 130.8, 128.7, 129.1, 126.3, 125.2, 122.7, 121.4, 120.0, 111.9, 103.3. The data are in accordance with the literature [29].

**5-Methyl-3-(phenylthio)-1*H*-indole** (**4b**). Using the general procedure, the reaction was performed with 5-methyl-1*H*-indole (1.0 equiv, 26 mg, 0.2 mmol), 1-(phenylthio)pyrrolidine-2,5-dione (1.0 equiv, 41 mg, 0.2 mmol), CuBr_2_ (0.02 equiv, 0.89 mg, 4 μmol), and 2 mL of CH_2_Cl_2_. Compound **4b** was prepared isolated as a light yellow solid (31 mg, 65% yield) after flash chromatography (petroleum ether/ethyl acetate = 40/1). mp = 145–146 °C. (ref. [39] mp = 134–135 °C) ^1^H NMR (400 MHz, CDCl_3_): δ 8.18 (brs, 1H), 7.44–7.36 (m, 2H), 7.28 (d, *J* = 8.3 Hz, 1H), 7.11 (t, *J* = 7.8 Hz, 2H), 7.10–6.92 (m, 4H), 2.29 (s, 3H). ^13^C{^1^H} NMR (100 MHz, CDCl_3_): δ 139.1, 135.3, 131.4, 129.9, 129.7, 128.2, 125.3, 124.9, 124.3, 118.9, 110.8, 102.4, 21.2. The data are in accordance with the literature [65].

**5-Methoxy-3-(phenylthio)-1*H*-indole** (**4c**). Using the general procedure, the reaction was performed with 5-methoxy-1*H*-indole (1.0 equiv, 29 mg, 0.2 mmol), 1-(phenylthio)pyrrolidine-2,5-dione (1.0 equiv, 41 mg, 0.2 mmol), CuBr_2_ (0.02 equiv, 0.89 mg, 4 μmol), and 2 mL of CH_2_Cl_2_. Compound **4c** was isolated as an oil (38 mg, 75% yield) after flash chromatography (petroleum ether/ethyl acetate = 10/1). ^1^H NMR (400 MHz, CDCl_3_): δ 8.31 (brs, 1H), 7.42 (d, *J* = 2.7 Hz, 1H), 7.21 (d, *J* = 8.8 Hz, 1H), 7.17–6.89 (m, 6H), 6.87 (dd, *J* = 8.8, 2.5 Hz, 1H), 3.68 (s, 3H). ^13^C{^1^H} NMR (100 MHz, CDCl_3_): δ 155.5, 138.8, 131.6, 131.4, 129.7, 128.2, 125.3, 125.0, 113.9, 112.1, 101.8, 101.2, 56.2. The data are in accordance with the literature [29].

**5-Fluoro-3-(phenylthio)-1*H*-indole** (**4d**). Using the general procedure, the reaction was performed with 5-fluoro-1*H*-indole (1.0 equiv, 27 mg, 0.2 mmol), 1-(phenylthio)pyrrolidine-2,5-dione (1.0 equiv, 41 mg, 0.2 mmol), CuBr_2_ (0.02 equiv, 0.89 mg, 4 μmol), and 2 mL of CH_2_Cl_2_. Compound **4d** was isolated as a light yellow solid (34 mg, 69% yield) after flash chromatography (petroleum ether/ethyl acetate =30/1). mp = 150–152 °C. (ref. [29] mp = 167–169 °C). ^1^H NMR (400 MHz, CDCl_3_): δ 8.37 (brs, 1H), 7.51 (d, *J* = 2.7 Hz, 1H), 7.29 (dd, *J* = 8.8, 4.2 Hz, 1H), 7.23–7.18 (m, 1H), 7.05 (dd, *J* = 8.4, 6.9 Hz, 2H), 7.08–7.01 (m, 3H), 6.98 (td, *J* = 9.0, 2.5 Hz, 1H). ^13^C{^1^H} NMR (100 MHz, CDCl_3_): δ 159.1 (d, *J*_C-F_ = 237.0 Hz), 139.0, 133.4, 132.6, 128.3, 126.2, 124.7, 112.8 (d, *J*_C-F_ = 9.5 Hz), 112.2, 112.0, 105.1 (d, *J*_C-F_ = 24.2 Hz). ^19^F NMR (376 MHz, CDCl_3_): δ -122.1. The data are in accordance with the literature [29].

**5-Chloro-3-(phenylthio)-1*H*-indole** (**4e**). Using the general procedure, the reaction was performed with 5-chloro-1*H*-indole (1.0 equiv, 30 mg, 0.2 mmol), 1-(phenylthio)pyrrolidine-2,5-dione (1.0 equiv, 41 mg, 0.2 mmol), CuBr_2_ (0.02 equiv, 0.89 mg, 4 μmol), and 2 mL of CH_2_Cl_2_. Compound **4e** was isolated as a light yellow solid (37 mg, 71% yield) after flash chromatography (petroleum ether/ethyl acetate = 30/1). mp = 115–116 °C. (ref. [44] mp = 111–113 °C). ^1^H NMR (400 MHz, CDCl_3_): δ 8.32 (brs, 1H), 7.47 (d, *J* = 1.9 Hz, 1H), 7.46 (d, *J* = 2.6 Hz, 1H), 7.33 (d, *J* = 8.6 Hz, 1H), 7.21–7.11 (m, 3H), 6.98 (d, *J* = 7.2 Hz, 3H). ^13^C{^1^H} NMR (100 MHz, CDCl_3_): δ 139.1, 135.0, 131.6, 130.8, 129.3, 126.8, 126.4, 125.5, 123.2, 119.6, 113.0, 103.4. The data are in accordance with the literature [65].

**5-Iodo-3-(phenylthio)-1*H*-indole** (**4f**). Using the general procedure, the reaction was performed with 5-iodo-1*H*-indole (1.0 equiv, 48 mg, 0.2 mmol), 1-(phenylthio)pyrrolidine-2,5-dione (1.0 equiv, 41 mg, 0.2 mmol), CuBr_2_ (0.02 equiv, 0.89 mg, 4 μmol), and 2 mL of CH_2_Cl_2_. Compound **4f** was isolated as a light yellow solid (53 mg, 75% yield) after flash chromatography (petroleum ether/ethyl acetate = 40/1). mp = 150–151 °C. (ref. [19] mp = 111–113 °C). ^1^H NMR (400 MHz, CDCl_3_): δ 8.41 (brs, 1H), 7.93 (d, *J* = 1.6 Hz, 1H), 7.42 (dd, *J* = 8.6, 1.7 Hz, 1H), 7.43 (d, *J* = 2.6 Hz, 1H), 7.24–7.04 (m, 6H). ^13^C{^1^H} NMR (100 MHz, CDCl_3_): δ 139.1, 135.2, 132.0, 131.4, 131.9, 129.3, 128.6, 126.1, 124.5, 113.9, 102.1, 85.2. The data are in accordance with the literature [65].

**1-Methyl-3-(phenylthio)-1*H*-indole** (**4g**). Using the general procedure, the reaction was performed with 1-methyl-1*H*-indole (1.0 equiv, 26 mg, 0.2 mmol), 1-(phenylthio)pyrrolidine-2,5-dione (1.0 equiv, 44 mg, 0.2 mmol), CuBr_2_ (0.02 equiv, 0.89 mg, 4 μmol), and 2 mL of CH_2_Cl_2_. Compound **4g** was isolated as a light yellow solid (38 mg, 80% yield) after flash chromatography (petroleum ether/ethyl acetate = 40/1). mp = 90–92 °C. (ref. [29] mp = 86–88 °C). ^1^H NMR (400 MHz, CDCl_3_): δ 7.49 (d, *J* = 7.9 Hz, 1H), 7.28 (d, *J* = 8.2 Hz, 1H), 7.24–7.20 (m, 2H), 7.16–7.01 (m, 5H), 7.01 (t, *J* = 7.0 Hz, 1H), 3.80 (s, 3H). ^13^C{^1^H} NMR (100 MHz, CDCl_3_): δ 140.2, 137.3, 134.8, 130.3, 128.9, 125.3, 124.5, 122.8, 120.1, 120.3, 110.2, 100.8, 32.9. The data are in accordance with the literature [29].

**2-Methyl-3-(phenylthio)-1*H*-indole** (**4h**). Using the general procedure, the reaction was performed with 2-methyl-1*H*-indole (1.0 equiv, 26 mg, 0.2 mmol), 1-(phenylthio)pyrrolidine-2,5-dione (1.0 equiv, 41 mg, 0.2 mmol), CuBr_2_ (0.02 equiv, 0.89 mg, 4 μmol), and 2 mL of CH_2_Cl_2_. Compound **4h** was isolated as a light yellow solid (33 mg, 70% yield) after flash chromatography (petroleum ether/ethyl acetate = 40/1). mp = 120–121 °C. (ref. [29] mp = 116–118 °C). ^1^H NMR (400 MHz, CDCl_3_): δ 8.08 (s, 1H), 7.51 (d, *J* = 7.7 Hz, 1H), 7.29 (d, *J* = 8.0 Hz, 1H), 7.14–6.96 (m, 4H), 7.02–6.93 (m, 3H), 2.39 (s, 3H). ^13^C{^1^H} NMR (100 MHz, CDCl_3_): δ 141.5, 138.9, 135.6, 130.0, 129.1, 125.2, 124.7, 121.8, 120.5, 118.8, 110.4, 99.6, 12.5. The data are in accordance with the literature [29].

**3-(*p*-Tolylthio)-1*H*-indole** (**4i**). Using the general procedure, the reaction was performed with the indole (1.0 equiv, 26 mg, 0.2 mmol), 1-(*p*-tolylthio)pyrrolidine-2,5-dione (1.0 equiv, 44 mg, 0.2 mmol), CuBr_2_ (0.02 equiv, 0.89 mg, 4 μmol), and 2 mL of CH_2_Cl_2_. Compound **4i** was isolated as a light yellow solid (31 mg, 65% yield) after flash chromatography (petroleum ether/ethyl acetate = 20/1). mp = 120–121 °C. (ref. [41] mp = 125–126 °C). ^1^H NMR (400 MHz, CDCl_3_): δ 8.37 (s, 1H), 7.52 (d, *J* = 7.9 Hz, 1H), 7.46 (d, *J* = 2.6 Hz, 1H), 7.41 (d, *J* = 8.1 Hz, 1H), 7.24–7.19 (m, 1H), 7.11 (t, *J* = 7.5 Hz, 1H), 6.99 (d, *J* = 8.2 Hz, 2H), 6.87 (d, *J* = 8.2 Hz, 2H), 2.21 (s, 3H). ^13^C{^1^H} NMR (100 MHz, CDCl_3_): δ 136.2, 135.7, 134.1, 130.6, 130.0, 128.8, 125.7, 122.9, 121.3, 119.3, 111.8, 103.1, 30.1. The data are in accordance with the literature [66].

**3-(Thiophen-2-ylthio)-1*H*-indole** (**4j**). Using the general procedure, the reaction was performed with the indole (1.0 equiv, 26 mg, 0.2 mmol), 1-(thiophen-2-ylthio)pyrrolidine-2,5-dione (1.0 equiv, 43 mg, 0.2 mmol), CuBr_2_ (0.02 equiv, 0.89 mg, 4 μmol), and 2 mL of CH_2_Cl_2_. Compound **4j** was isolated as a light yellow solid (28 mg, 60% yield) after flash chromatography (petroleum ether/ethyl acetate = 15/1). mp = 96–97 °C. (ref. [15] mp = 84–85 °C). ^1^H NMR (400 MHz, CDCl_3_): δ 8.18 (brs, 1H), 7.75 (dd, *J* = 7.7, 1.4 Hz, 1H), 7.39 (d, *J* = 2.6 Hz, 1H), 7.34 (dd, *J* = 7.6, 1.3 Hz, 1H), 7.123–7.14 (m, 2H), 7.11 (dd, *J* = 5.3, 1.2 Hz, 1H), 7.06 (dd, *J* = 3.6, 1.3 Hz, 1H), 6.77 (dd, *J* = 5.3, 3.6 Hz, 1H). ^13^C{^1^H} NMR (100 MHz, CDCl_3_): δ 138.2, 136.4, 130.1, 129.6, 128.7, 127.5, 126.8, 122.8, 121.3, 119.7, 111.1, 107.1. The data are in accordance with the literature [15].

**3-(Benzylthio)-1*H*-indole** (**4k**). Using the general procedure, the reaction was performed with the indole (1.0 equiv, 26 mg, 0.2 mmol), 1-(benzylthio)pyrrolidine-2,5-dione (1.0 equiv, 44 mg, 0.2 mmol), CuBr_2_ (0.02 equiv, 0.89 mg, 4 μmol), and 2 mL of CH_2_Cl_2_. Compound **4k** was isolated as a light yellow solid (36 mg, 76% yield) after flash chromatography (petroleum ether/ethyl acetate =20/1). mp = 79–80 °C. (ref. [41] mp = 84–85 °C). ^1^H NMR (400 MHz, CDCl_3_): δ 8.05 (brs, 1H), 7.70–7.49 (m, 1H), 7.32 (d, *J* = 7.9 Hz, 1H), 7.18–7.02 (m, 5H), 7.03 (dd, *J* = 7.3, 2.2 Hz, 2H), 6.96 (d, *J* = 2.5 Hz, 1H), 3.81 (s, 2H). ^13^C{^1^H} NMR (100 MHz, CDCl_3_): δ 138.1, 136.5, 130.2, 128.8, 128.2, 127.6, 127.4, 122.7, 121.2, 119.7, 111.3, 107.1, 30.0. The data are in accordance with the literature [29].

**3-(Heptylthio)-1*H*-indole** (**4l**). Using the general procedure, the reaction was performed with the indole (1.0 equiv, 26 mg, 0.2 mmol), 1-(heptylthio)pyrrolidine-2,5-dione (1.0 equiv, 46 mg, 0.2 mmol), CuBr_2_ (0.02 equiv, 0.89 mg, 4 μmol), and 2 mL of CH_2_Cl_2_. Compound **4l** was isolated as an oil (25 mg, 51% yield) after flash chromatography (petroleum ether/ethyl acetate = 20/1). ^1^H NMR (400 MHz, CDCl_3_): δ 8.15 (brs, 1H), 7.78–7.60 (m, 1H), 7.33 (d, *J* = 7.5 Hz, 1H), 7.26 (d, *J* = 2.5 Hz, 1H), 7.14–7.07 (m, 2H), 2.66 (t, *J* = 7.4 Hz, 2H), 1.48–1.39 (m, 2H), 1.33–1.20 (m, 2H), 1.25–1.14 (m, 6H), 0.82 (t, *J* = 6.7 Hz, 3H). ^13^C{^1^H} NMR (100 MHz, CDCl_3_): δ 136.6, 129.1, 129.4, 122.2, 120.9, 119.0, 111.1, 106.6, 35.9, 32.2, 30.3, 28.7, 28.9, 22.3, 14.5. The data are in accordance with the literature [67].

## 4. Conclusions

In summary, we have reported a simple, efficient and practical C3-chalcogenylation protocol for accessing a variety of 3-chalcogenylindoles. This method was achieved with readily available *N*-selenophthalimide and *N*-sulfenylsuccinimide as the chalcogenation reagents and CuBr_2_ as the catalyst. Moreover, the reactions required low catalyst loadings of an earth-abundant, nonprecious transition metal and were conducted at room temperature in air with a wide tolerance of functional groups. The simplicity of our approach, the low cost of the reagents, and the fact that no particular precautions to exclude moisture or oxygen from the reaction system need to be taken suggest that the present protocol could be useful in the preparation of organochalcogen compounds. New schemes for the construction of other bioactive molecules containing an organochalcogen moiety are in progress in our laboratories, and the results will be reported subsequently.

## Figures and Tables

**Table 1:** Optimization of the reaction conditions *^a^*.

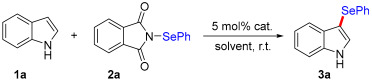			
Entry	cat.	Solvent	Isolated Yield (%)
1	Zn(OTf)_2_	DCM	75
2	Cu(OTf)_2_	DCM	70
3	AgOTf	DCM	62
4	Yb(OTf)_3_	DCM	71
5	In(OTf)_3_	DCM	75
6	ZnBr_2_	DCM	77
7	FeCl_3_	DCM	82
8	CaBr_2_	DCM	79
9	CuBr_2_	DCM	87
10	MnBr_2_	DCM	80
11	CuBr_2_	MeOH	53
12	CuBr_2_	DMF	44
13	CuBr_2_	EtOAc	71
14	CuBr_2_	CH_3_CN	63
15	CuBr_2_	hexane	47
16 *^b^*	CuBr_2_	DCM	86 (82) *^d^*
17 *^c^*	CuBr_2_	DCM	62
18	-	DCM	23

*^a^* Reaction conditions: **1a** (0.20 mmol, 1.00 equiv), **2a** (0.20 mmol, 1.00 equiv), cat. (0.010 mmol, 0.05 equiv), solvent (2 mL), r.t., 5 h. *^b^* 2 mol% CuBr_2_. *^c^* 1 mol% CuBr_2_. *^d^* 5 mmol.

## Data Availability

All data and material described in this work are available in this article or in the Supplementary Materials.

## Supplementary Materials

The following supporting information can be downloaded at: https://www.mdpi.com/article/10.3390/molecules30091870/s1, Figures S1–S65: Copies of NMR spectra for the obtained compounds.
