# Supplementary material for: Copper(II)-Catalyzed Direct C3 Chalcogenylation of Indoles
Source: Molecules. 2025 Apr 22;30(9):1870. doi: 10.3390/molecules30091870 (PMC12073016; doi:10.3390/molecules30091870)
Supplement: Supplementary file 1 [file molecules-30-01870-s001.zip › molecules-3523041-supplementary.pdf]

Supporting Information

Copper(II)-Catalyzed Direct C3 Chalcogenylation of Indoles

Liuyan Pan, Shengwei Chen, Dongfang Wu, Jian Shao *, Xiaofeng Bao * and Gong-Qing Liu *

Nantong Key Laboratory of Small Molecular Drug Innovation, School of Pharmacy, Nantong University, Nantong 226019, China

E-mail: shaojian@ntu.edu.cn (J.S.), baoxi@ntu.edu.cn (X.B.), gqliu@ntu.edu.cn (G.-Q.L.)

Content

1. Copies of NMR spectra..S2

1. Copies of NMR spectra

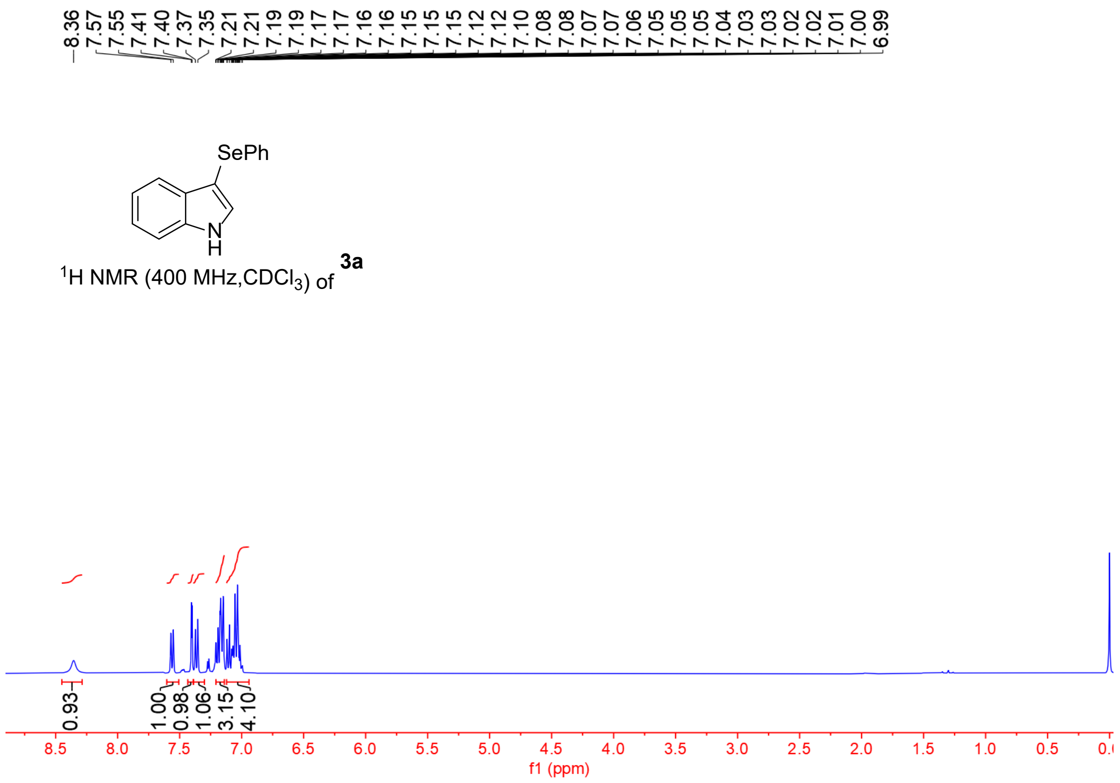

Figure S1: ^{1}H NMR spectrum for compound **3a**

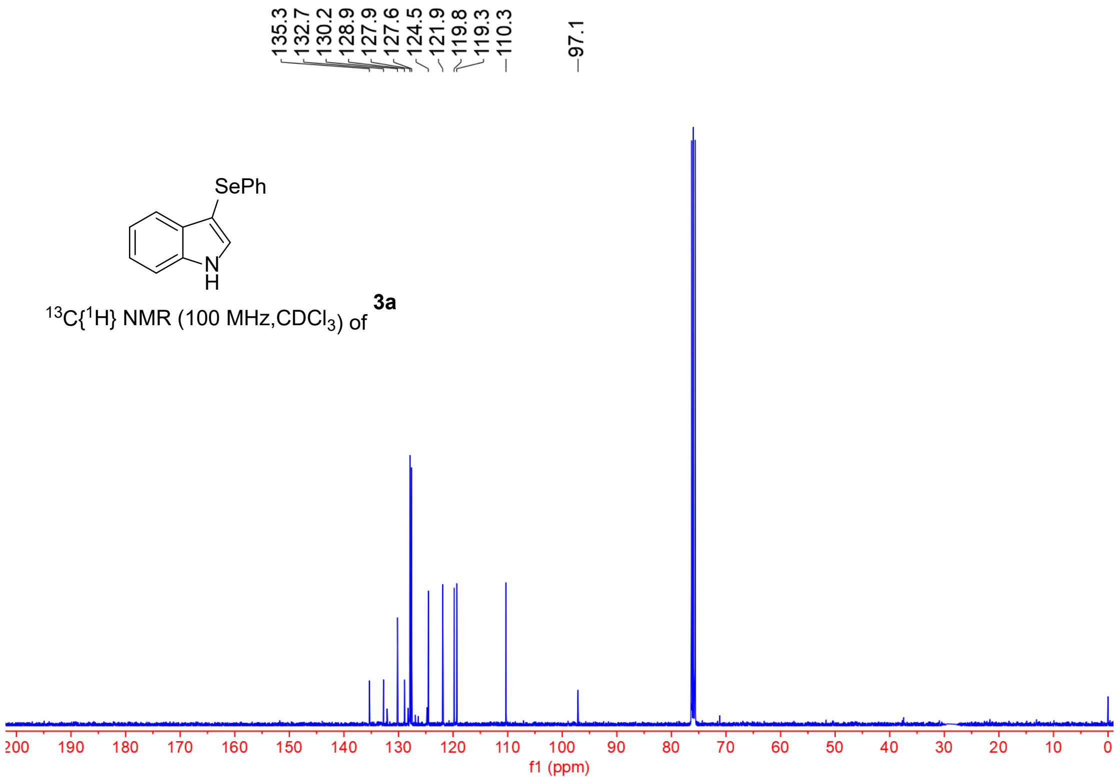

Figure S2: ^{13}C NMR spectrum for compound **3a**

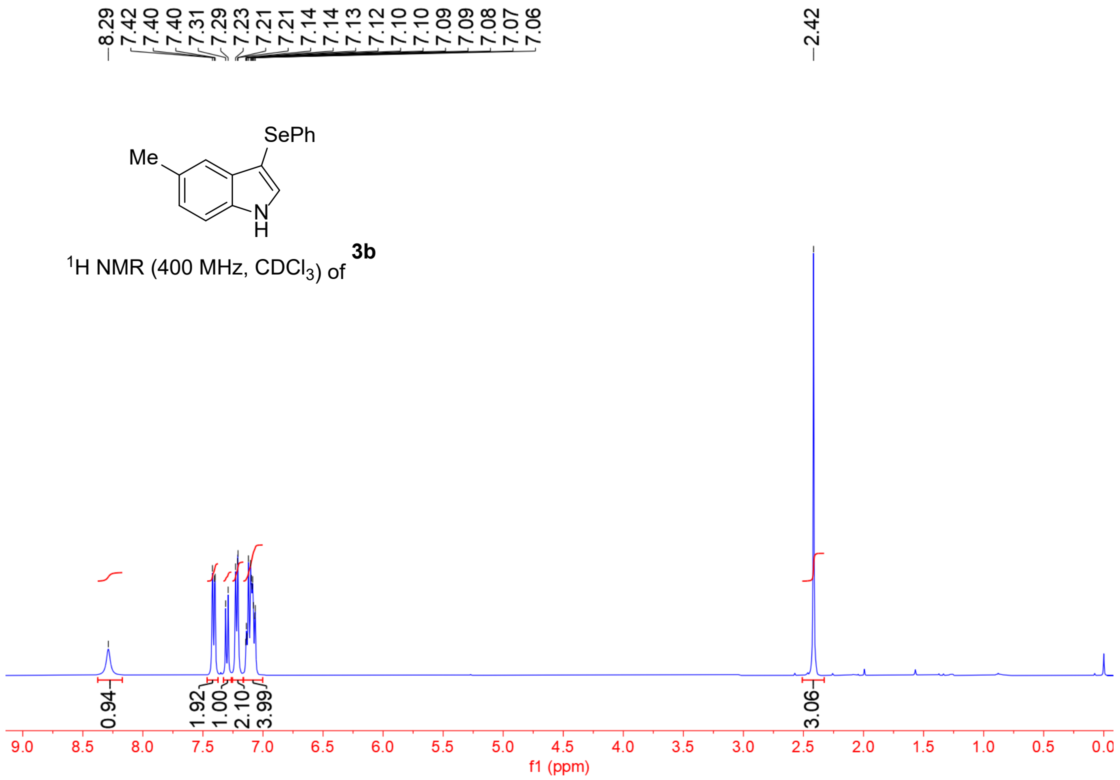

Figure S3: ^{1}H NMR spectrum for compound **3b**

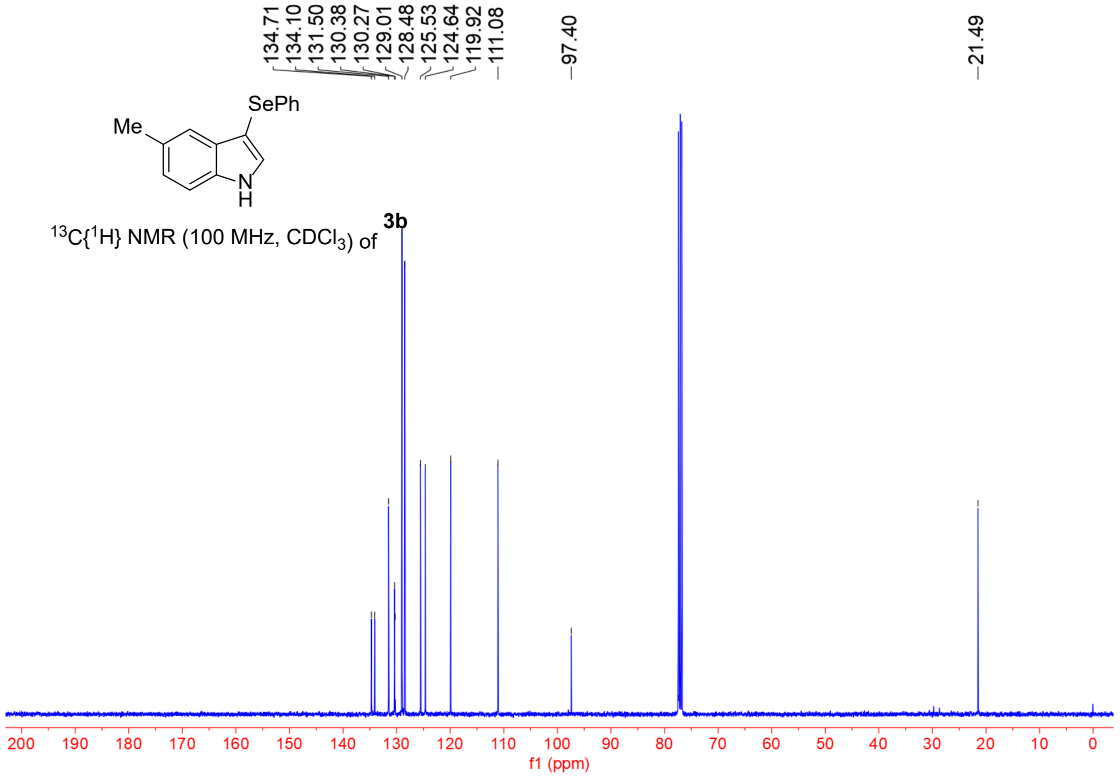

Figure S4: ^{13}C NMR spectrum for compound **3b**

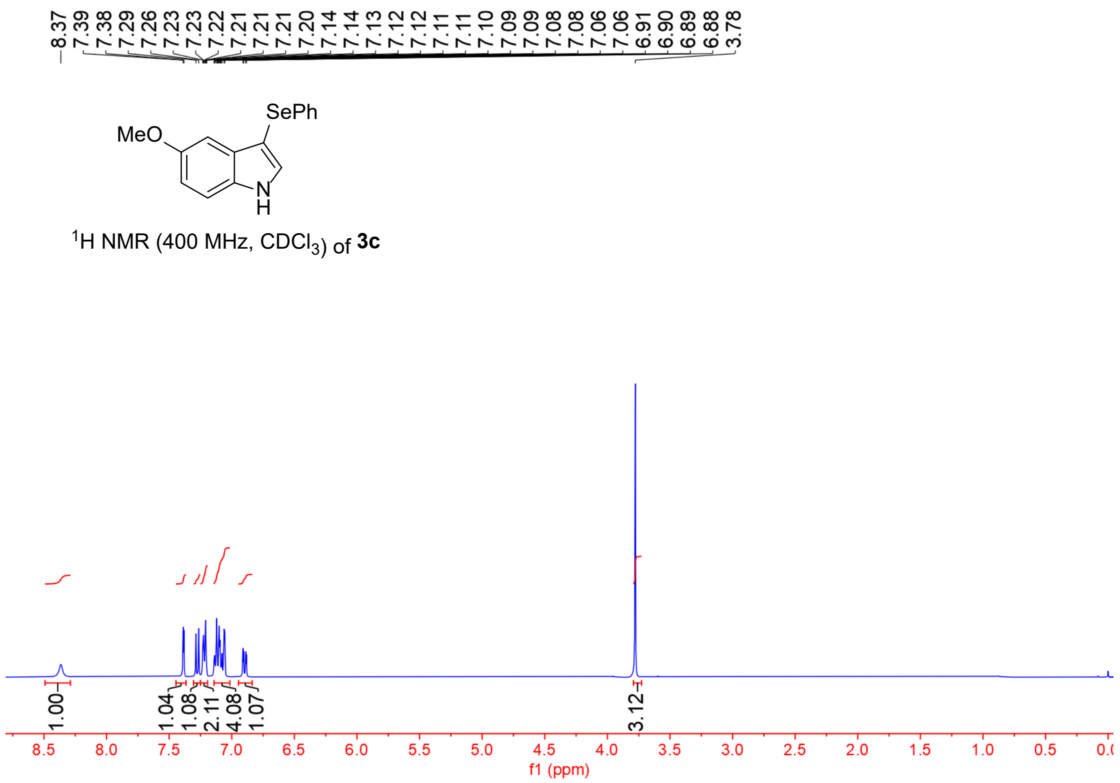

Figure S5: ^{1}H NMR spectrum for compound **3c**

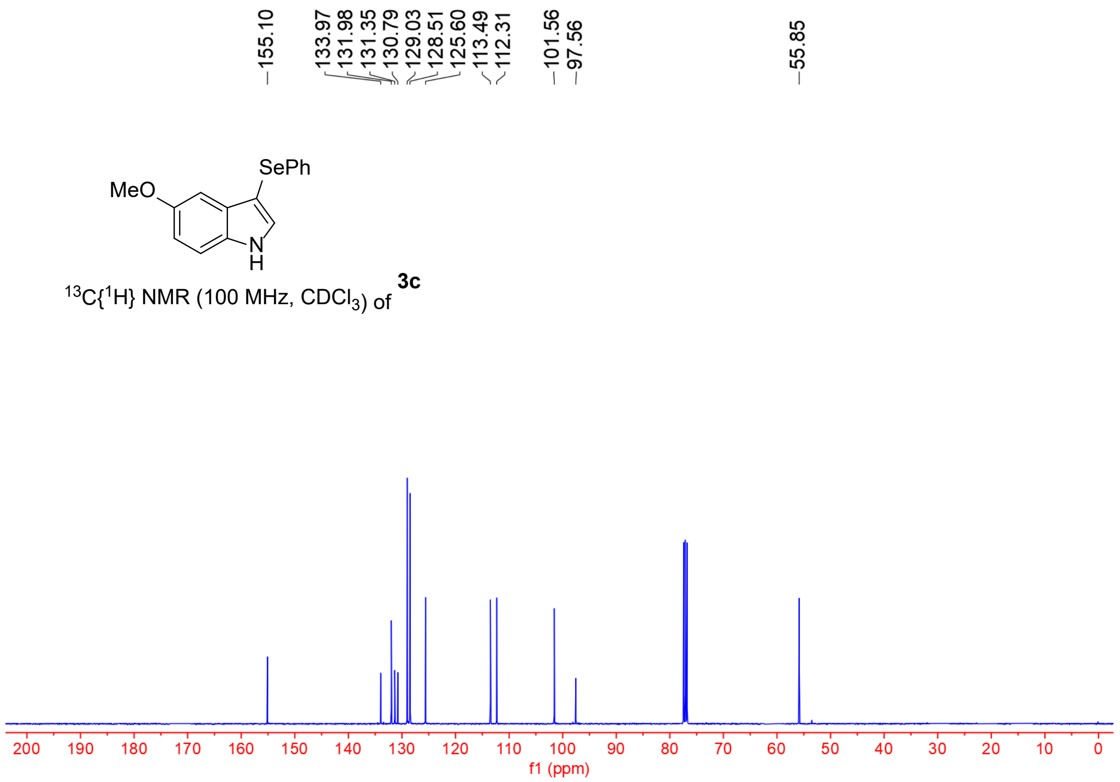

Figure S6: ^{13}C NMR spectrum for compound **3c**

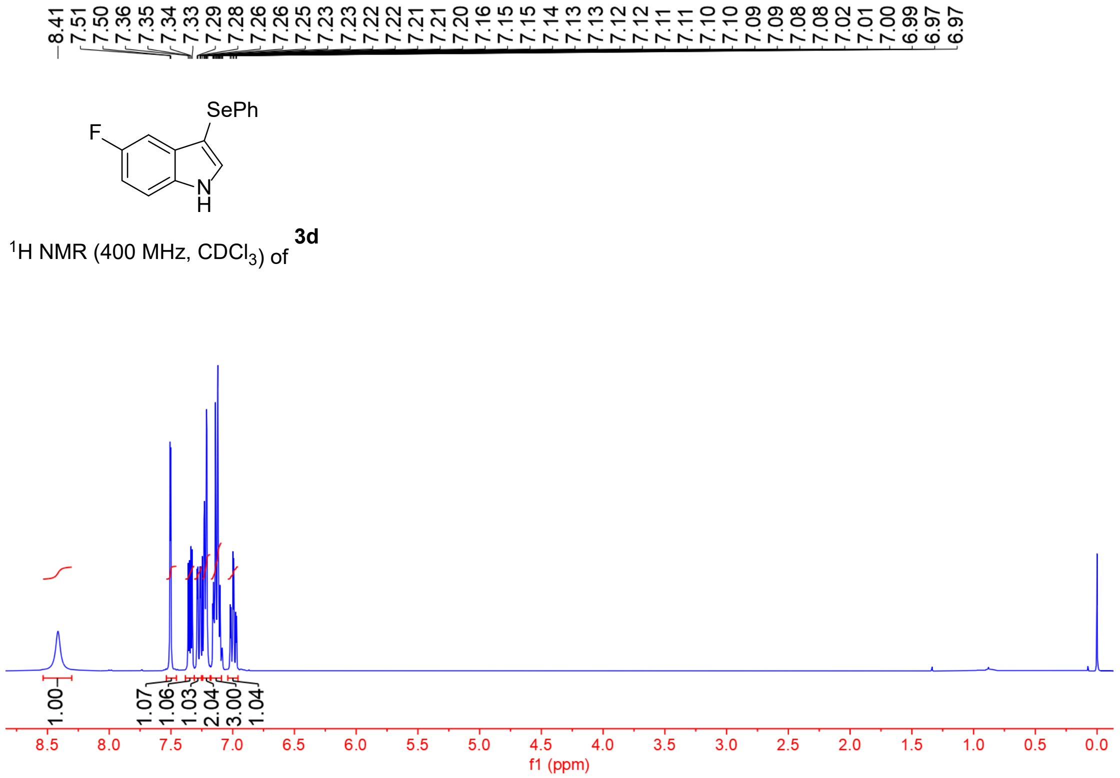

Figure S7: ^{1}H NMR spectrum for compound **3d**

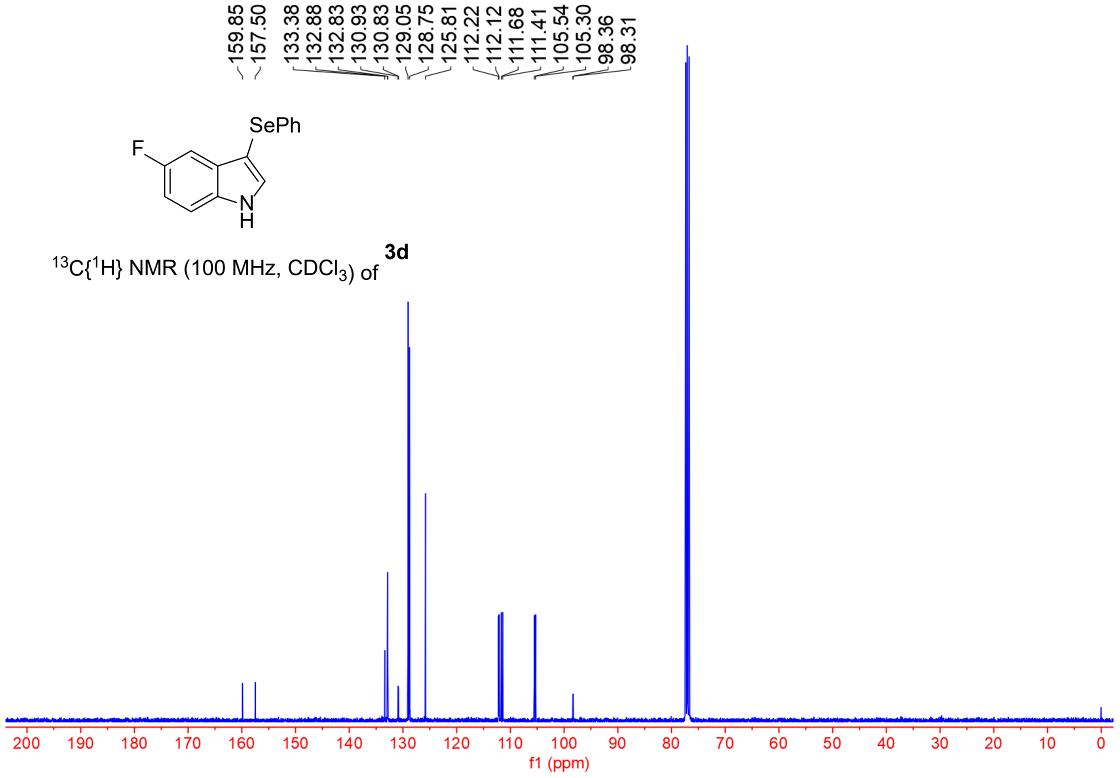

Figure S8: ^{13}C NMR spectrum for compound **3d**

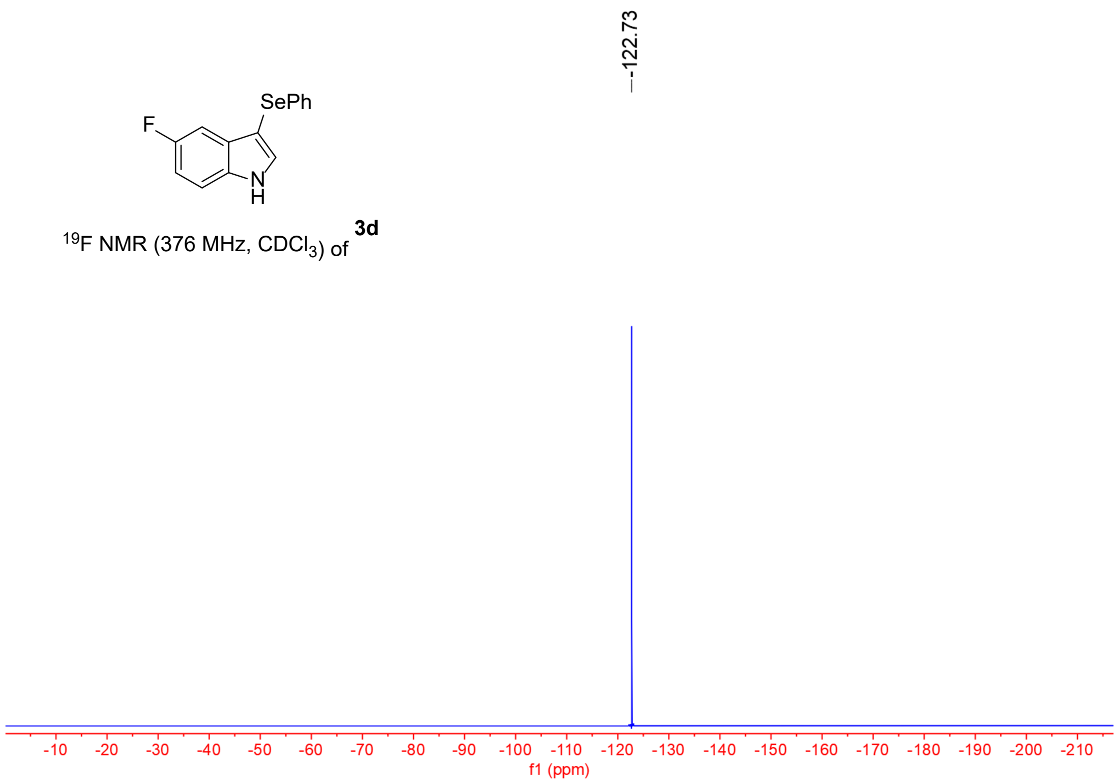

Figure S9: ^{19}F NMR spectrum for compound **3d**

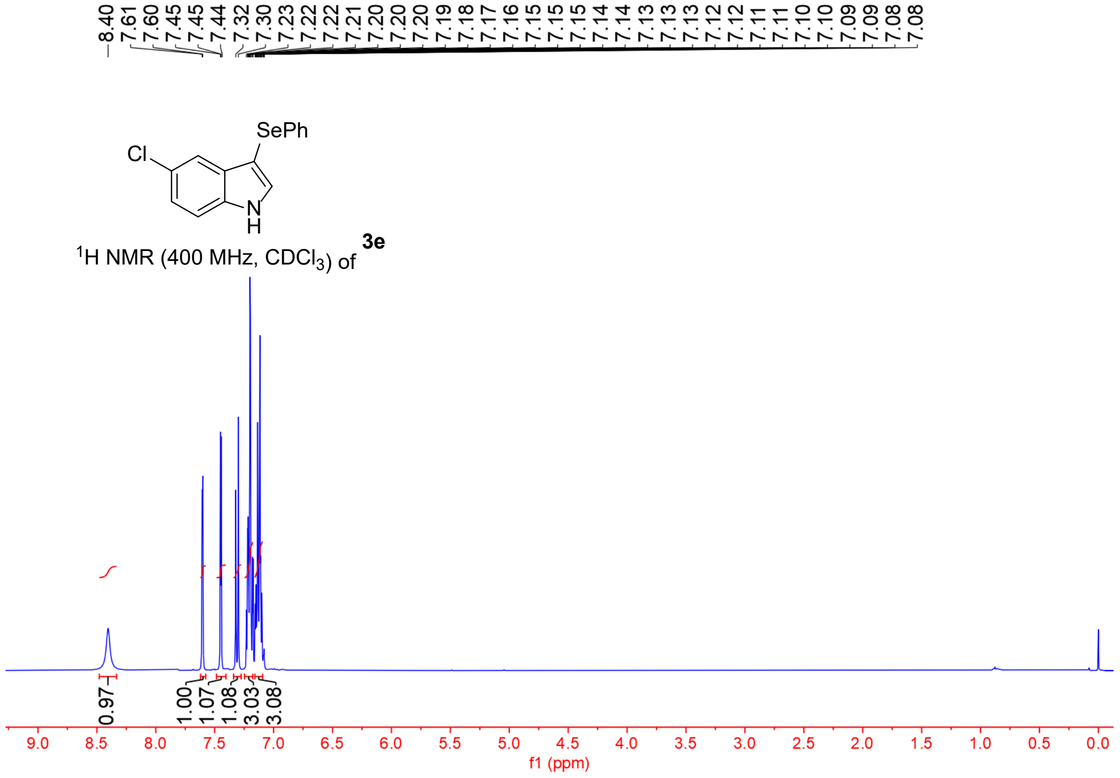

Figure S10: ^{1}H NMR spectrum for compound **3e**

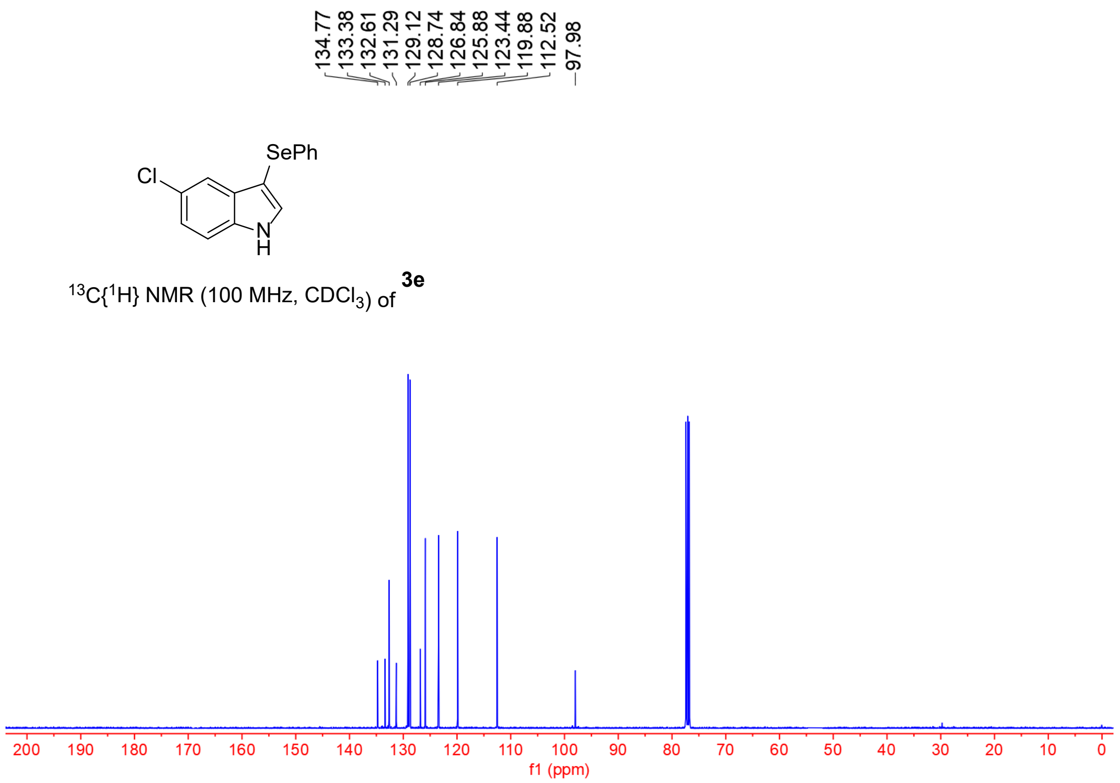

Figure S11: ^{13}C NMR spectrum for compound **3e**

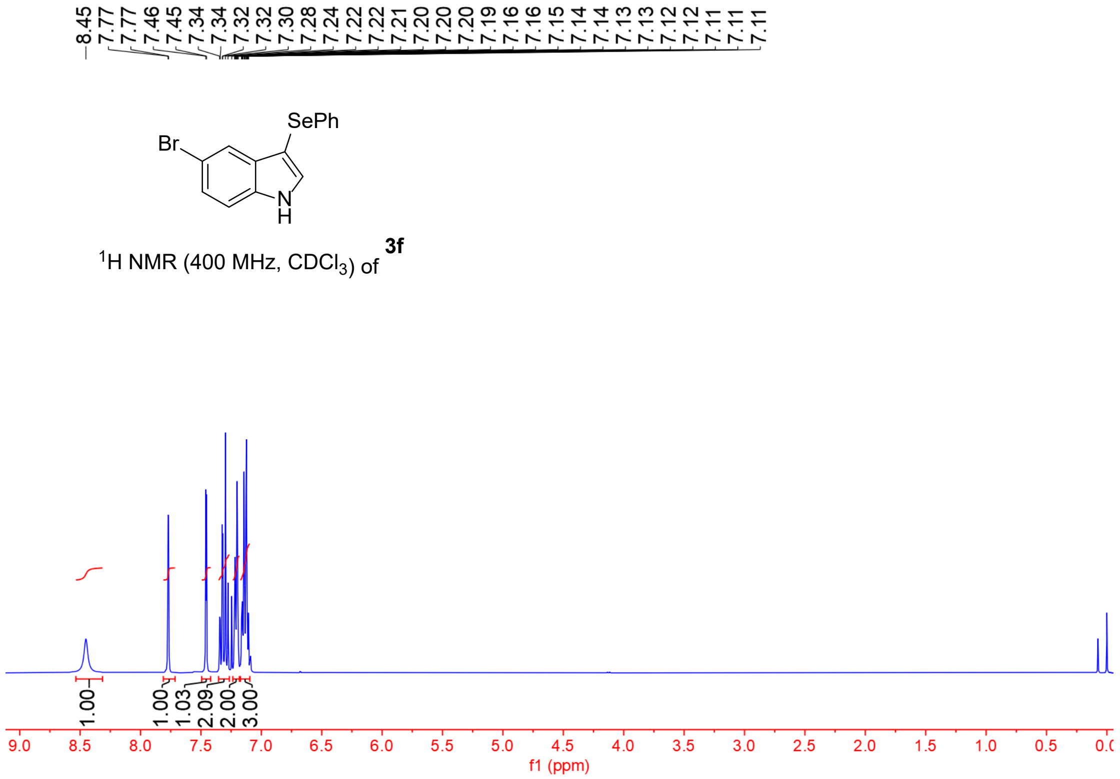

Figure S12: ^{1}H NMR spectrum for compound **3f**

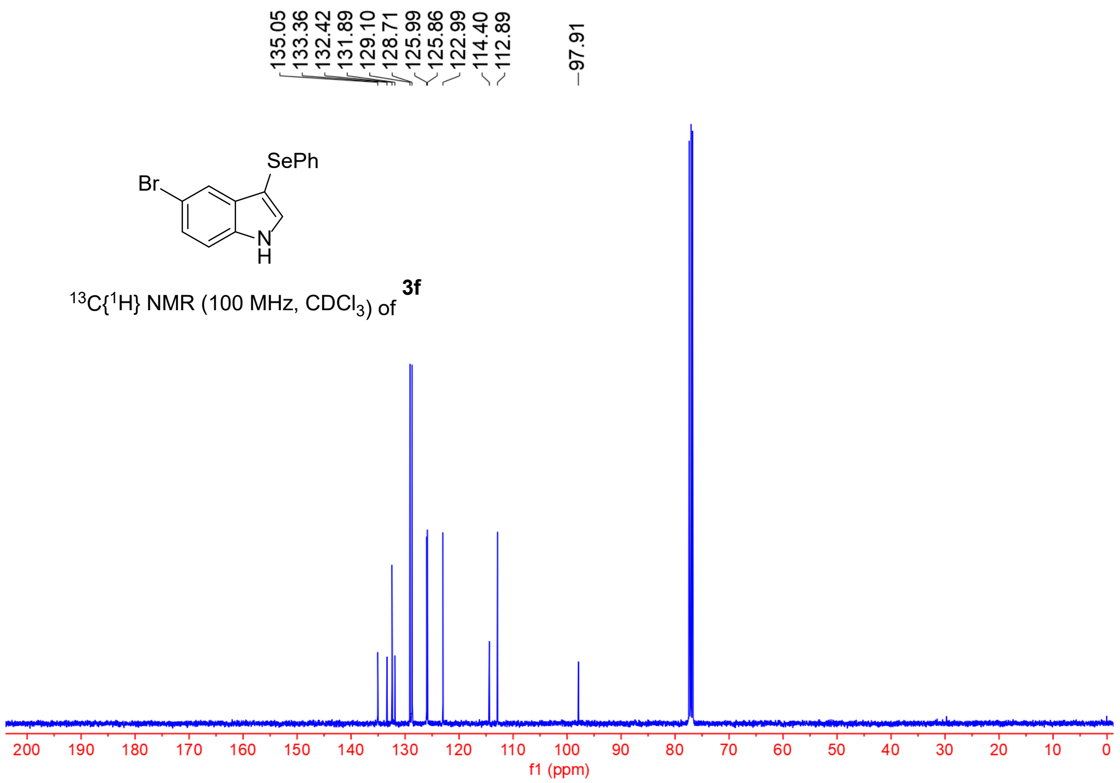

Figure S13: ^{13}C NMR spectrum for compound **3f**

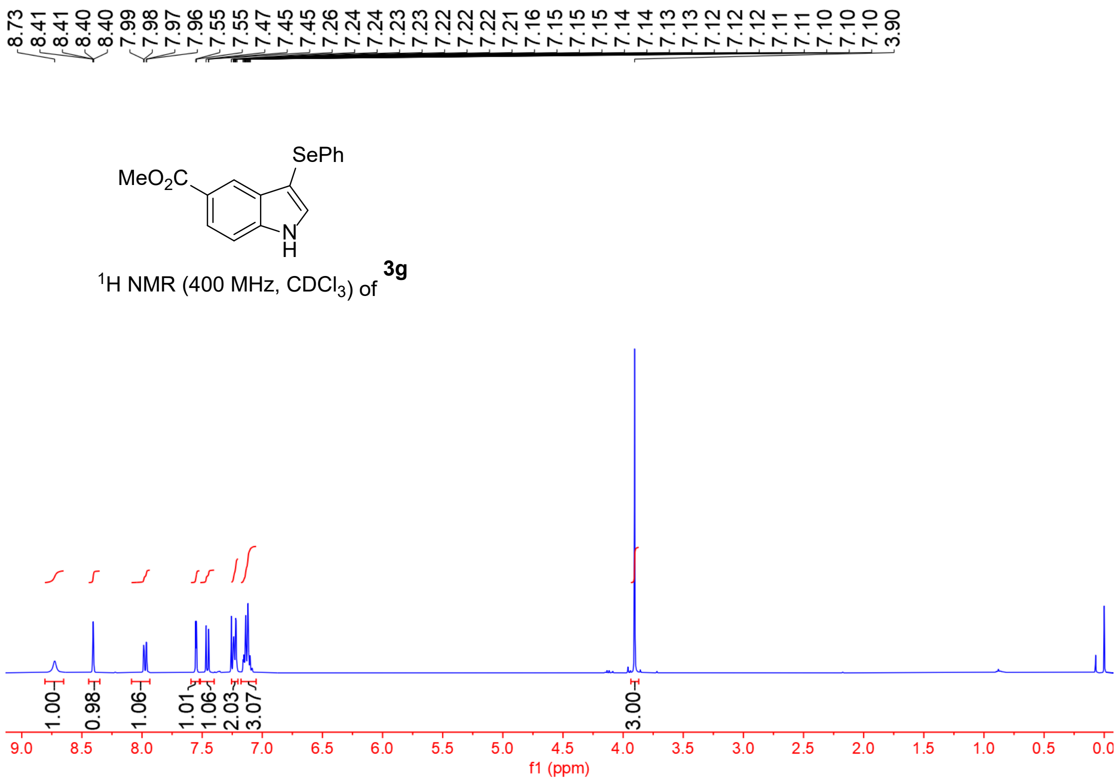

Figure S14: ^{1}H NMR spectrum for compound **3g**

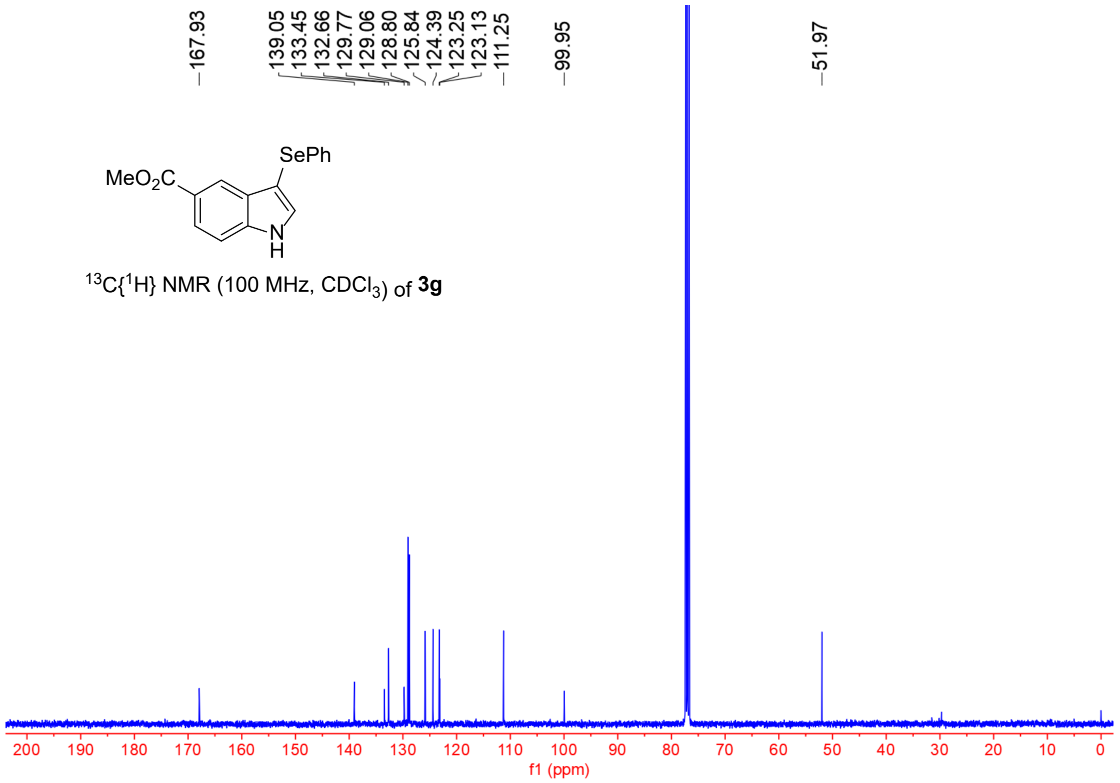

Figure S15: ^{13}C NMR spectrum for compound **3g**

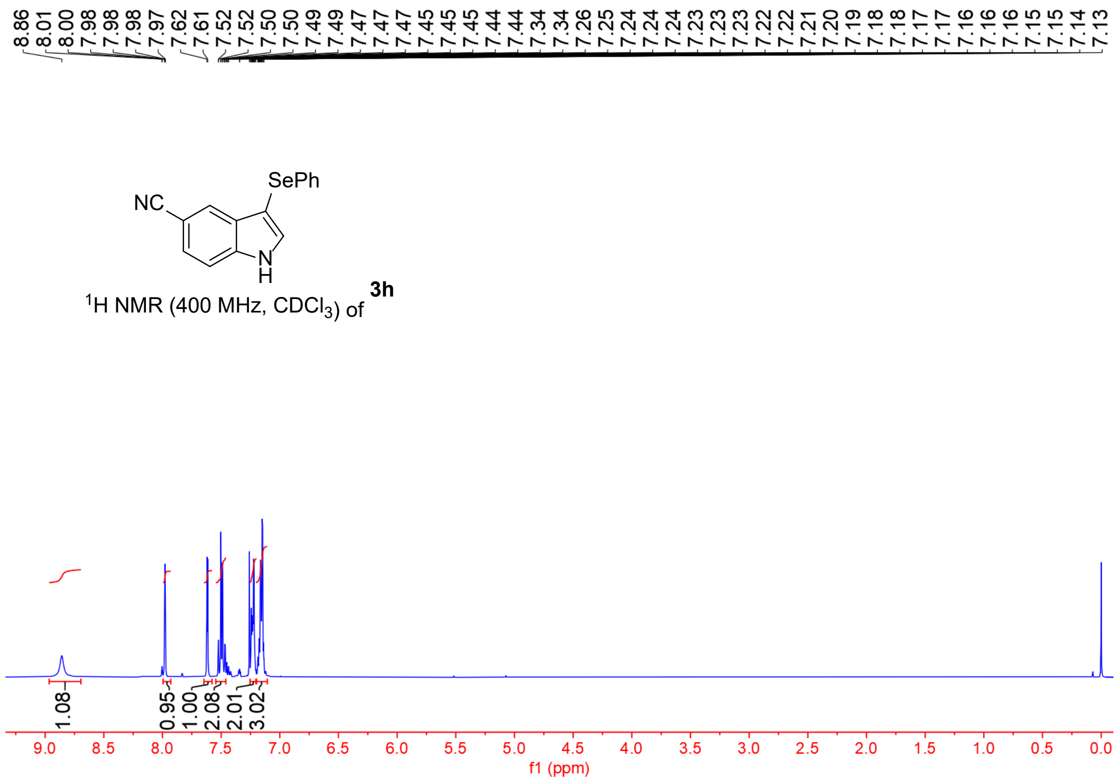

Figure S16: ^{1}H NMR spectrum for compound **3h**

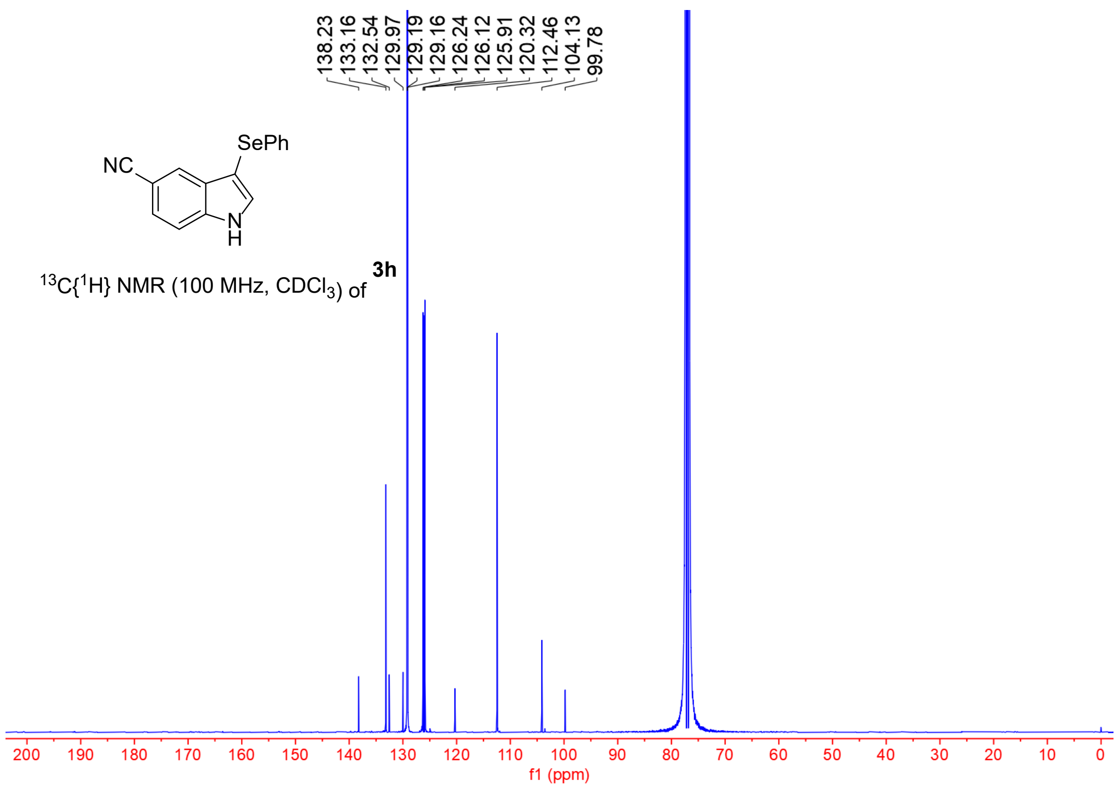

Figure S17: ^{13}C NMR spectrum for compound **3h**

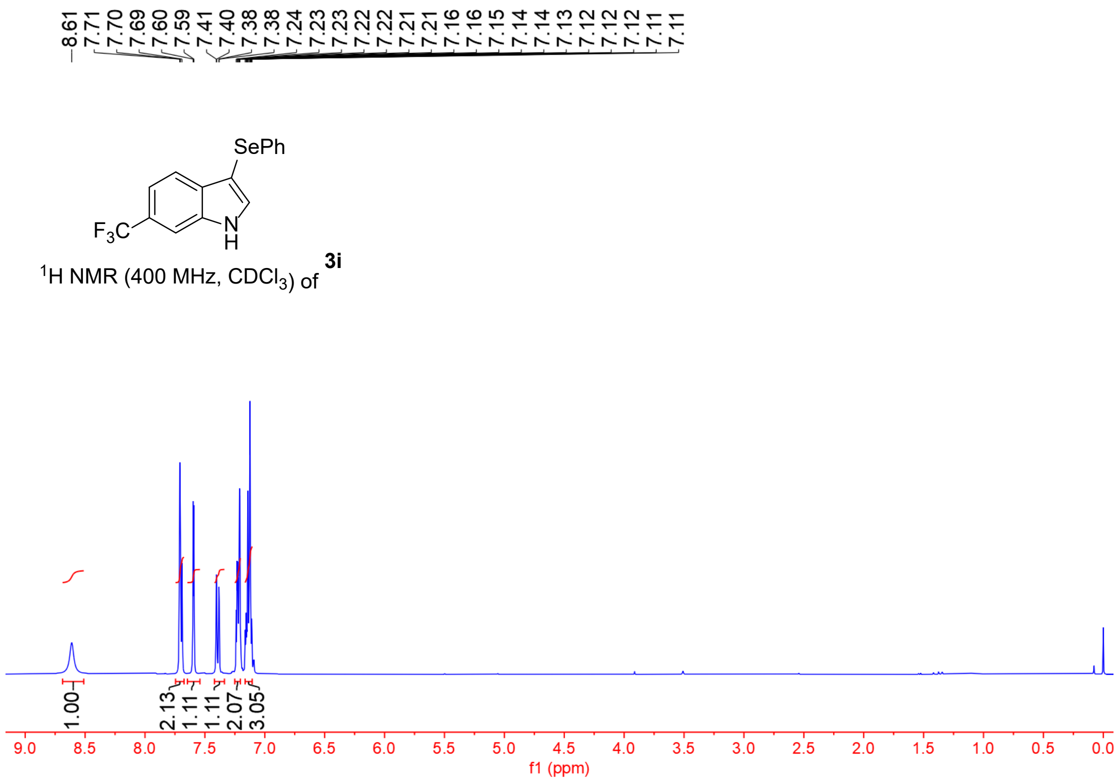

Figure S18: ^{1}H NMR spectrum for compound **3i**

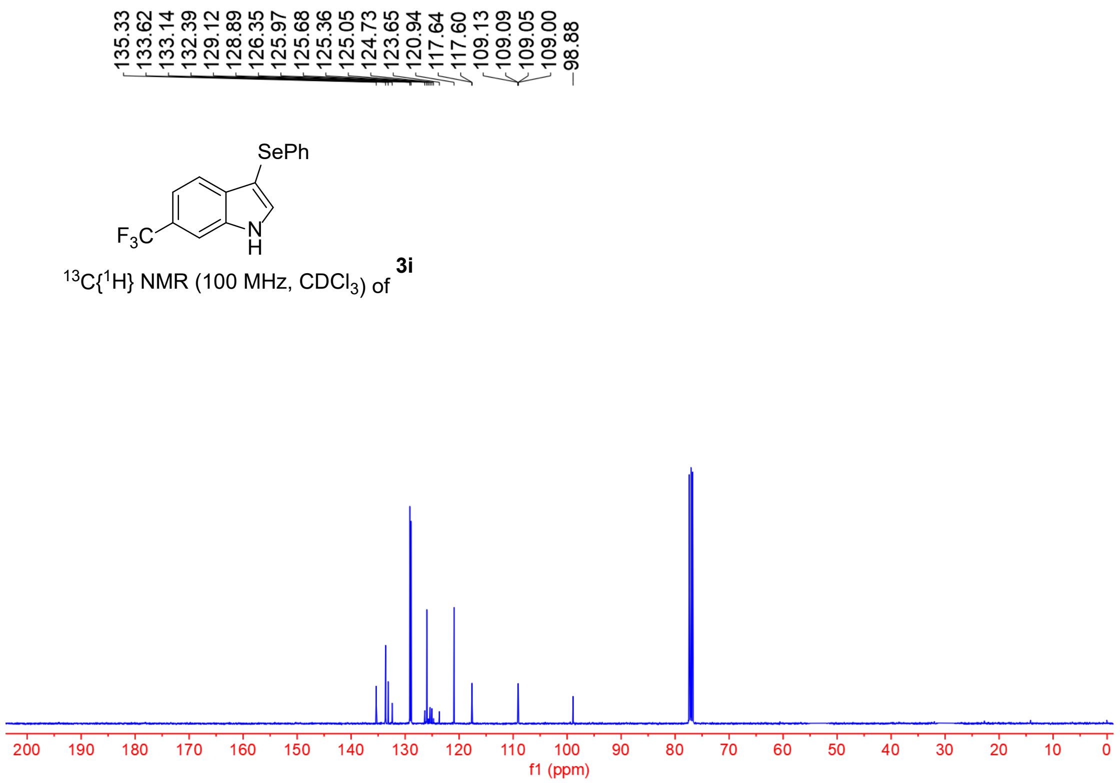

Figure S19: ^{13}C NMR spectrum for compound **3i**

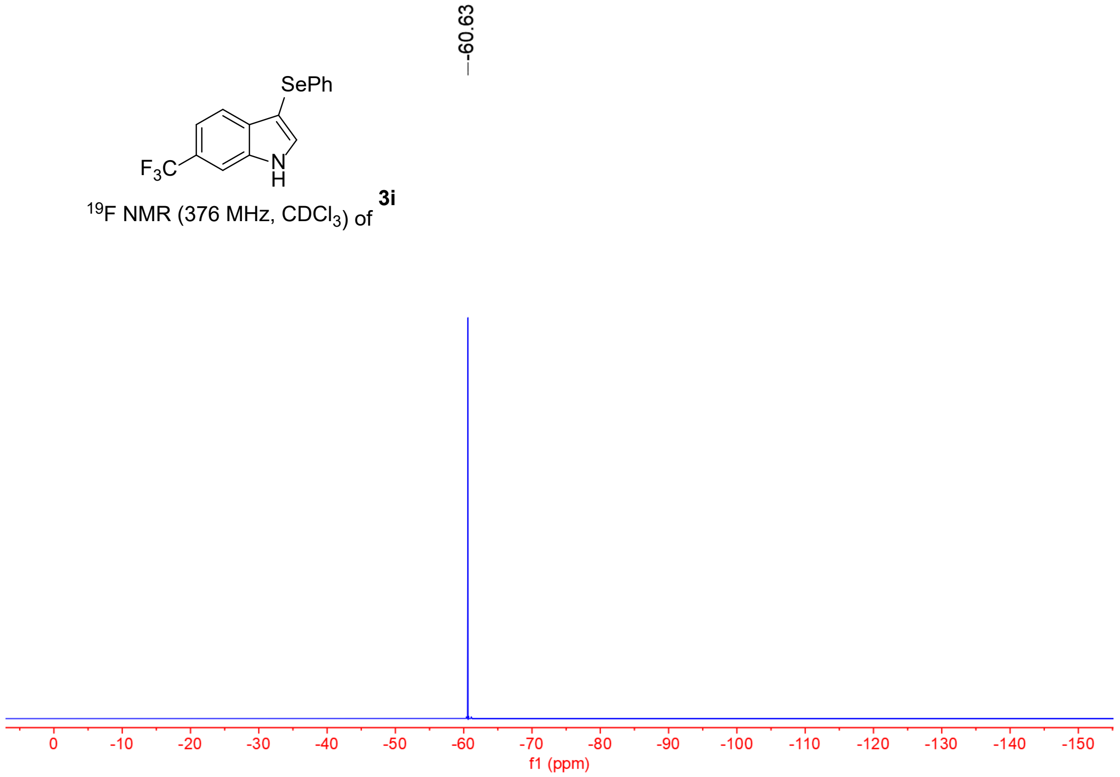

Figure S20: ^{19}F NMR spectrum for compound **3i**

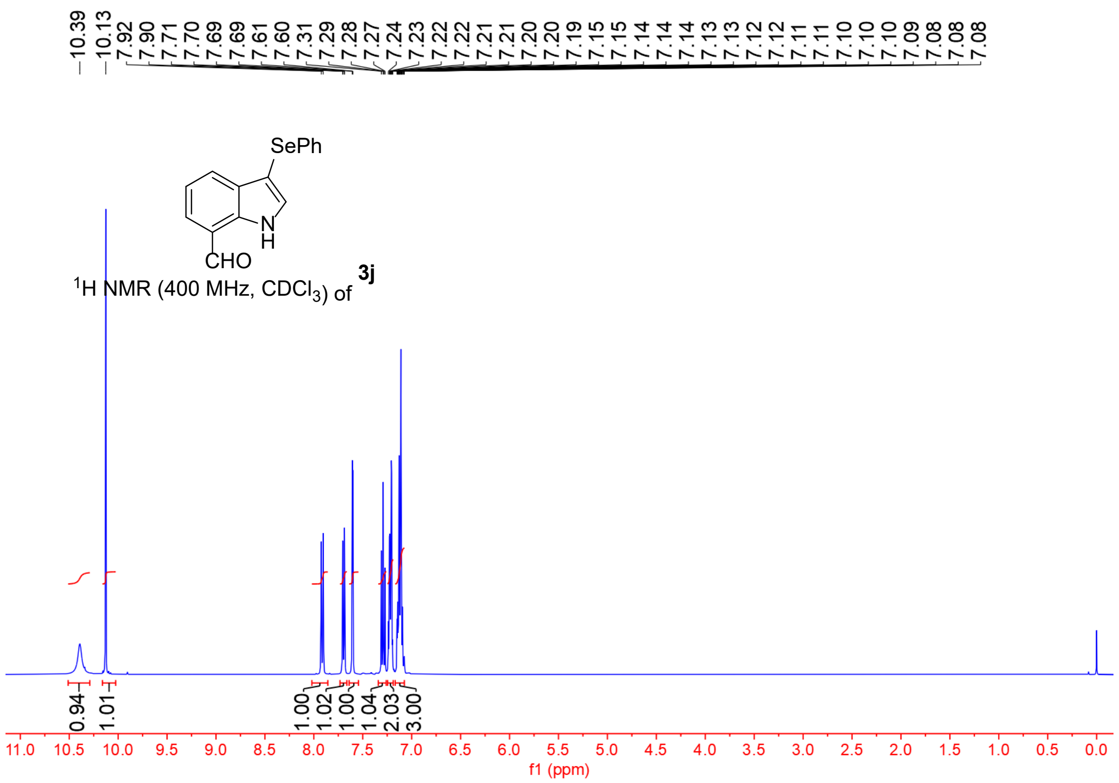

Figure S21: ^{1}H NMR spectrum for compound **3j**

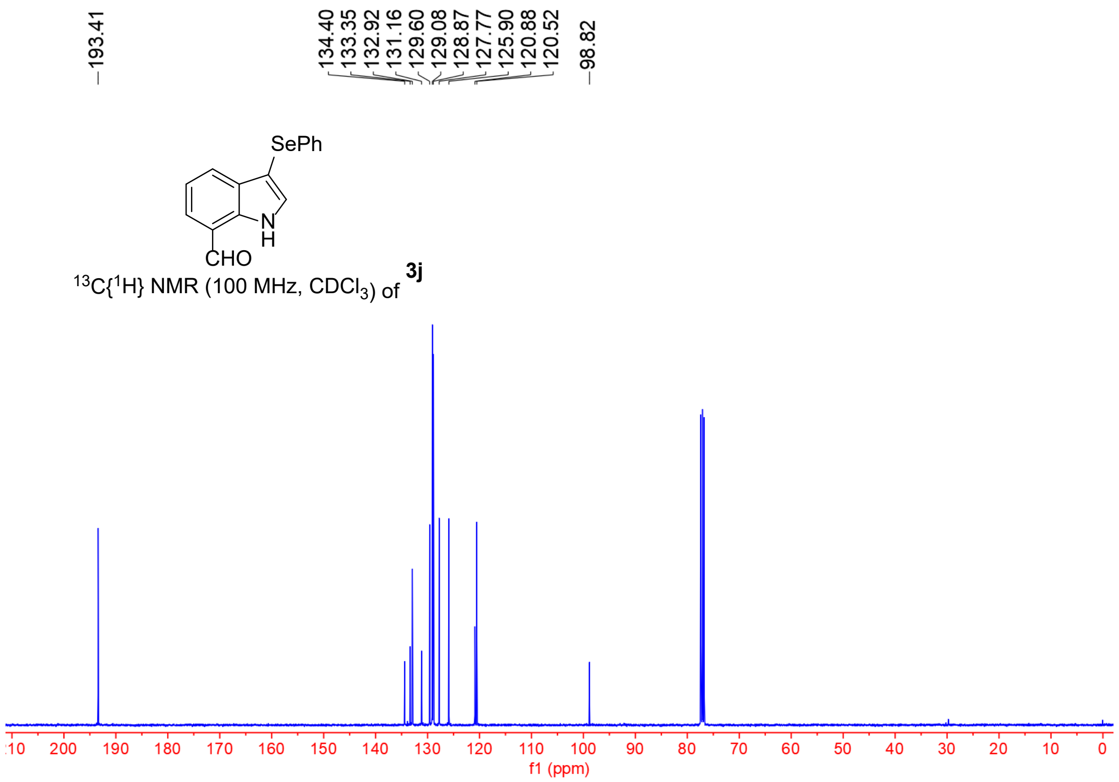

Figure S22: ^{13}C NMR spectrum for compound **3j**

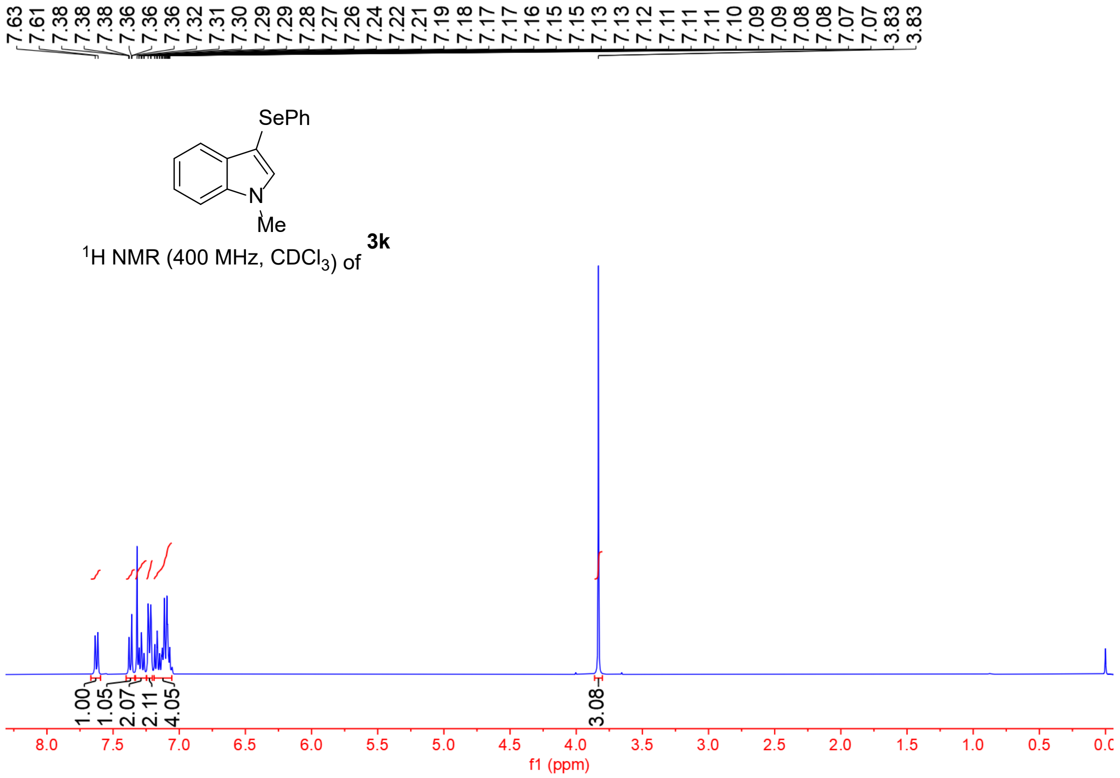

Figure S23: ^{1}H NMR spectrum for compound **3k**

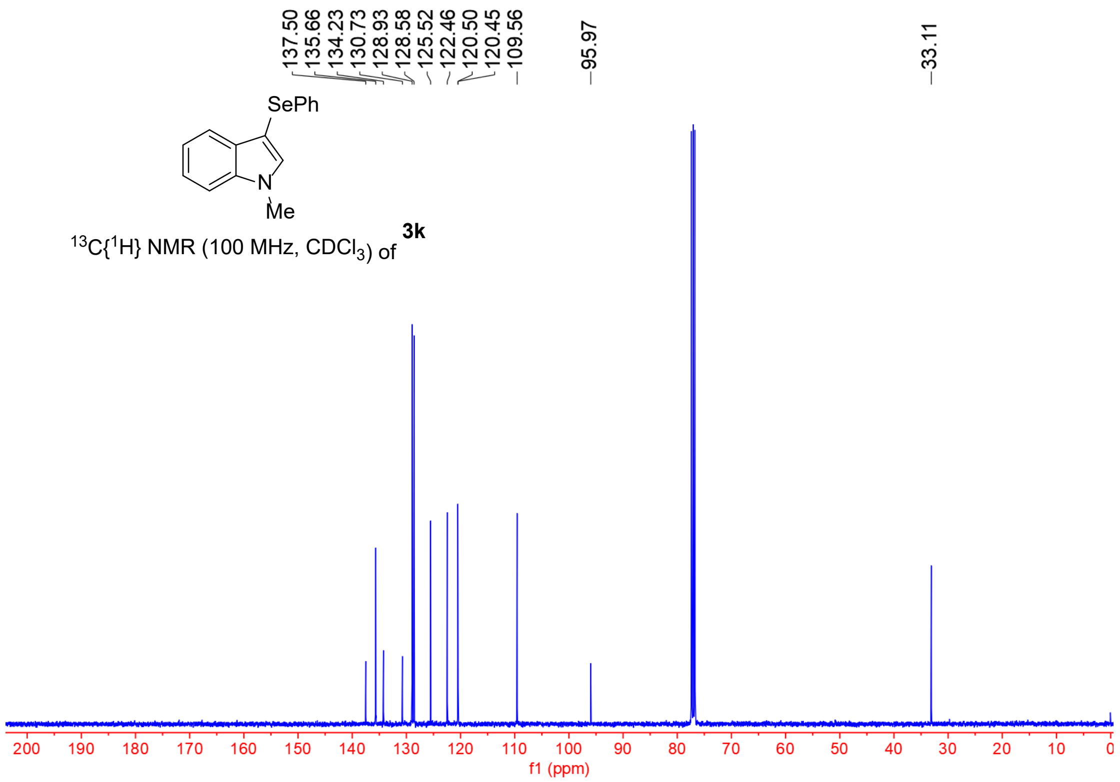

Figure S24: ^{13}C NMR spectrum for compound **3k**

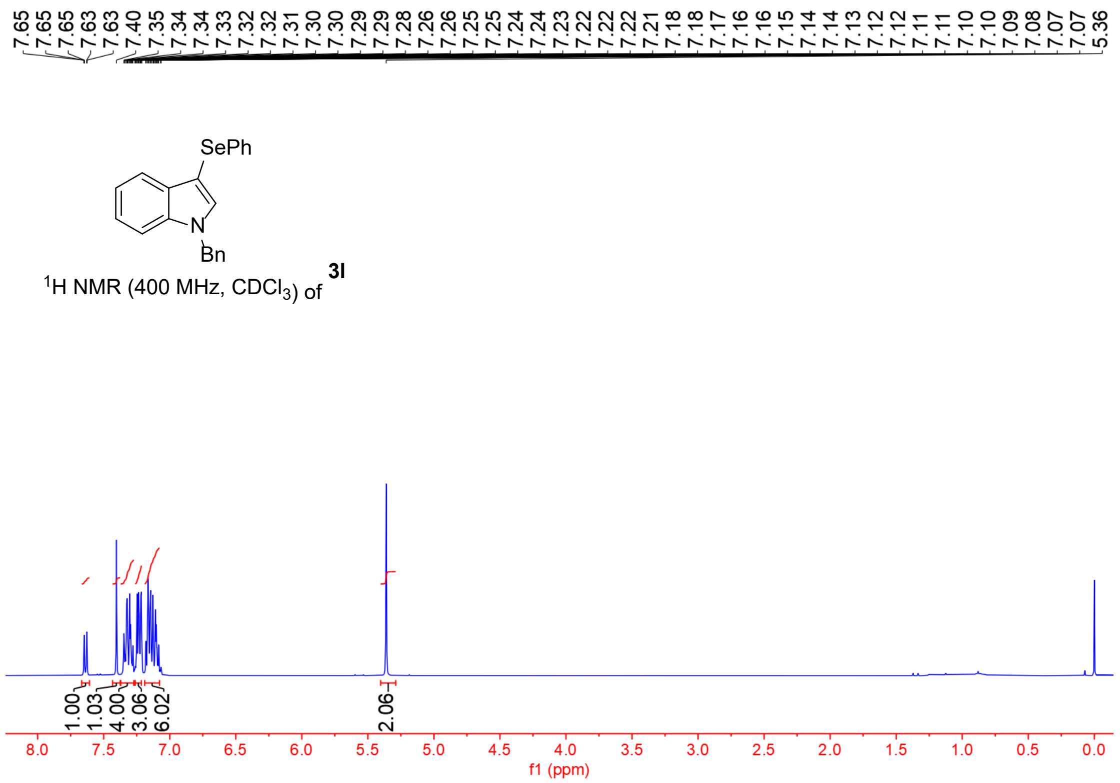

Figure S25: ^{1}H NMR spectrum for compound **3l**

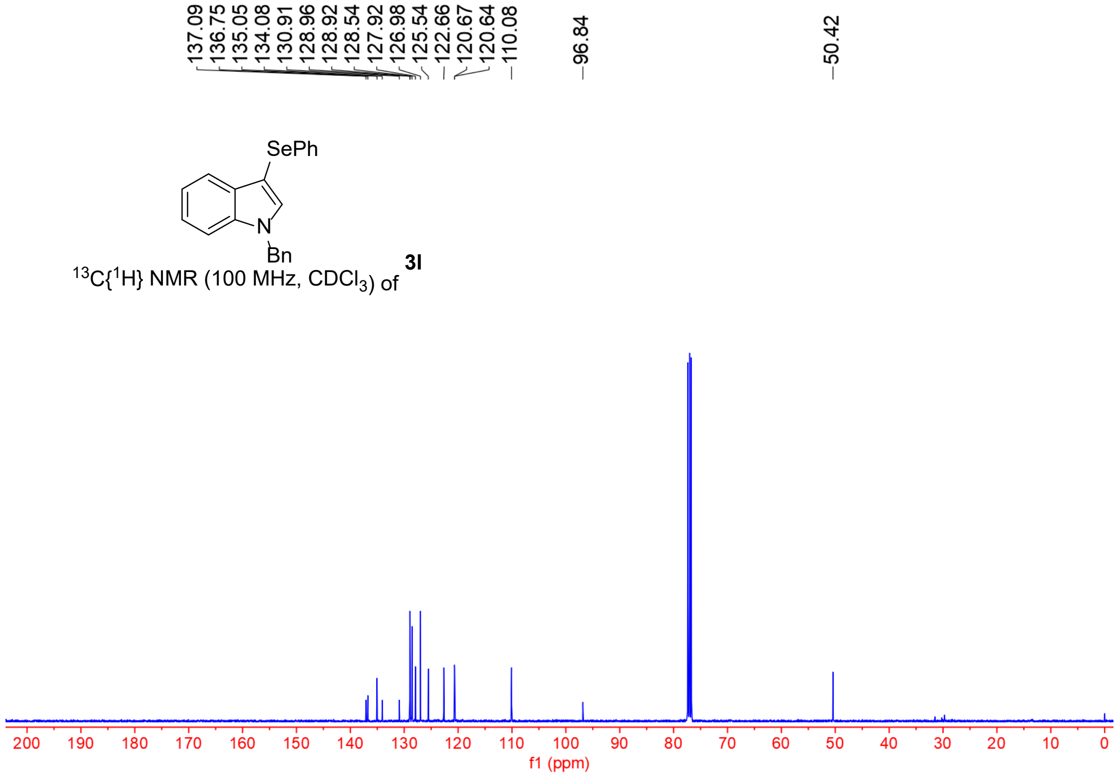

Figure S26: ^{13}C NMR spectrum for compound **3l**

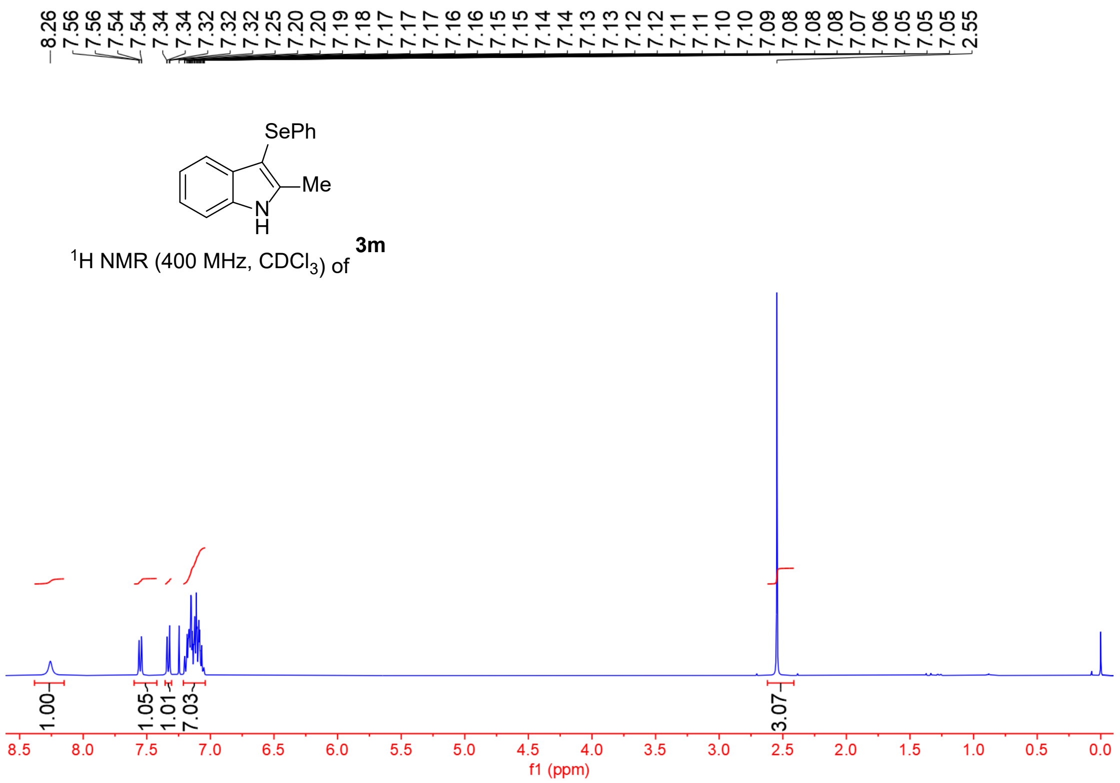

Figure S27: ^{1}H NMR spectrum for compound **3m**

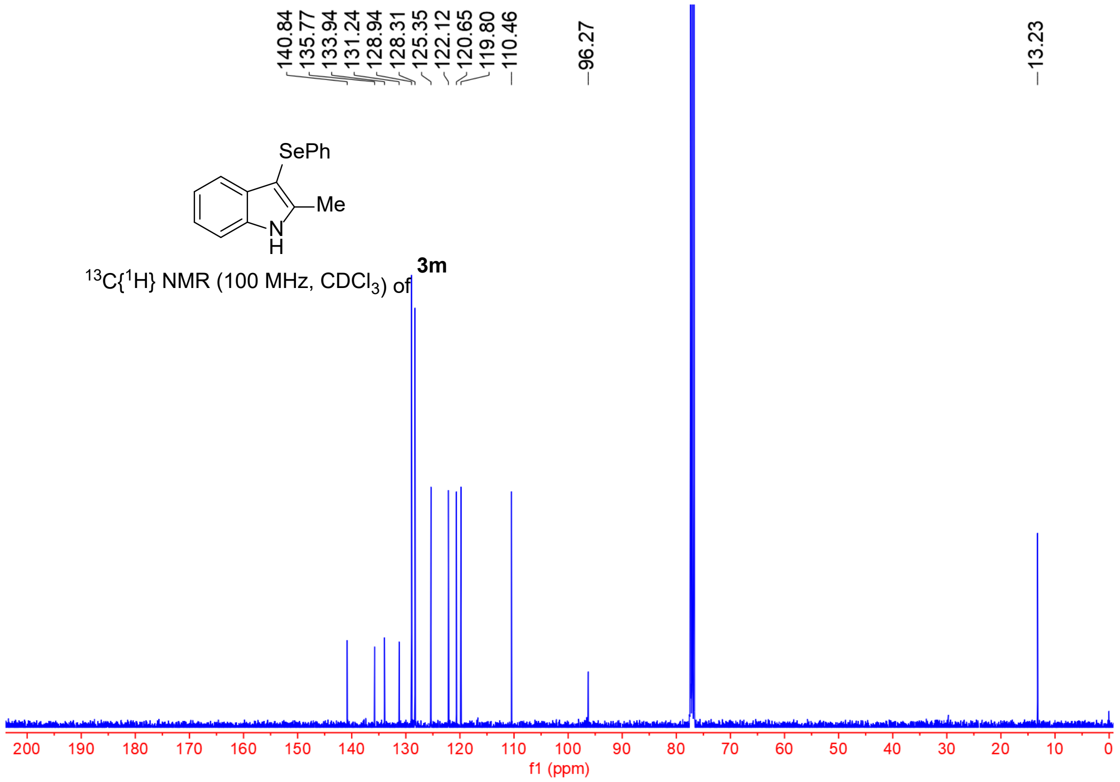

Figure S28: ^{13}C NMR spectrum for compound **3m**

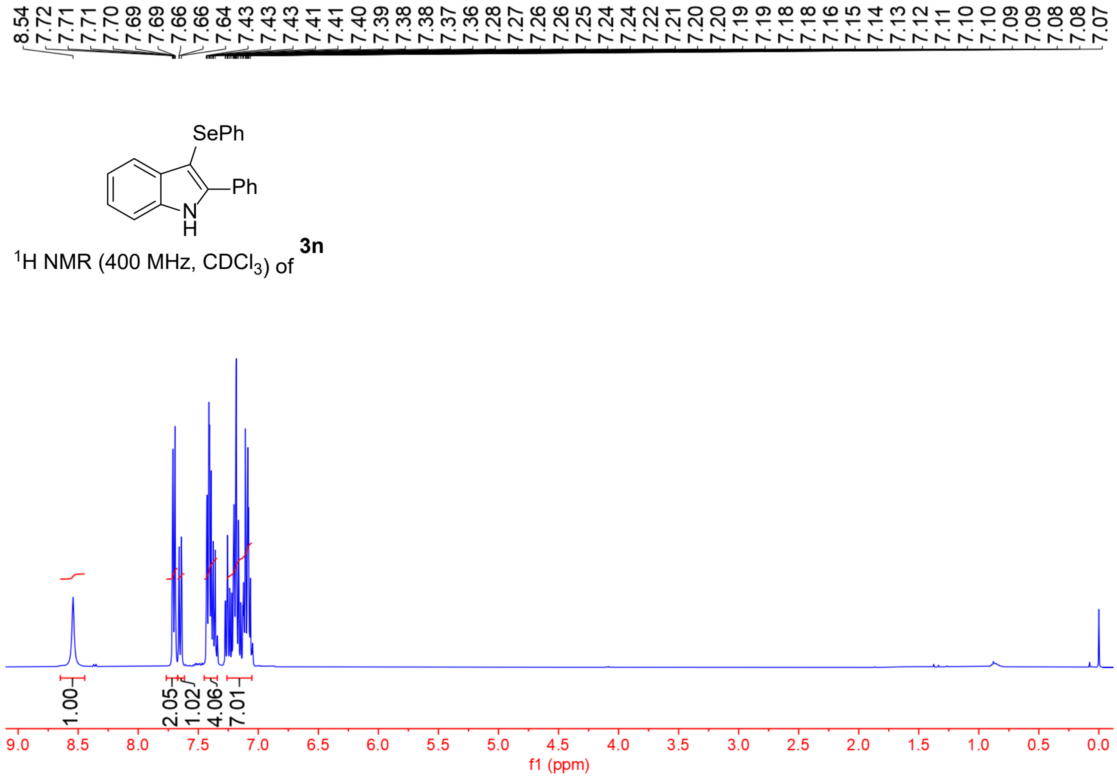

Figure S29: ^{1}H NMR spectrum for compound **3n**

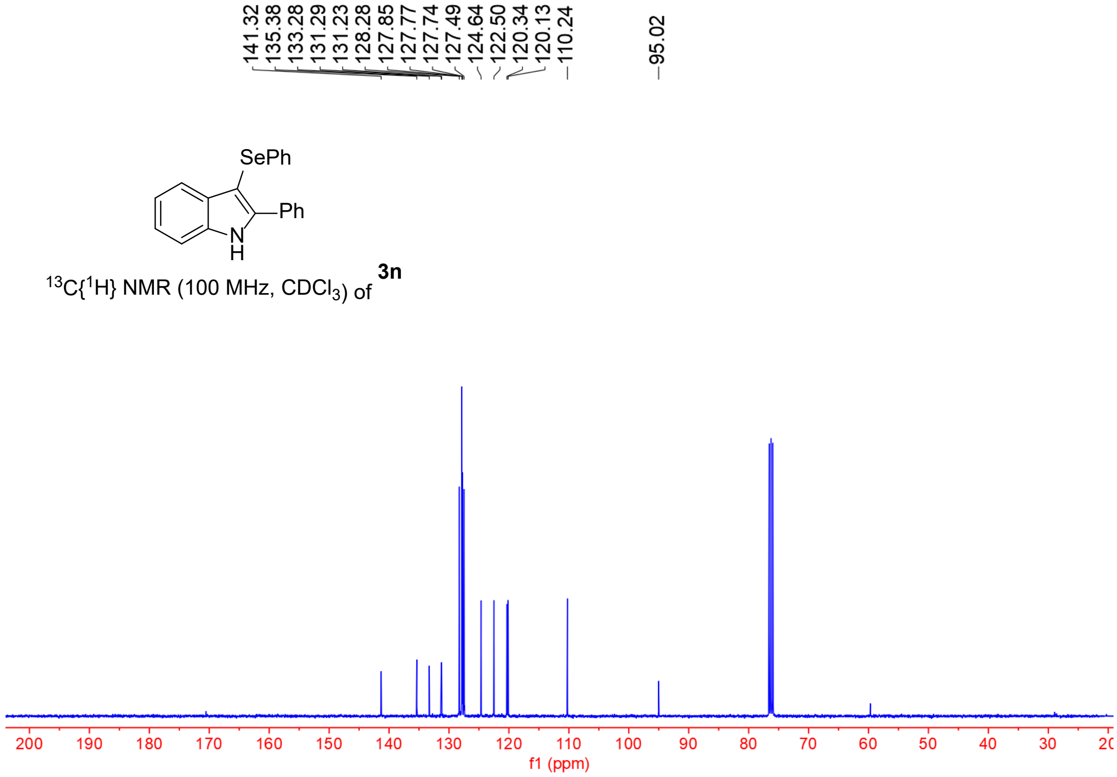

Figure S30: ^{13}C NMR spectrum for compound **3n**

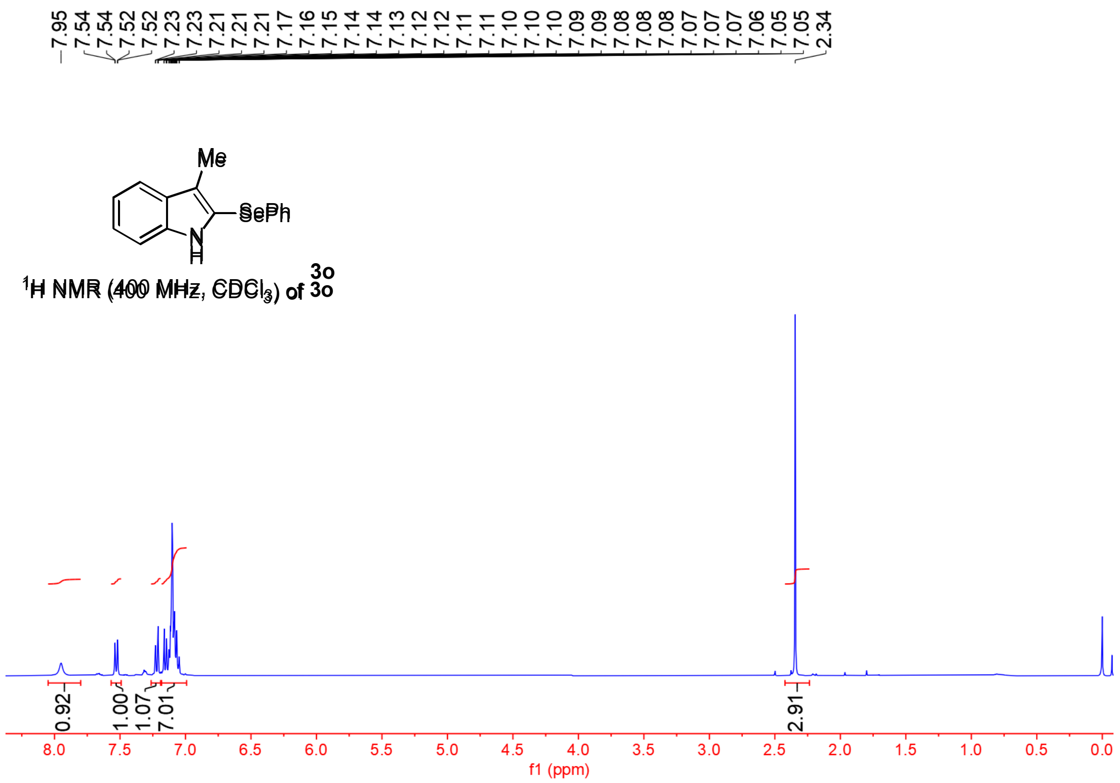

Figure S31: ^{1}H NMR spectrum for compound **3o**

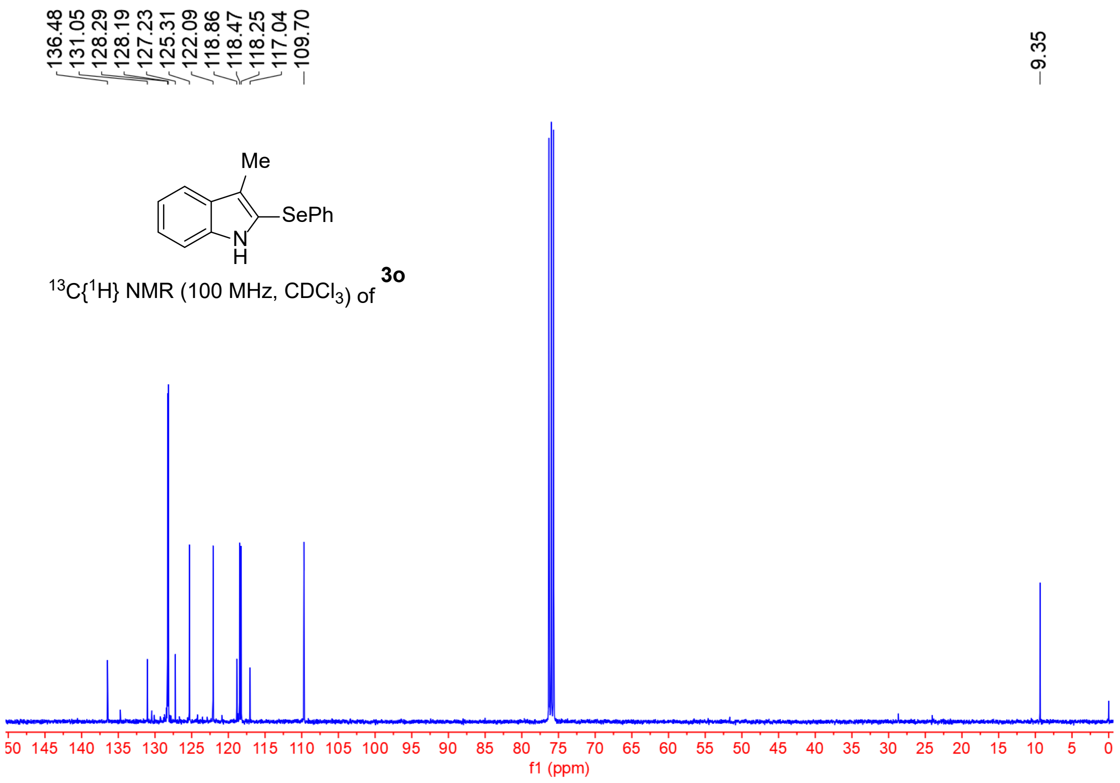

Figure S32: ^{13}C NMR spectrum for compound **3o**

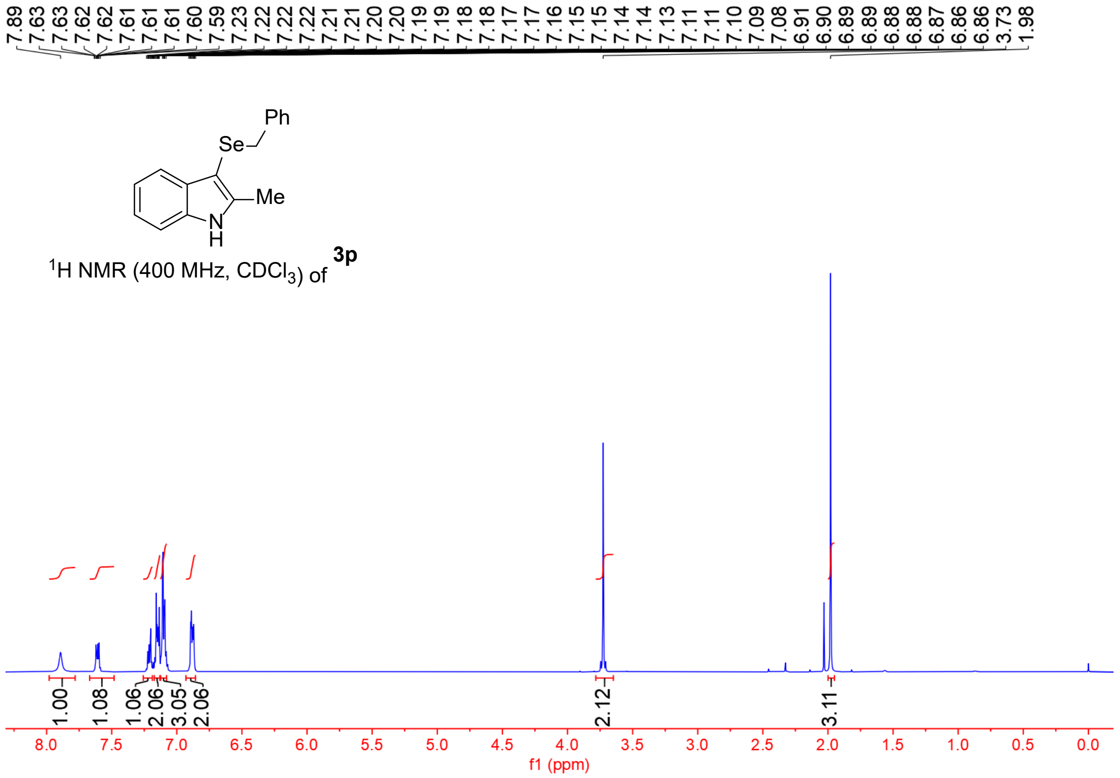

Figure S33: ^{1}H NMR spectrum for compound **3p**

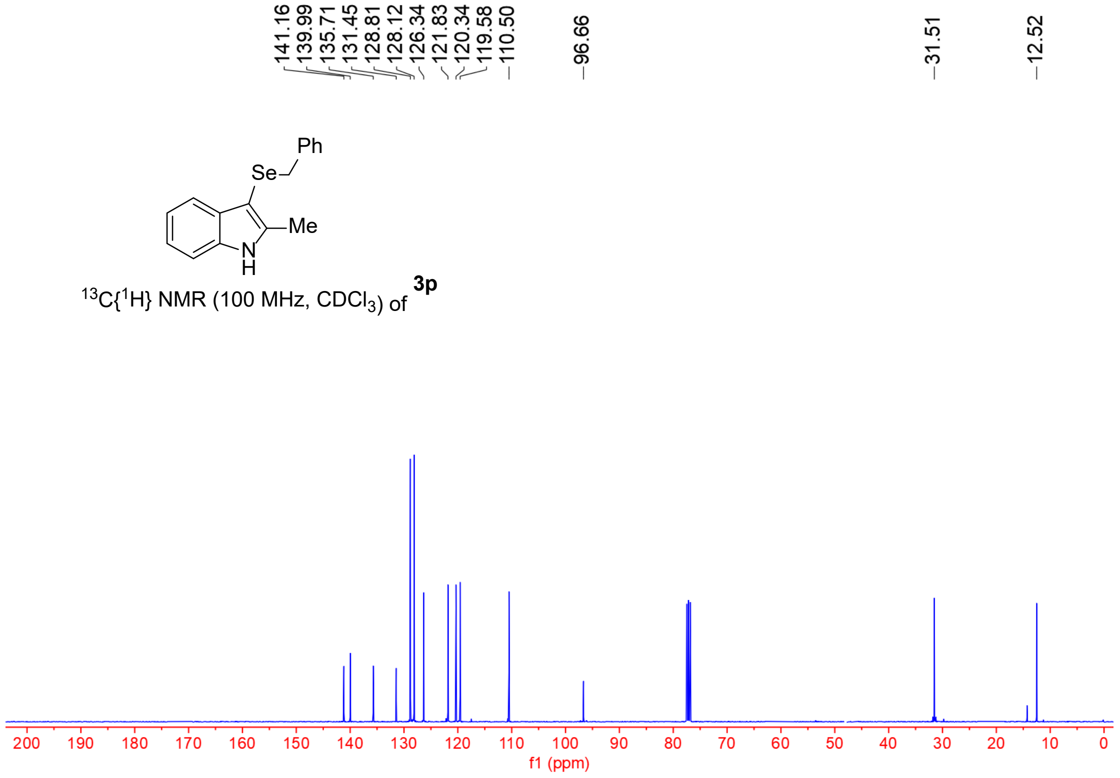

Figure S34: ^{13}C NMR spectrum for compound **3p**

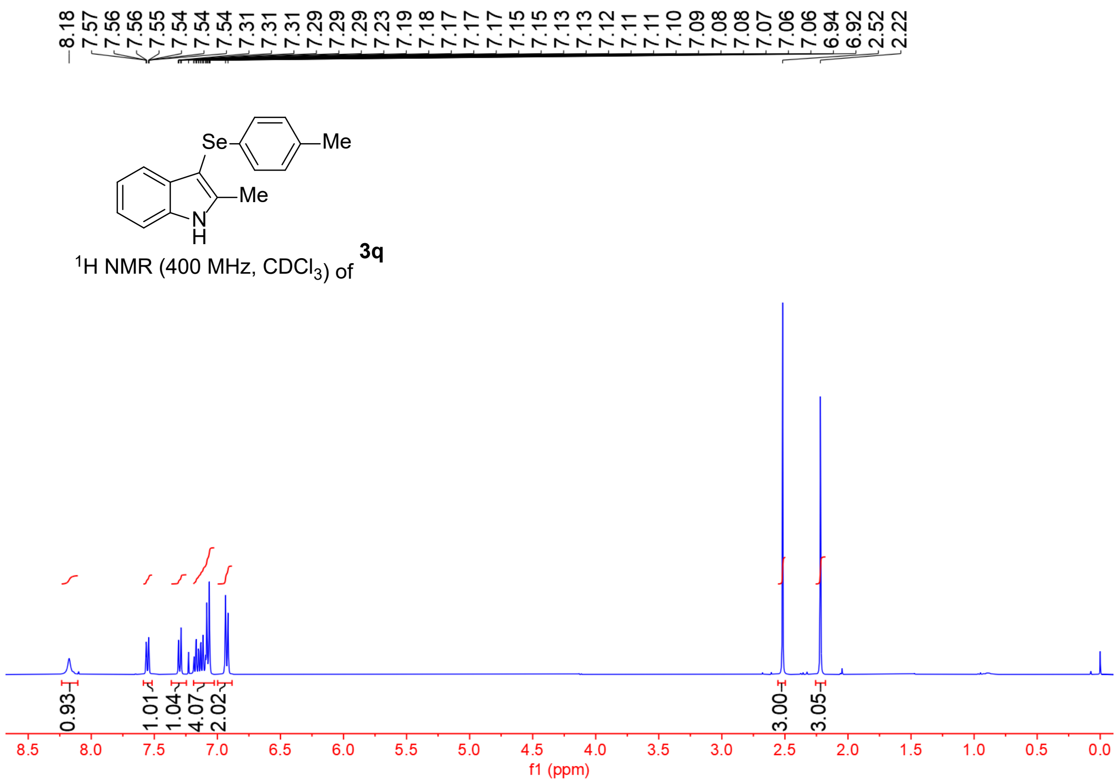

Figure S35: ^{1}H NMR spectrum for compound **3q**

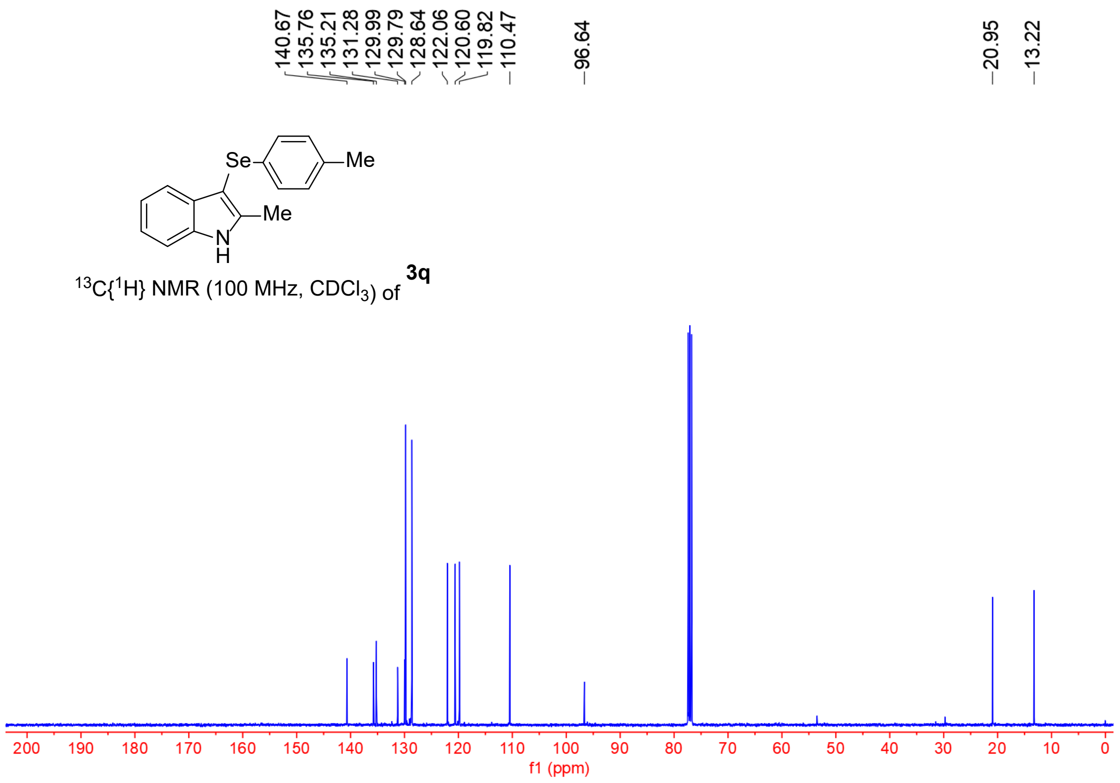

Figure S36: ^{13}C NMR spectrum for compound **3q**

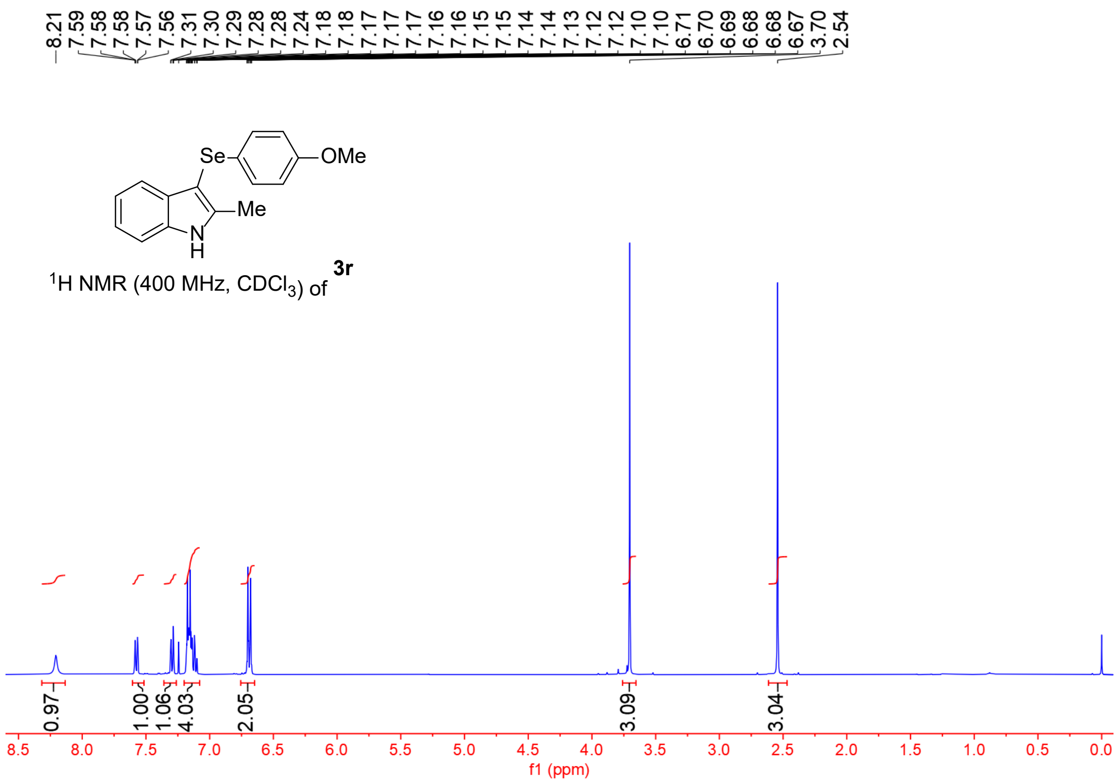

Figure S37: ^{1}H NMR spectrum for compound **3r**

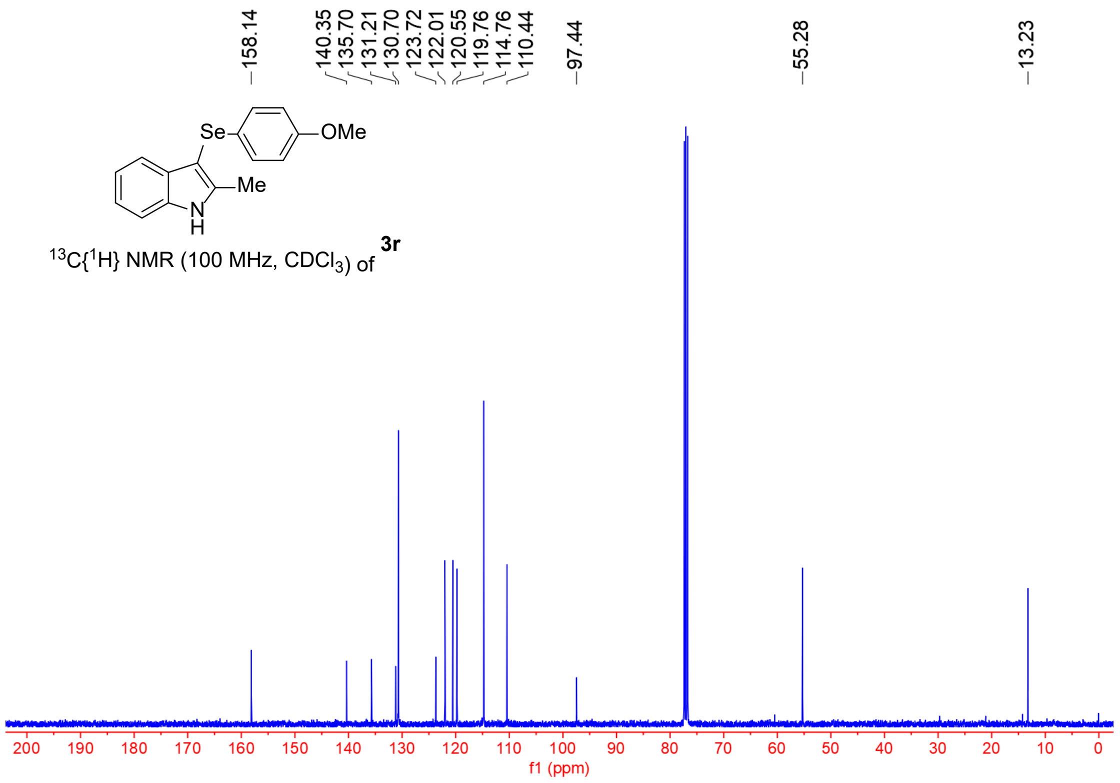

Figure S38: ^{13}C NMR spectrum for compound **3r**

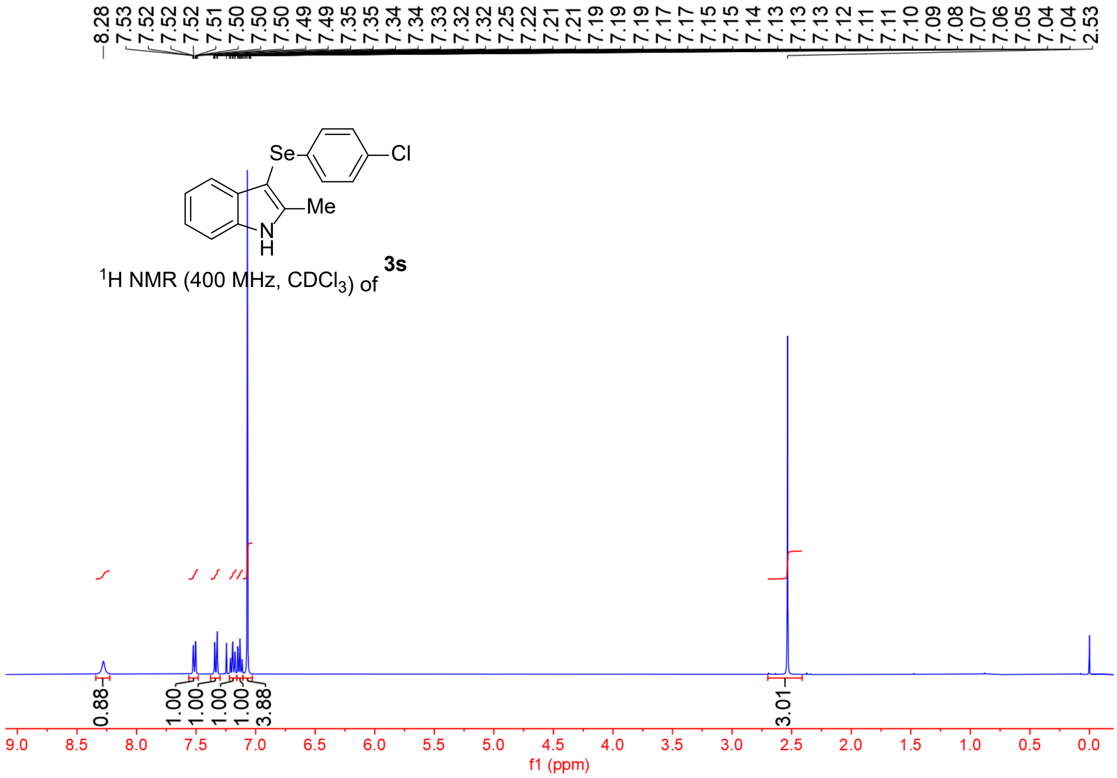

Figure S39: ^{1}H NMR spectrum for compound **3s**

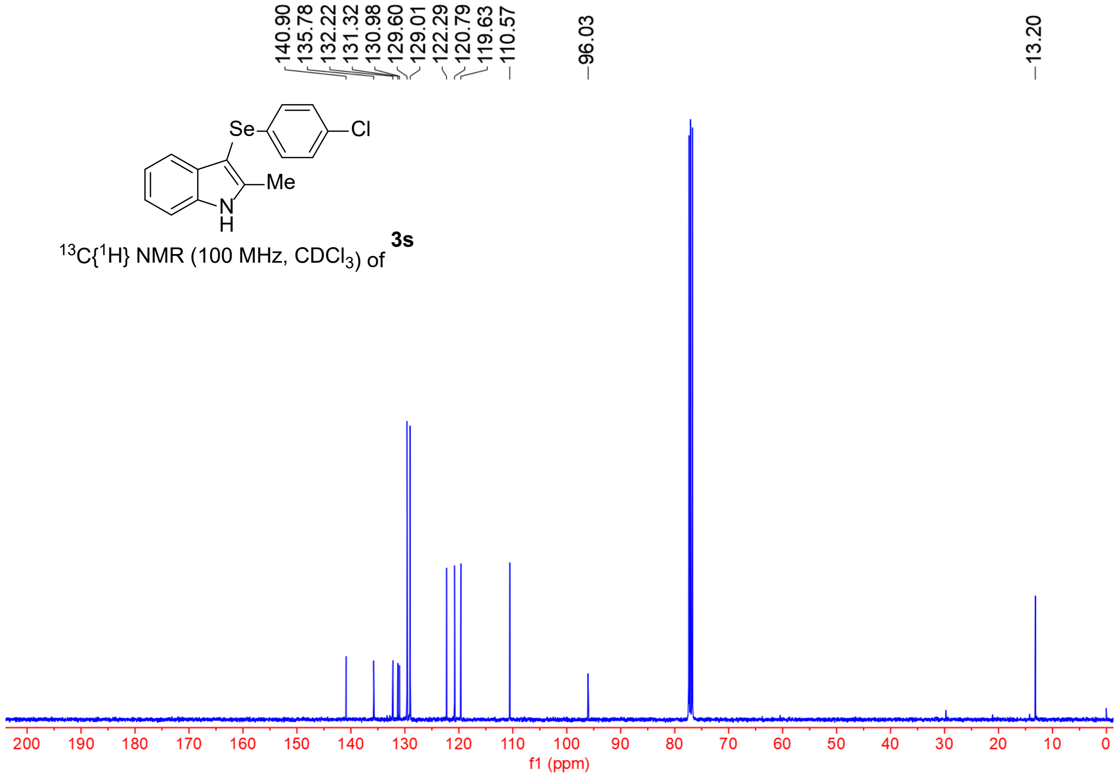

Figure S40: ^{13}C NMR spectrum for compound **3s**

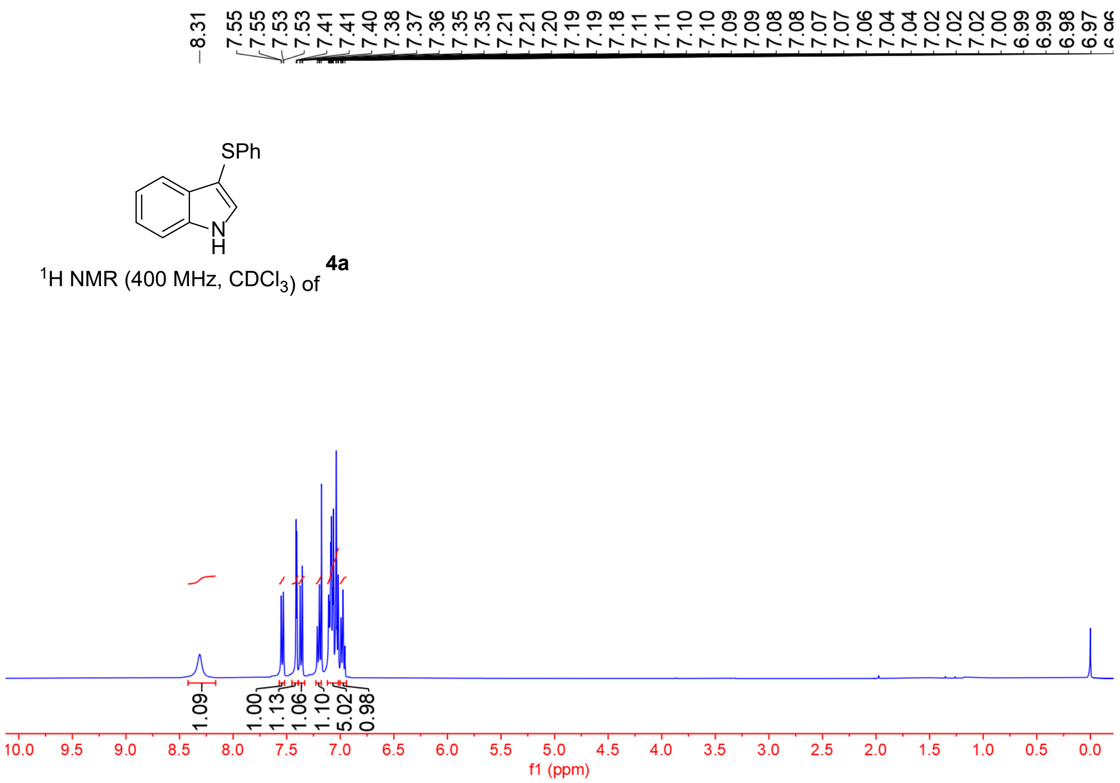

Figure S41: ^{1}H NMR spectrum for compound **4a**

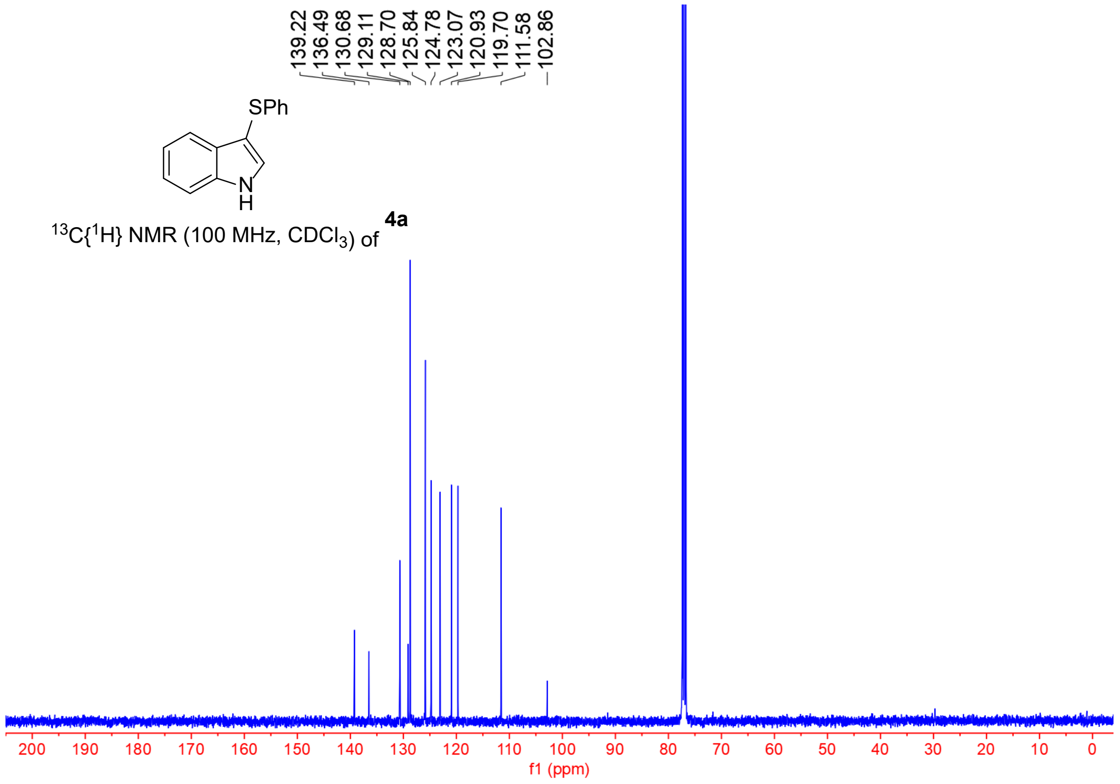

Figure S42: ^{13}C NMR spectrum for compound **4a**

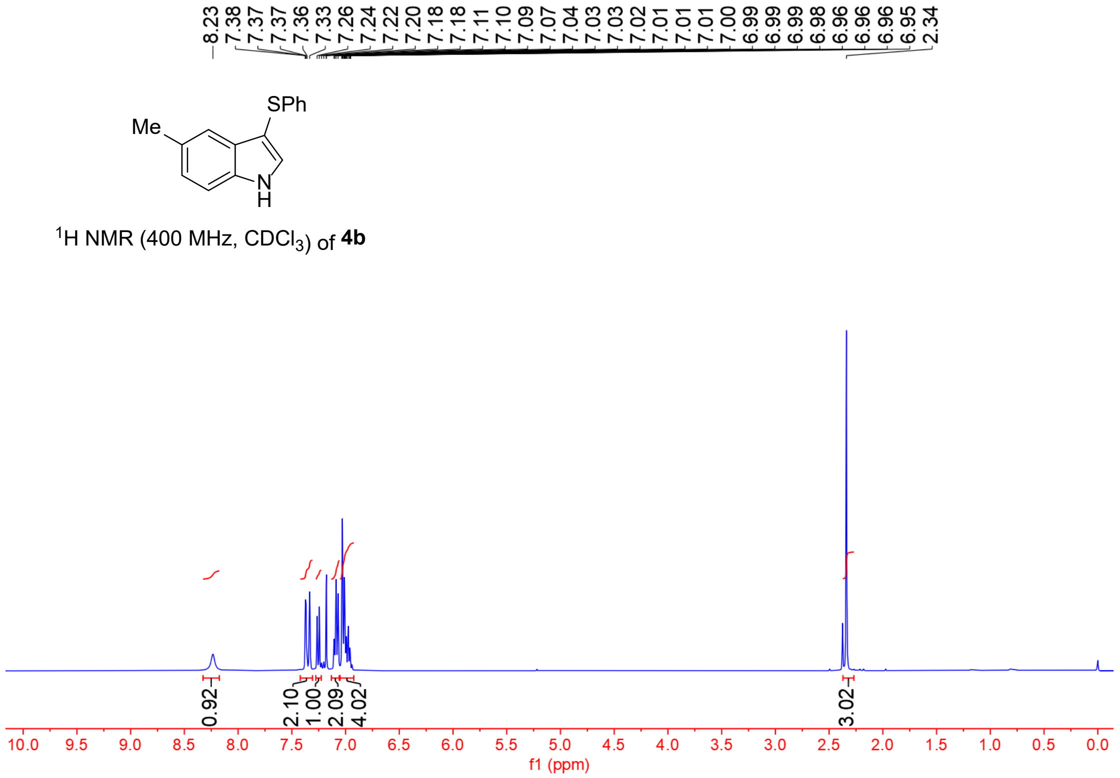

Figure S43: ^{1}H NMR spectrum for compound **4b**

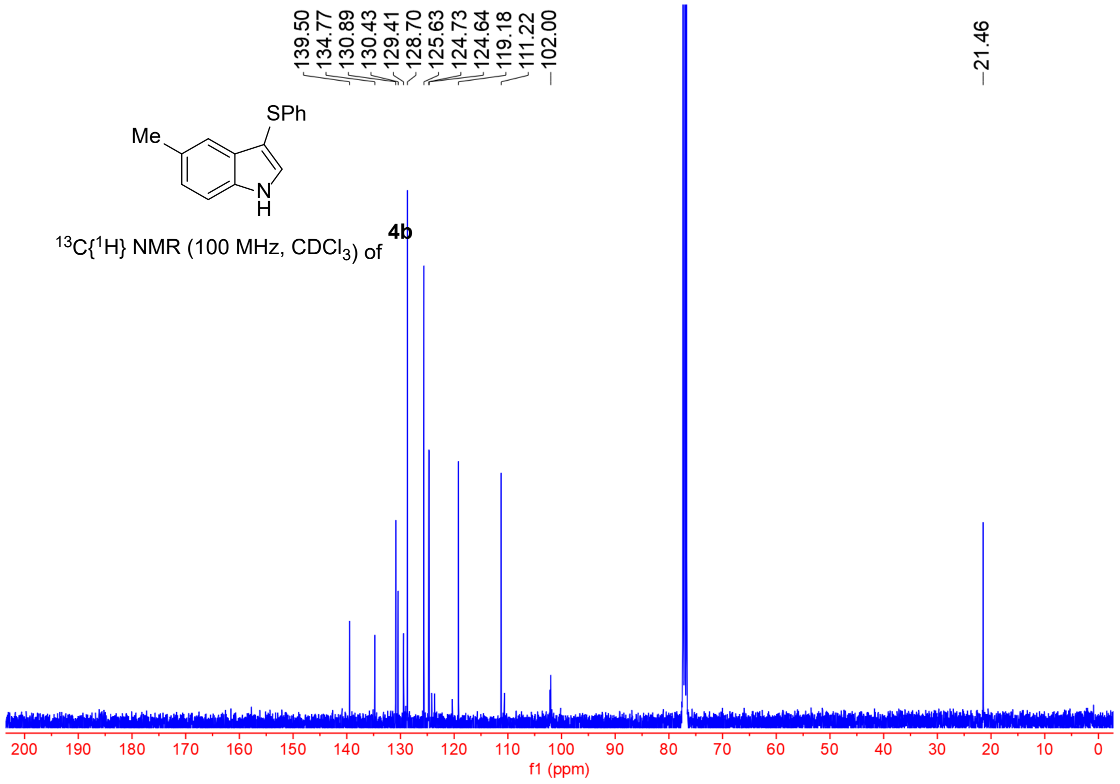

Figure S44: ^{13}C NMR spectrum for compound **4b**

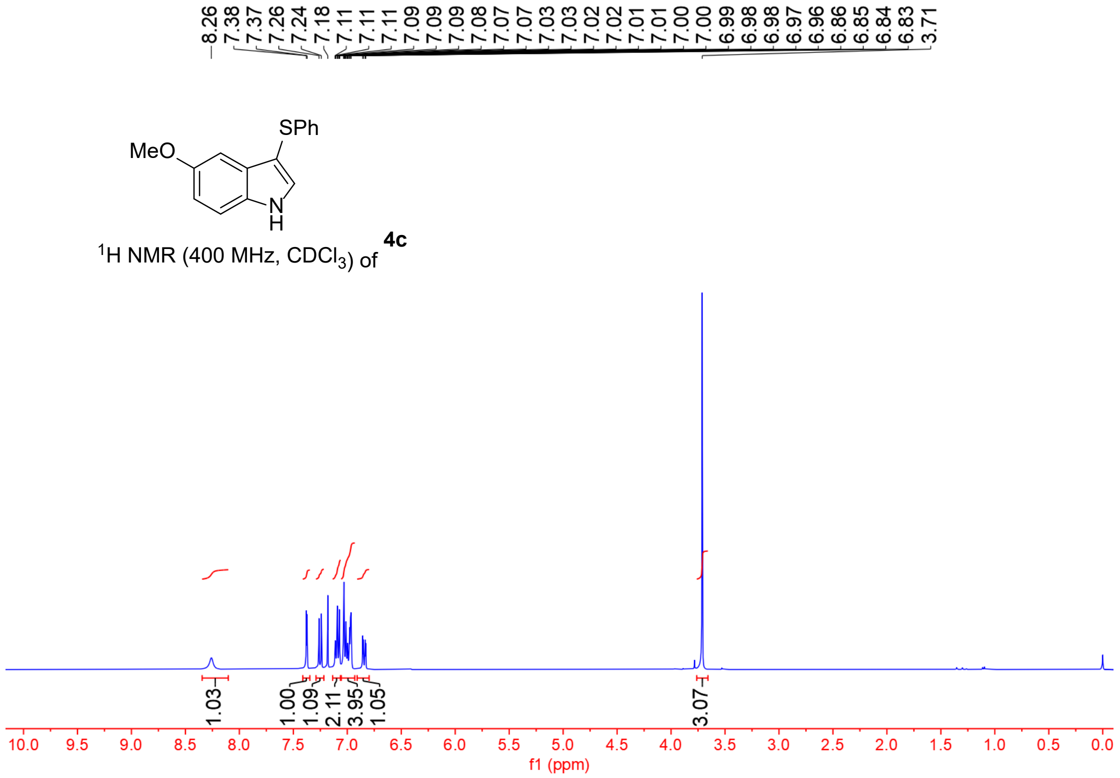

Figure S45: ^{1}H NMR spectrum for compound **4c**

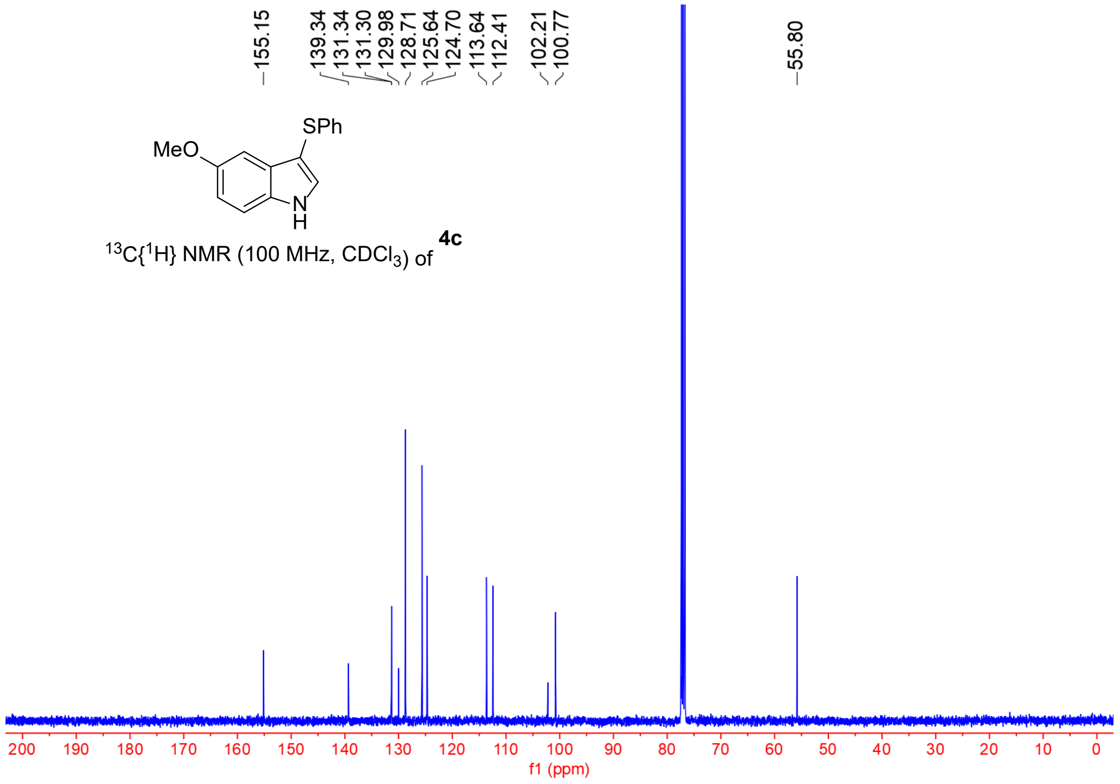

Figure S46: ^{13}C NMR spectrum for compound **4c**

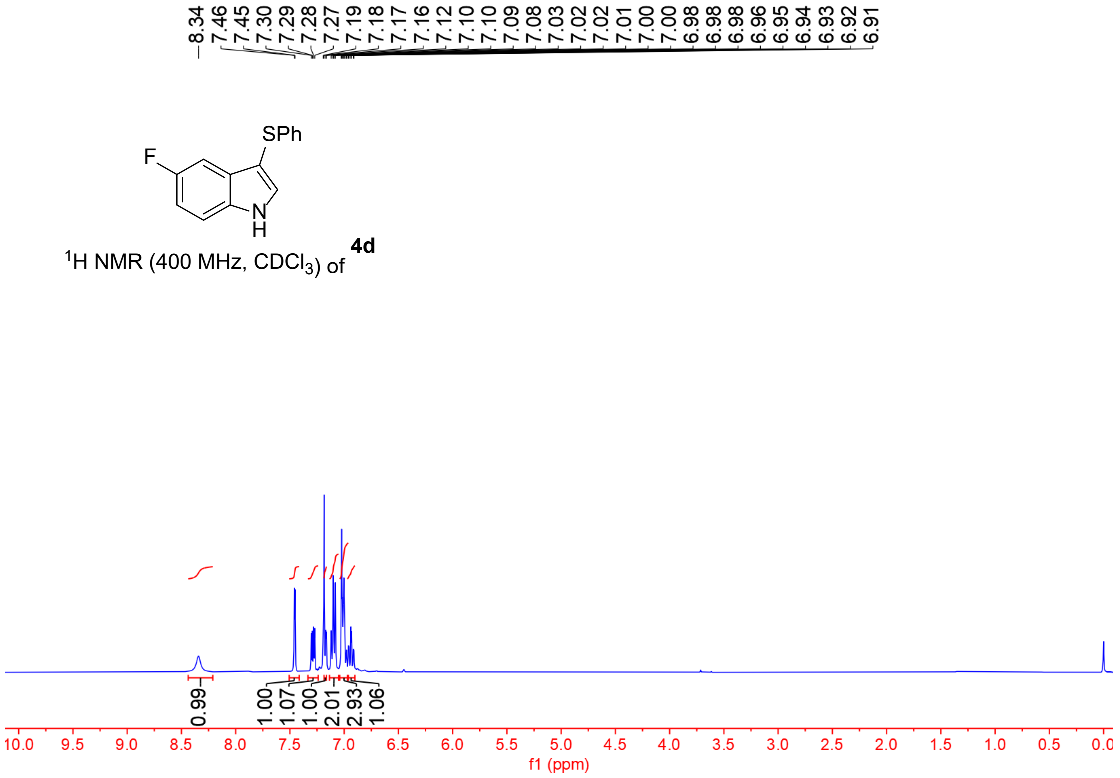

Figure S47: ^{1}H NMR spectrum for compound **4d**

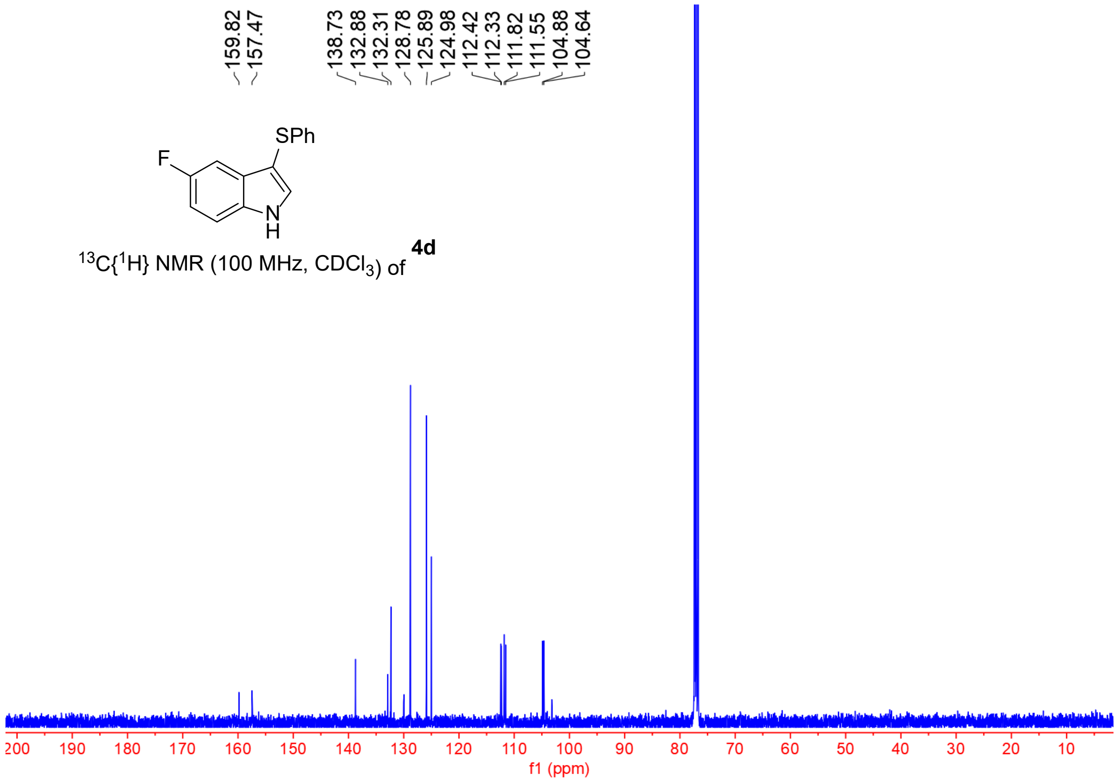

Figure S48: ^{13}C NMR spectrum for compound **4d**

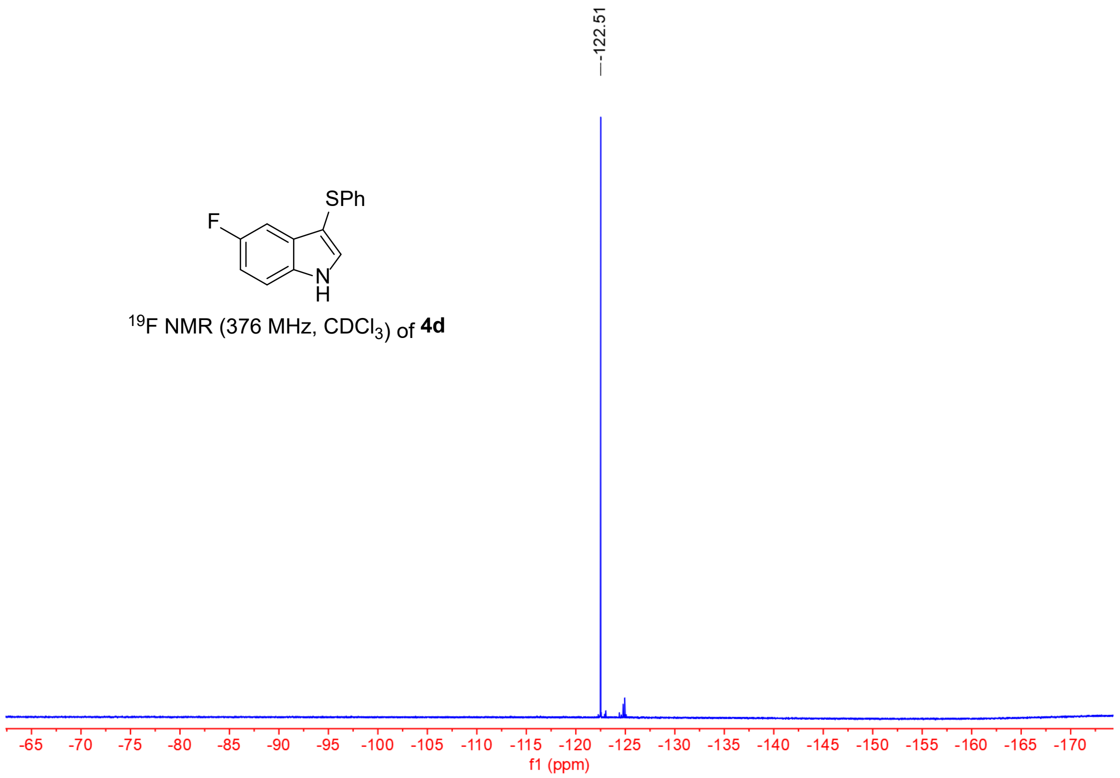

Figure S49: ^{19}F NMR spectrum for compound **4d**

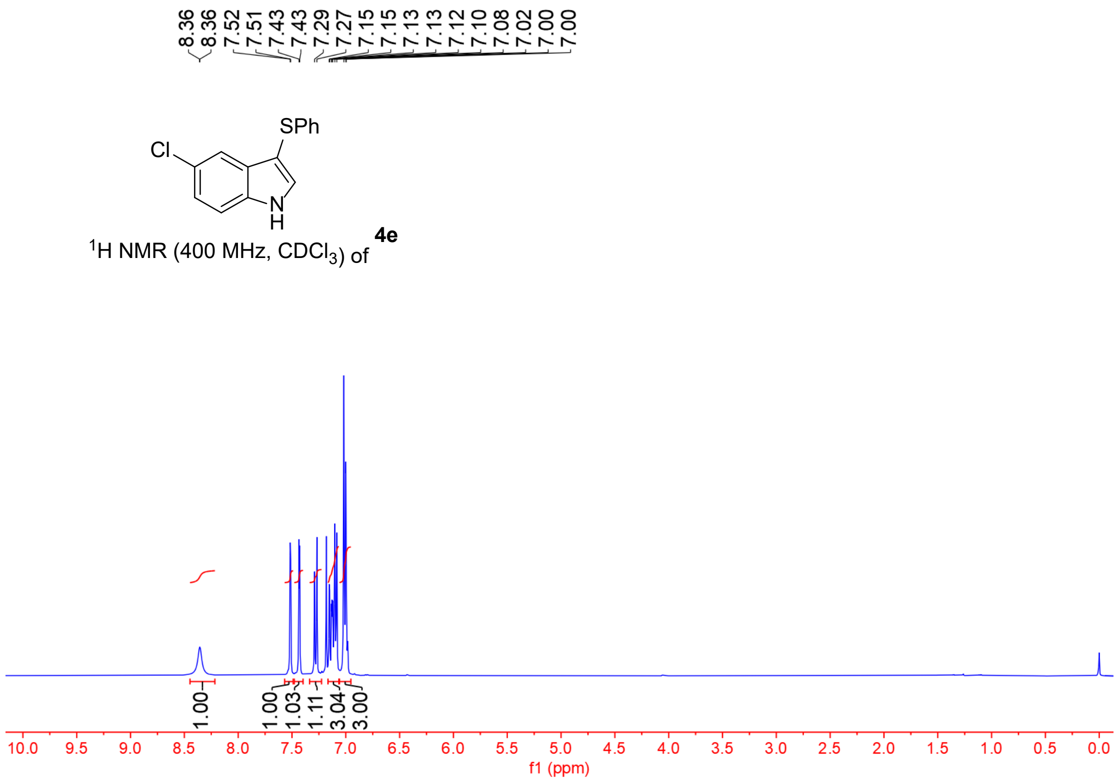

Figure S50: ^{1}H NMR spectrum for compound **4e**

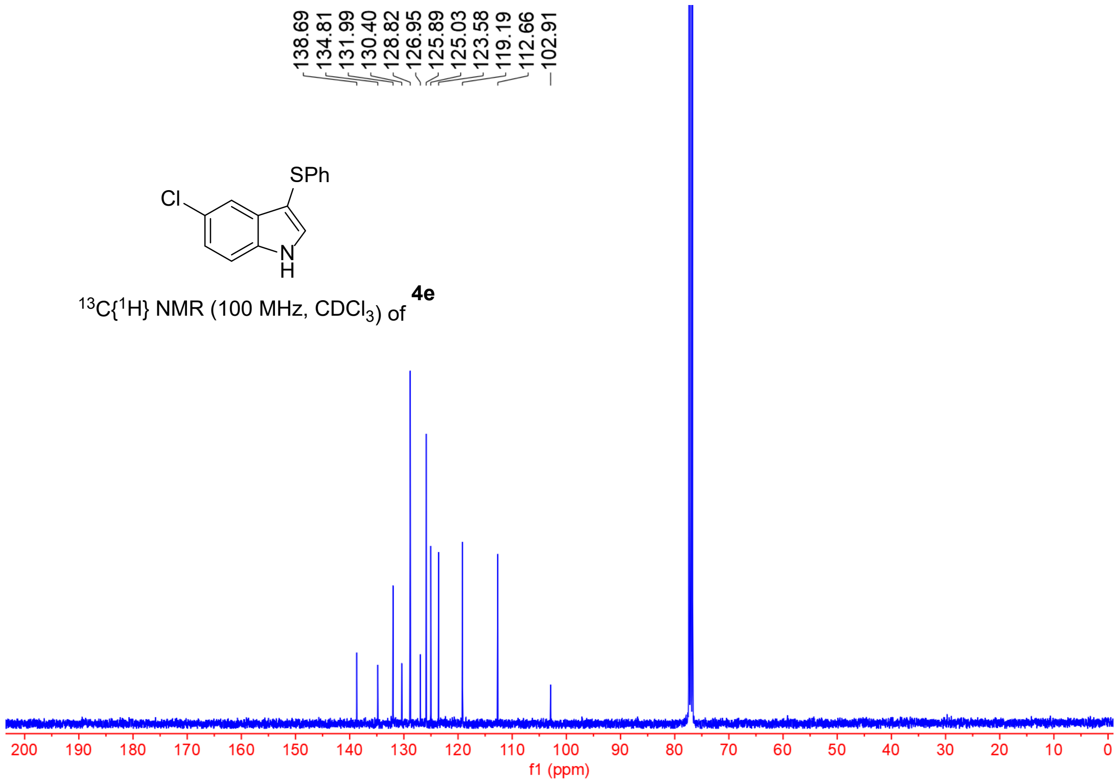

Figure S51: ^{13}C NMR spectrum for compound **4e**

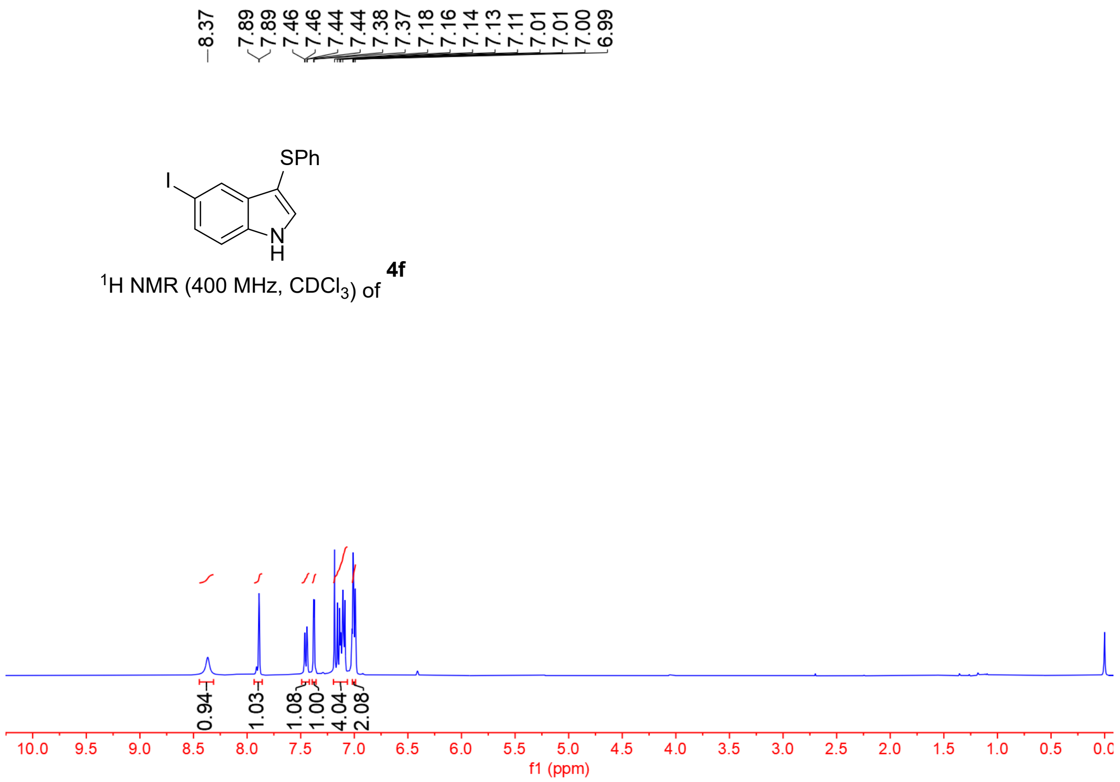

Figure S52: ^{1}H NMR spectrum for compound **4f**

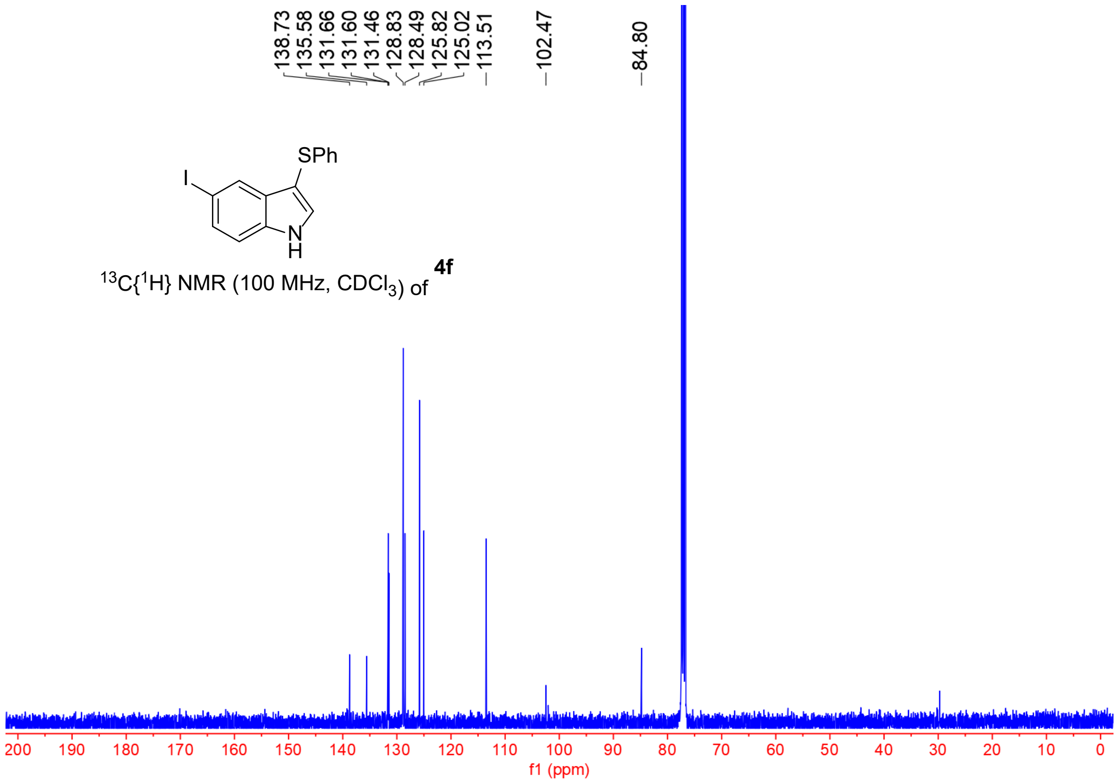

Figure S53: ^{13}C NMR spectrum for compound **4f**

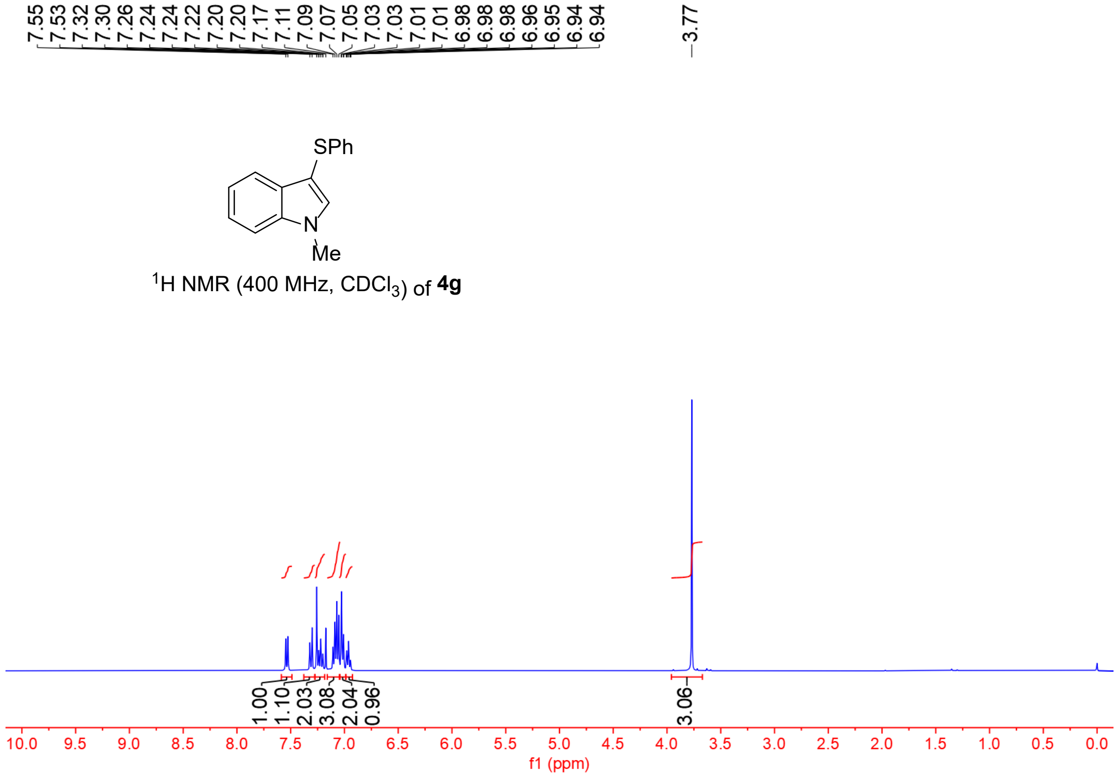

Figure S54: ^{1}H NMR spectrum for compound **4g**

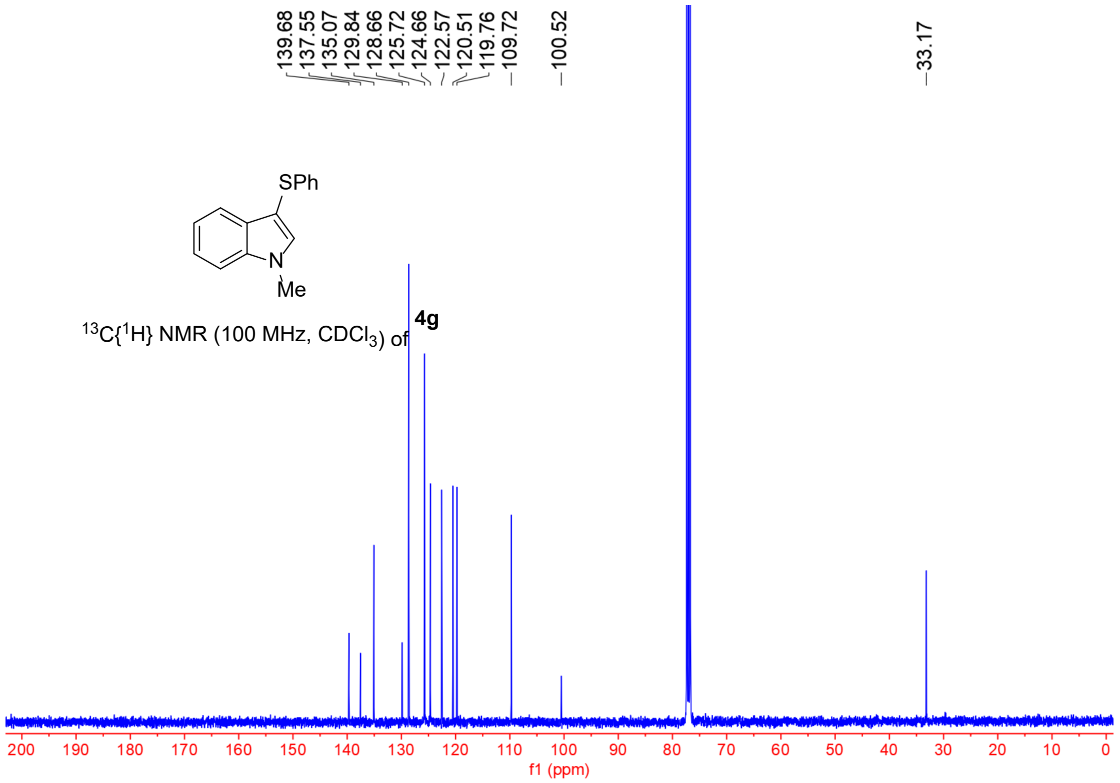

Figure S55: ^{13}C NMR spectrum for compound **4g**

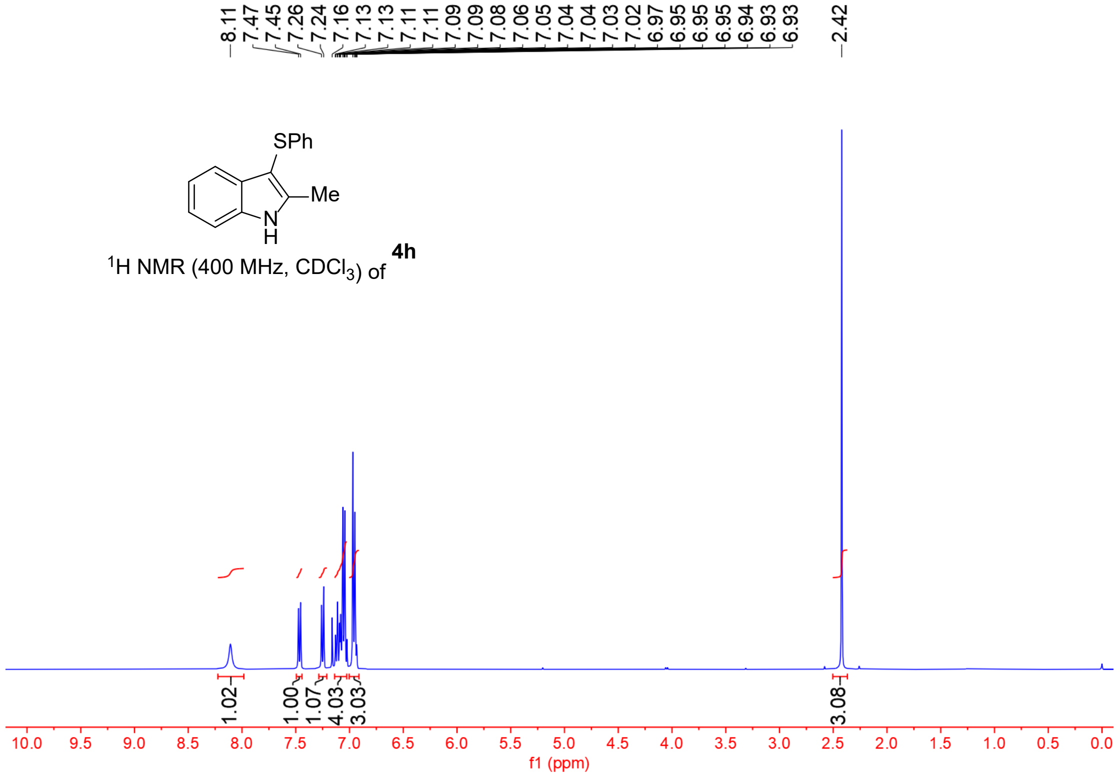

Figure S56: ^{1}H NMR spectrum for compound **4h**

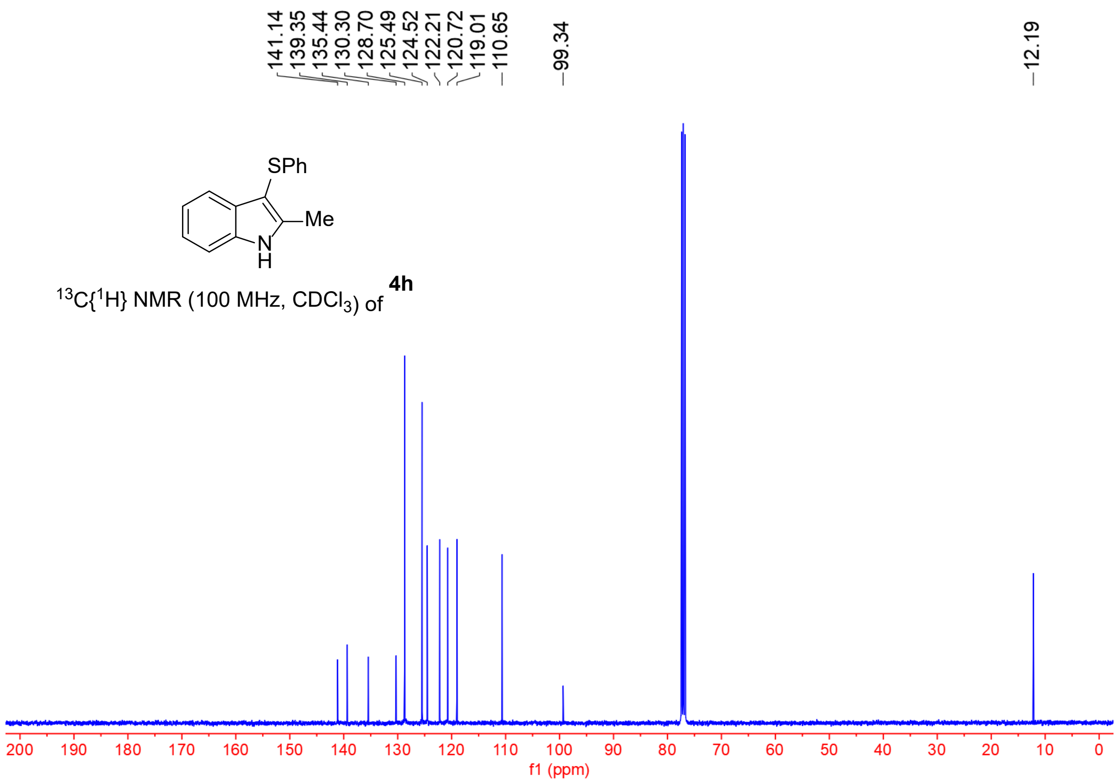

Figure S57: ^{13}C NMR spectrum for compound **4h**

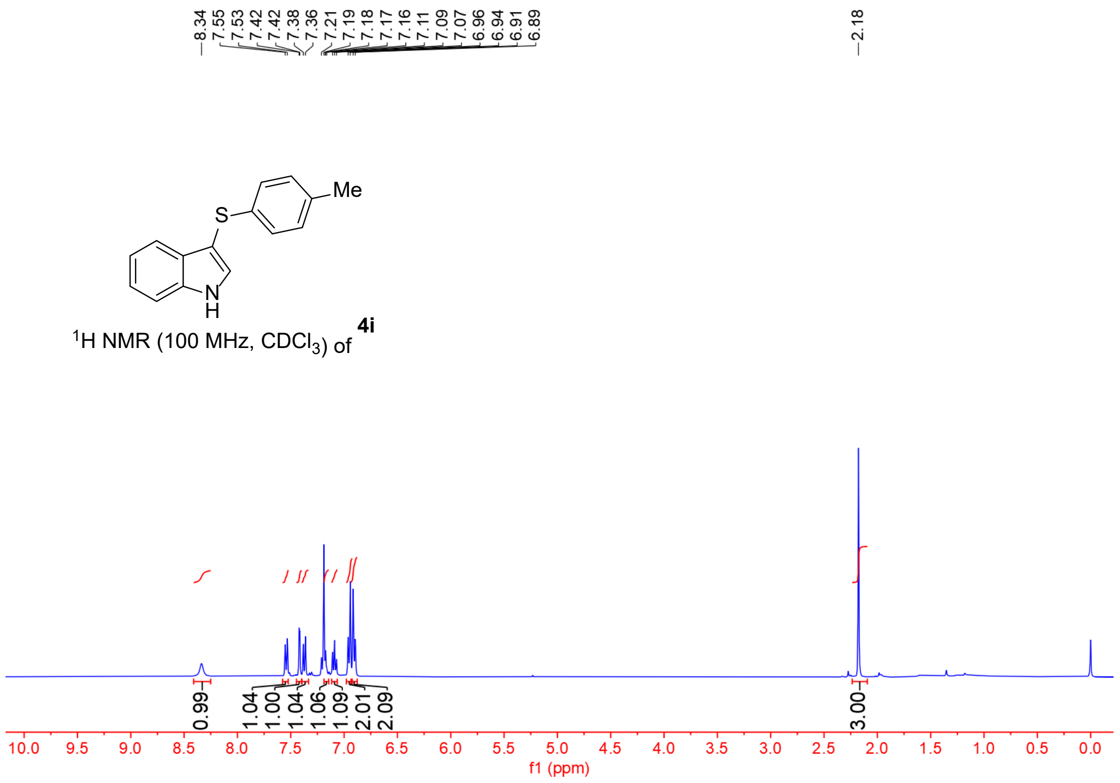

Figure S58: ^1H NMR spectrum for compound **4i**

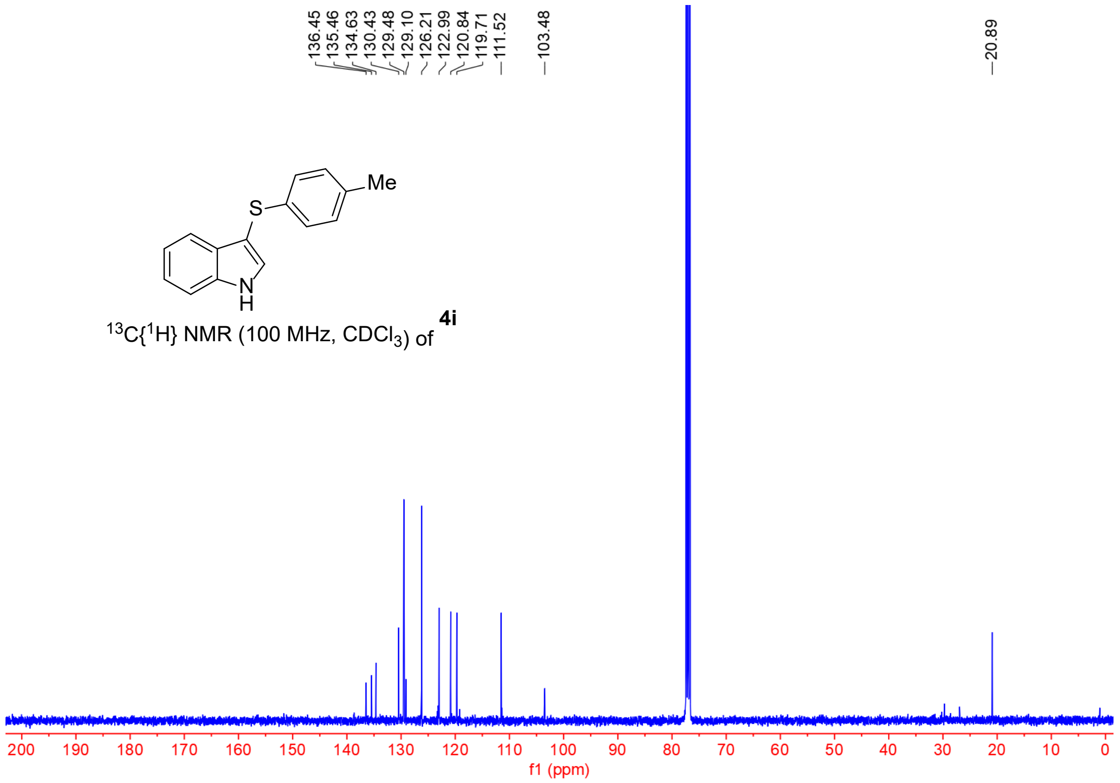

Figure S59: ^{13}C NMR spectrum for compound **4i**

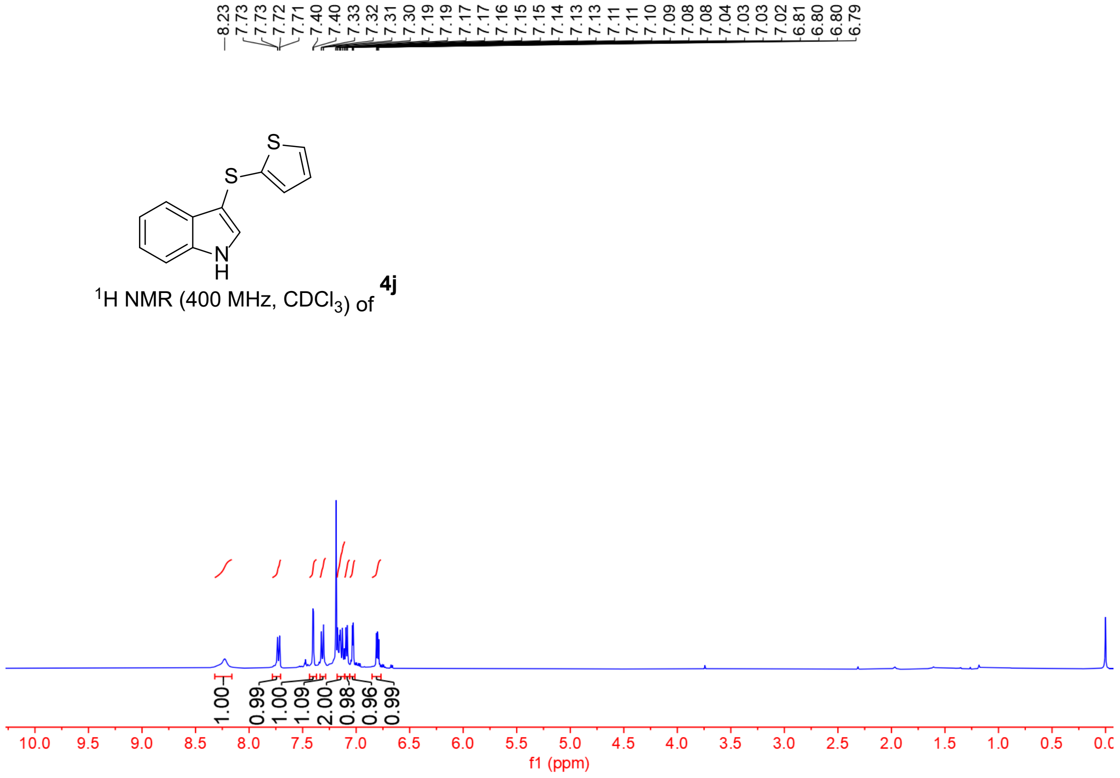

Figure S60: ^{1}H NMR spectrum for compound **4j**

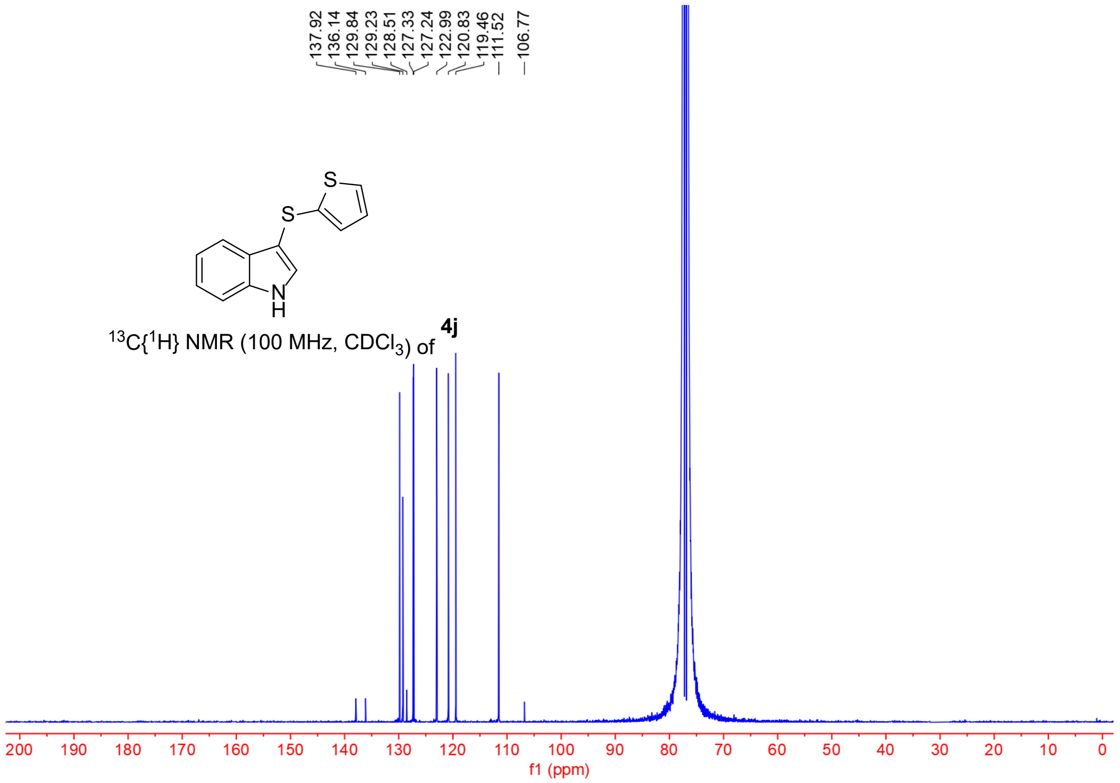

Figure S61: ^{13}C NMR spectrum for compound **4j**

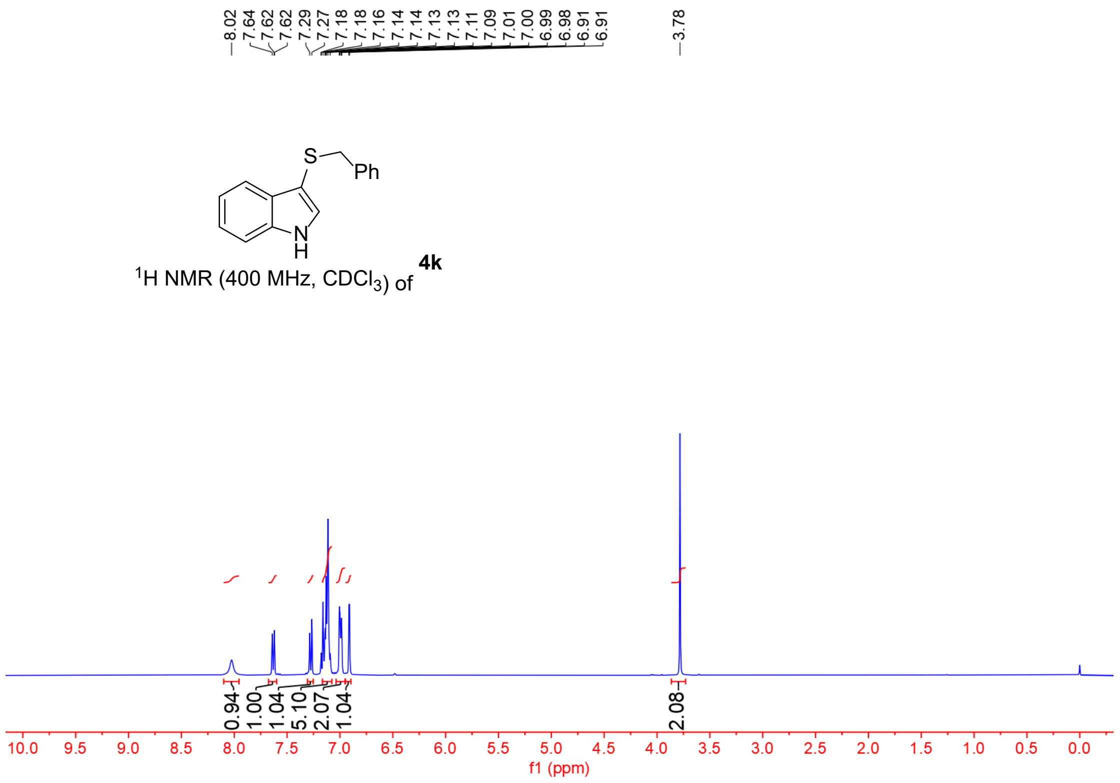

Figure S62: ^{1}H NMR spectrum for compound **4k**

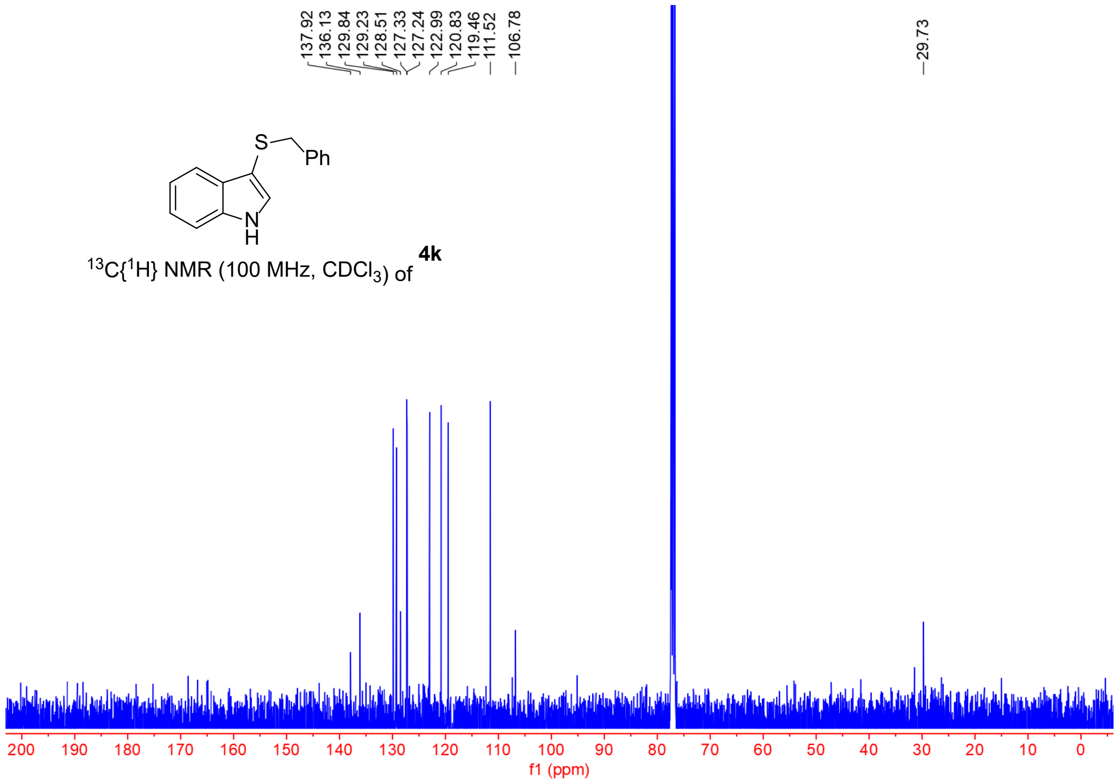

Figure S63: ^{13}C NMR spectrum for compound **4k**

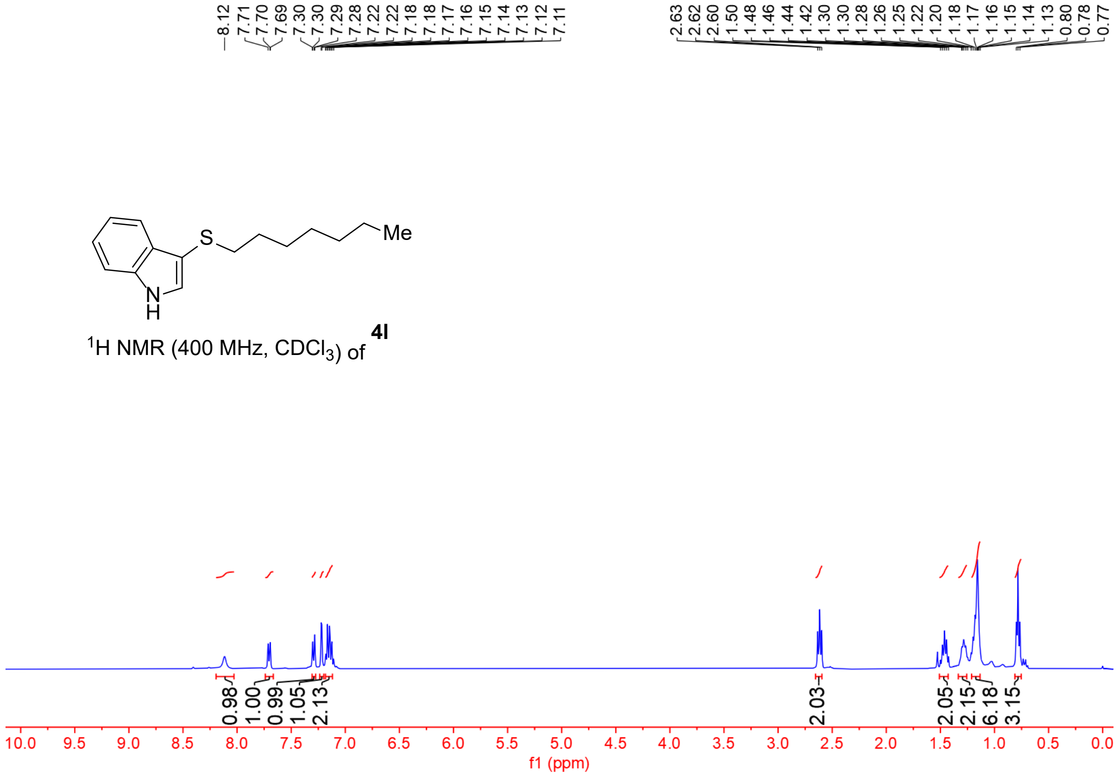

Figure S64: ^1H NMR spectrum for compound **4l**

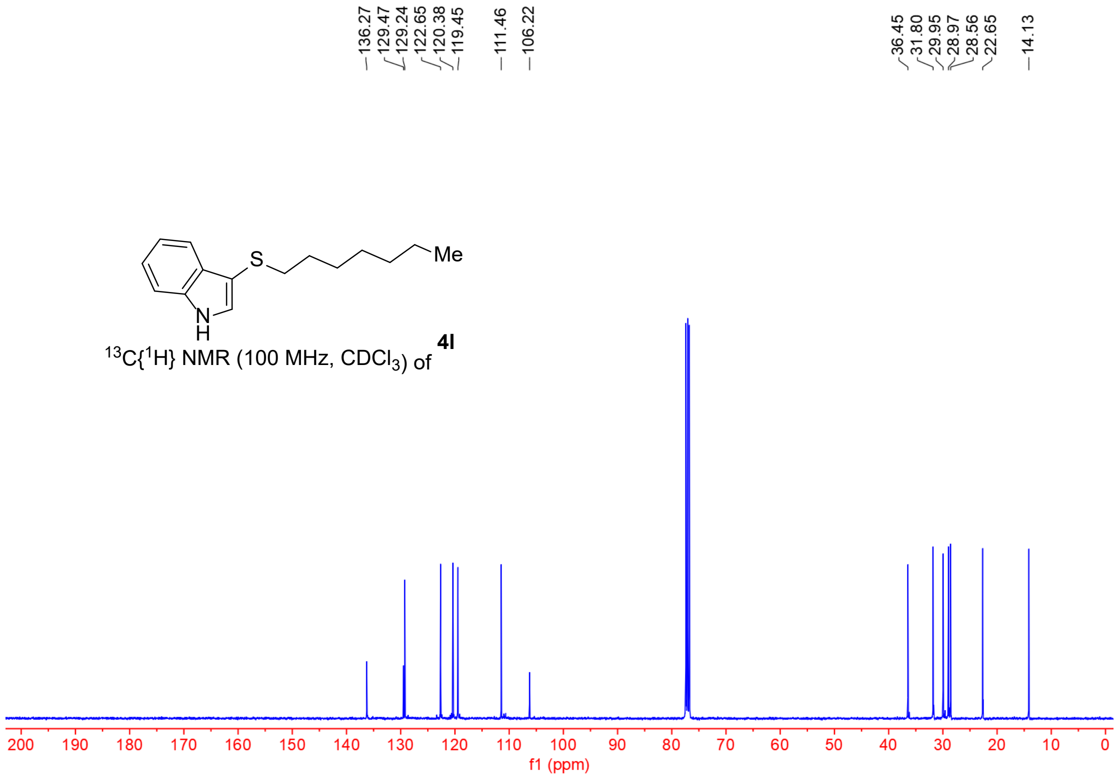

Figure S65: ^{13}C NMR spectrum for compound **4l**
